# Interaction between Conjugative and Retrotransposable Elements in Horizontal Gene Transfer

**DOI:** 10.1371/journal.pgen.1004853

**Published:** 2014-12-04

**Authors:** Olga Novikova, Dorie Smith, Ingrid Hahn, Arthur Beauregard, Marlene Belfort

**Affiliations:** 1 Department of Biological Sciences and RNA Institute, University at Albany, Albany, New York, United States of America; 2 Department of Biomedical Sciences, University at Albany, Wadsworth Center, NYS Department of Health, Albany, New York, United States of America; Fred Hutchinson Cancer Research Center, United States of America

## Abstract

Mobile genetic elements either encode their own mobilization machineries or hijack them from other mobile elements. Multiple classes of mobile elements often coexist within genomes and it is unclear whether they have the capacity to functionally interact and even collaborate. We investigate the possibility that molecular machineries of disparate mobile elements may functionally interact, using the example of a retrotransposon, in the form of a mobile group II intron, found on a conjugative plasmid pRS01 in *Lactococcus lactis*. This intron resides within the pRS01 *ltrB* gene encoding relaxase, the enzyme required for nicking the transfer origin (*oriT*) for conjugal transmission of the plasmid into a recipient cell. Here, we show that relaxase stimulates both the frequency and diversity of retrotransposition events using a retromobility indicator gene (RIG), and by developing a high-throughput genomic retrotransposition detection system called RIG-Seq. We demonstrate that LtrB relaxase not only nicks ssDNA of its cognate *oriT* in a sequence- and strand-specific manner, but also possesses weak off-target activity. Together, the data support a model in which the two different mobile elements, one using an RNA-based mechanism, the other using DNA-based transfer, do functionally interact. Intron splicing facilitates relaxase expression required for conjugation, whereas relaxase introduces spurious nicks in recipient DNA that stimulate both the frequency of intron mobility and the density of events. We hypothesize that this functional interaction between the mobile elements would promote horizontal conjugal gene transfer while stimulating intron dissemination in the donor and recipient cells.

## Author Summary

Mobile genetic elements are segments of DNA that capable of “jumping” within a single DNA molecule, between chromosomes or even between cells. They usually encode the enzymes that mediate their own transfer and integration into new DNA locus. The transfer of mobile genetic elements between cells is known as horizontal gene transfer and it is common in Bacteria. Conjugative plasmids are major means to horizontal gene transfer often carrying a variety of putative virulence factors and antibiotics resistance determinants among and within bacterial species. Thus, conjugative plasmids play a crucial role in the plasticity of the genome, allowing bacteria to adjust readily to new environments. Other mobile elements, such as mobile group II introns, were found to be associated with conjugative plasmids. Here, we demonstrated that mobile group II intron and conjugative plasmid interact promoting gene transfer, and potentially providing a mutual benefit to each other.

## Introduction

Mobile group II introns are remarkable retroelements, based on large catalytic RNAs that encode reverse transcriptase (RT) that is required for their movement [1], [2]. Despite their bacterial origin, they are of interest for their putative ancestral relationship to nuclear spliceosomal introns and their ability to invade DNA and spread by a mechanism similar to non-LTR retrotransposons in metazoans [3]–[6]. Besides being at the nexus of eukaryotic evolution, group II introns often co-exist with other mobile elements in bacteria [7]. The molecular underpinning of one such liason is explored below.

**Figure 1:**
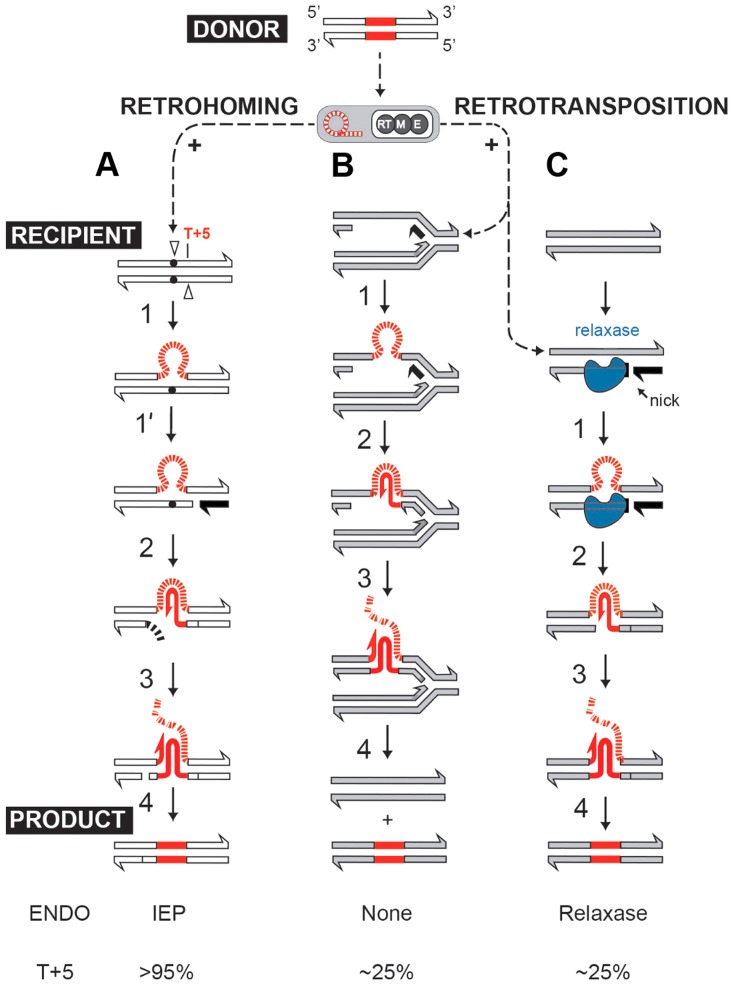
Group II intron retromobility pathways. (**A**) Endonuclease-dependent retrohoming pathway; (**B**) endonuclease independent retrotransposition; (**C**) Model for relaxase-dependent stimulation of retrotransposition. The intron is red (DNA solid lines, RNA hatched lines); the intron RNP is represented by a grey rectangle with a red intron lariat and an IEP with reverse transcriptase (RT), maturase (M) and endonuclease (E) activities. Exons of the donor are white, and that of the recipient are either white (retrohoming) or grey (retrotransposition). Each pathway ends with a product. Black exon fragment represents primer for reverse transcription. In the retrohoming pathway (**A**), dots in the recipient strands represent the homing site where the arrowhead on the top strand is the site of reverse splicing. The arrowhead on the bottom strand is the site of endonuclease cleavage. Endonuclease activity (ENDO) is provided by the IEP (as indicated at the bottom). The conserved residue at position +5 is indicated: T+5 occurs with a frequency of >95% for IEP-dependent cleavage (shown at the bottom). After reverse splicing of the top strand of dsDNA (step 1), cleavage of the bottom strand occurs 9 nt downstream (step 1′). In the retrotransposition pathway (**B**), integration is into ssDNA at a replication fork at an ectopic site (step 1). cDNA synthesis proceeds with the intron as template, using the 3′-OH of the cleaved bottom strand (**A**) or of an Okazaki fragment (**B**) to prime reverse transcription (step 2). No ENDO is necessary in this pathway. Intron degradation and second-strand cDNA synthesis (step 3) is followed by DNA repair to generate the retrohoming or retrotransposition products (step 4). According to data presented in this work, spurious nicking by relaxase provides primers for cDNA synthesis to promote retrotransposition (**C**). In endonuclease-independent pathways (**B**) and (**C**), no preference for T+5 was observed.

Self-splicing group II introns can efficiently insert into intronless alleles by a retrohoming process, or integrate at lower frequencies into ectopic sites by retrotransposition [2], [8], [9]. Both retrohoming and retrotransposition occur by reverse splicing into DNA, where the intron RNA is copied into cDNA by the RT activity of the intron-encoded protein (IEP). In the high-efficiency retrohoming reaction, integration is into dsDNA and the primer for cDNA synthesis is provided by a nick introduced by the IEP's endonuclease activity [8], [10] (Fig. 1A). In low-frequency retrotransposition to ectopic sites, integration is predominantly into ssDNA and primers can be provided by Okazaki fragments at replication forks [11], [12] (Fig. 1B), leading to broad intron dispersal.

**Figure 2:**
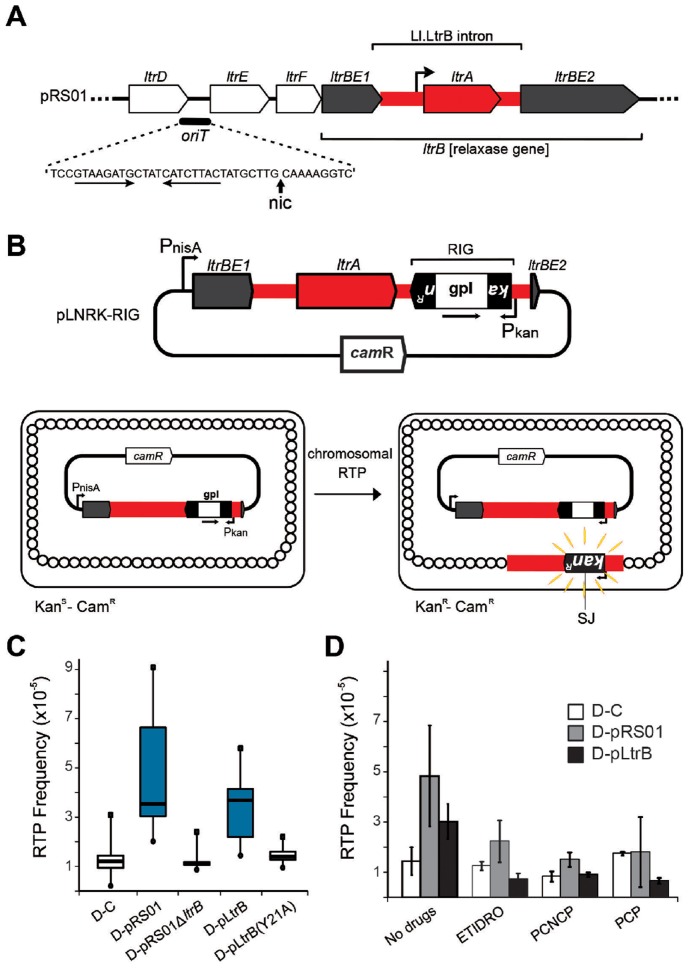
Relaxase-stimulated Ll.LtrB intron retrotransposition. (**A**) Relaxase locus of the *Lactococcus lactis* conjugative plasmid pRS01 includes relaxase gene (*ltrBE1* and *ltrBE2*) interrupted by the intron (Ll.LtrB, red) within the operon encoding auxiliary factors for conjugation (*ltrD*, *ltrE*, and *ltrF*) and origin of transfer *oriT*. The IEP, LtrA, is necessary for splicing and retromobility. The *oriT* site includes inverted repeats (horizontal arrows) and a proposed nick site (*nic*) [29] verified in this work. (**B**) The intron donor plasmid pLNRK-RIG carries the intron (red) flanked by exons (*ltrBE1* and *ltrBE2*) under a nisin-inducible promoter (P_nisA_). The intron is interrupted by a *r*etromobility *i*ndicator *g*ene (RIG) with the *kan^R^* gene inserted in the anti-transcriptional orientation to the intron, and carrying its own promoter (P_kan_). The *kan^R^* gene is interrupted by a self-splicing group I intron (gpI) in the same orientation as Ll.LtrB (arrow). Reconstitution of the *kan^R^* gene with formation of a characteristic splice junction (SJ) and kanamycin resistance is possible only through reverse transcription of an RNA intermediate that lost the group I intron during retrotransposition. (**C**) Box-plots for retrotransposition assays in cells expressing (blue) or lacking relaxase, performed in control strain (D-C), or in the strains containing the indicated plasmids described in the text. A significant difference between strains with relaxase D-pRS01 (two-tailed paired t test, p = 3.4×10^−5^) and D-pLtrB (p = 1.1×10^−5^) and control strain D-C was observed. (**D**) Results of retrotransposition assays in the presence of relaxase-specific inhibitors. Retrotransposition was assayed in cells treated with etidronic acid (ETIDRO), iminobis (methylphosphonic acid) (PCNCP), and methylenediphosphonic acid (free acid) (PCP) (10 mM) [21]. The error bars represent the standard errors. No statistically significant differences could be found in any of the three drug treatments between control strain D-C and strains with relaxase D-pRS01 (two-tailed paired t test, p(ETIDRO) = 0.12, p(PCNCP) = 0.22, and p(PCP) = 0.96) and D-pLtrB (p(ETIDRO) = 0.10, p(PCNCP) = 0.72, and p(PCP) = 0.09).

Curiously, many group II introns reside on bacterial mobile elements including plasmids. Examples are provided by conjugative plasmid pRS01 in *Lactococcus*, virulence plasmid pX01 in *Bacillus*, and *Pseudomonas*-derived toluene-catabolic self-transmissible plasmid pDK1 [13], [14]. The pRS01 plasmid, a broad-host-range conjugative element, mediates high-frequency transfer of the genes encoding lactose utilization among lactococci [15]. In bacteria, conjugation is one of the most common ways to increase genome plasticity via horizontal gene transfer. The mobile conjugative plasmids carry, among others, the genes encoding proteins necessary for conjugative DNA transfer, including a relaxase enzyme which is necessary for the initiation of conjugation. The relaxase nicks the plasmid DNA at the origin of transfer (*oriT*) and ssDNA is transferred to the recipient in a site- and strand-specific manner during conjugation [16]–[18]. This reversible reaction creates a covalent linkage between the active-site tyrosyl hydroxyl oxygen and ssDNA 5′-phosphate, yielding a 3′- OH, which can later act as a nucleophile to reverse the transesterification and release the relaxase. Interestingly, the Ll.LtrB group II intron resides within the relaxase *ltrB* gene of pRS01 plasmid (Fig. 2A), where intron splicing is required for expression of the functional LtrB relaxase protein, which initiates conjugation [13]. Moreover, intron retrotransposition is stimulated in the recipient cell by conjugation [19], suggesting either that the transfer process or factors encoded by the conjugative plasmid aid intron mobility. Thus, conjugative plasmids not only transfer the intron between bacterial cells and even across genera and species [19], [20], but aid dissimination of the intron in the recipient.

In the present study we report on the discovery that the conjugative relaxase, LtrB, promotes Ll.LtrB group II intron retrotransposition. To provide a mechanistic basis for enhanced retrotransposition, biochemical experiments were performed with the purified relaxase and the distribution of the retrotransposition events reported by a *r*etromobility *i*ndicator *g*ene (RIG) was explored. To this end we developed a targeted sequencing approach specifically for retrotransposition insertion sites, called RIG-Seq. Together, the data show the dynamic interaction between two different mobile elements: Intron splicing is essential for active relaxase expression required for conjugation; in turn, relaxase nicks recipient DNA to provide 3′ ends that prime and thereby stimulate retrotransposition to novel sites. Moreover, given that conjugation facilitates intron retrotransposition in both donor and recipient cells, one can infer that this co-habitation of different mobile elements promotes gene transfer and genome plasticity.

## Results

### Frequencies of retrotransposition are increased in the presence of active relaxase

To study the effect of the conjugative plasmid pRS01 on mobility of the Ll.LtrB group II intron, we used a *kan^R^* RIG cassette where kanamycin resistance is acquired after splicing of a group I intron from an RNA intermediate [11] (Fig. 2B). Whereas retrotransposition frequencies in the control donor strain were ∼1×10^−5^/recipient (strain D-C), the presence of the conjugative plasmid pRS01 stimulated retrotransposition levels to 4.7×10^−5^ on average (median 3.6×10^−5^; strain D-pRS01) (Fig. 2C; for strains see Table 1). To test if the relaxase itself was the stimulatory factor, a deletion of the relaxase gene from pRS01 was tested (strain D-pRS01Δ*ltrB*). The retrotransposition frequency dropped to ∼1×10^−5^, the same as that of the D-C control. Additionally, with relaxase expression from pLtrB::Ll.LtrB (strain D-pLtrB), retrotransposition increased to 3.2×10^−5^ on average (median 3.7×10^−5^; Fig. 2C). This elevation in retrotransposition frequency is not due to effects of donor plasmid pLNRK-RIG copy number, which is slightly depressed in cells carrying pRS01 and pLtrB (Figure S2A in Text S1), resulting in an underestimate of stimulatory effects by as much as a factor of 2.

**Table 1:** Bacterial strains and plasmids.

Bacteria	Strain	Relevant characteristics; comments	Reference
*L. lactis*	IL1403	plasmid free; recipient strain, strain for all RTP assays; sequenced genome is available (GenBank: NC_002662)	[53]
*L. lactis*	DM2036	donor of pRS01::pTRK28	[40]
*E. coli*	DH5α	F^−^ *endAI recAl hsdRl7* (rK^−^ mK^−^) *deoR supE44 thi-J gyrA96 relA*	Gibco-BRL
*E. coli*	TOP10	F^−^ *mcr*A Δ(*mrr*-*hsd*RMS-*mcr*BC) Φ80*lac*ZΔM15 Δ*lac*X74 *rec*A1 *ara*D139 Δ(*ara leu*) 7697 *gal*U *gal*K *rps*L (Str^R^) *end*A1 λ^−^; expression strain	Invitrogen
*E. coli*	TOP10F′	F′{*lac*Iq, Tn*10*(TetR)}c *mcr*A Δ(*mrr*-*hsd*RMS-*mcr*BC) Φ80*lac*ZΔM15 Δ*lac*X74 *rec*A1 *ara*D139 Δ(*ara leu*) 7697 *gal*U *gal*K *rps*L (Str^R^) *end*A1 *nup*G; ssDNA production	Invitrogen
**Plasmids**			
pLNRK-RIG	D-C	*L. lactis*/*E. coli* shuttle vector contains nisin-inducible promoter, *nisR nisK*, Cam^R^, RIG cassette, intron donor	[11], [40]
pCJK21		nisin-inducible expression vector, Erm^R^, Spc^R^	[41]
pRS01::pTRK28	D-pRS01	co-integrant of pRS01 and pTRK28, Erm^R^	[40]
pDL278		shuttle vector, Spc^R^	[54]
pBSΔ*ltrB*::*tet*		pBlueScript with *ltrB* partially replaced with *tet* gene, Amp^R^, Tet^R^	Present study; pBlueScript – Agilent Technologies
pRS01Δ*ltrB*::*tet*	D-pRS01Δ*ltrB*	pRS01::pTRK28 with *ltrB* replaced with *tet* gene, Erm^R^, Tet^R^	Present study
pLtrB::Ll.LtrB	D-pLtrB	full-length intron-containing *ltrB*, cloned into *Spe*I/*Sph*I sites of pCJK21	Present study
pLtrB(Y21A)::Ll.LtrB	D-pLtrB(Y21A)	Full-length intron-containing *ltrB*, cloned into *Spe*I/*Sph*I sites of pCJK21, Y21A mutant	Present study
pLtrB(Y21F)::Ll.LtrB		Y21F *ltrB* mutant	Present study
pLtrB(S117R)::Ll.LtrB		S117R *ltrB* mutant	Present study
pLtrB(S117A)::Ll.LtrB		S117A *ltrB* mutant	Present study
pLtrB(H159R)::Ll.LtrB		H159R *ltrB* mutant	Present study
pLtrB(H159A)::Ll.LtrB		H159A *ltrB* mutant	Present study
pLtrB(H161R)::Ll.LtrB		H161R *ltrB* mutant	Present study
pLtrB(H161A)::Ll.LtrB		H161A *ltrB* mutant	Present study
pCY20		full-length intronless *ltrB*, cloned into *Spe*I/*Sph*I sites of pCJK21	[29]
pBAD33		expression vector, arabinose-inducible promoter; Cam^R^	[50]
pLtrB-HIS6		full-length intronless *ltrB*, cloned into pBAD33, N-end HIS-tagged	Present study
pLtrB(Y21A)-HIS6		full-length intronless *ltrB*, cloned into pBAD33, Y21A mutant; N-end HIS-tagged	Present study
pON*oriT1*		*oriT* from pRS01 cloned into pGEM, Amp^R^	Present study
pON*oriT2*		*oriT* from R388 cloned into pGEM, Amp^R^	Present study
pON*LLglnP*-R		fragment of *glnP* from *L. lactis* IL1403 cloned in reverse orientation into pGEM, Amp^R^	Present study

Mutation of the putative catalytic tyrosine (Y21 in LtrB relaxase) to alanine (strain D-pLtrB(Y21A)) resulted in reduction in retrotransposition to control levels (Fig. 2C; Figure S1 in Text S1). Mutants in non-catalytic active site residues had a lesser effect on retrotransposition (Figure S1 in Text S1). To further investigate the effect of relaxase on retrotransposition, we used several bisphosphonates, etidronic acid (ETIDRO), imino-bis(methylphosphonic acid) (PCNCP), and methylenediphosphonic acid (PCP), which are specific inhibitors of relaxase [21]. Elevated retrotransposition levels in D-pRS01 and D-pLtrB strains were reduced 2- to 3-fold by all three relaxase inhibitors (Fig. 2D).

### Relaxase promotes the diversity of chromosomal retrotransposition events

**Figure 3:**
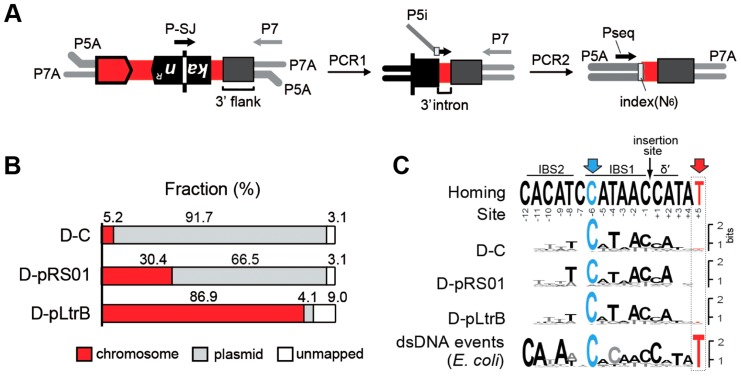
Mapping of intron insertion sites with RIG-seq. (**A**) RIG-seq amplification scheme. High-throughput targeted sequencing of insertion loci was based on the generation of splice junction (SJ) sequence of the *kan^R^* gene during retrotransposition. After ligation of P5A and P7A, Illumina-specific adapters, two tandem PCRs were used to amplify 3′ flanks of retrotransposition events in Illumina libraries: a specific SJ primer (P-SJ) with the P7 primer in the first PCR; then library-specific primers (P5i) carrying library-specific indices with the P7 primer in the second PCR. Pseq, sequencing primer. (**B**) Replicon preferences. Mapping of the Illumina libraries demonstrated a marked preference for intron insertion into the donor plasmid pLNRK-RIG in control D-C strain (Figure S2 in Text S1); the overwhelming majority of the reads for D-pLtrB was mapped to the chromosome. (**C**) Insertion site sequence logos compared to the homing-site sequence. The logos were generated based on multiple sequence alignments of retrotransposition events. The homing site sequence with intron-binding sites (IBS1 and IBS2) and Δ′, required for reverse splicing, is shown on the top. The most conserved residue C-6 is highlighted in blue. The T+5 is shown in red and represents the nucleotide indicative of endonuclease-dependent events. The low frequency of T+5 suggests that endonuclease-independent pathways into ssDNA dominate in *L. lactis* IL1403. The logo for endonuclease-dependent dsDNA events identified in *E. coli* is shown for comparison at the bottom [24].

We next asked whether the presence of relaxase affects the specificity and distribution of the intron retrotransposition events. We developed a strategy for targeted high-throughput detection of retrotransposed introns, called RIG-Seq, based on the Ll.LtrB RIG construct (Fig. 3A). The unique splice junction (SJ) sequence in the *kan^R^* gene, from which the group I intron is removed during retromobility, is present in all retrotransposition events but not in the original intron donor (Fig. 2B). This sequence can be used as the landmark for primer binding to detect exclusively retrotransposition events. Libraries for sequencing were constructed after selection of Kan^R^ retrotransposition events for the control strain D-C and the two relaxase-containing strains D-pRS01 and D-pLtrB. The sequences containing the 3′ flanking genomic sequence were analyzed for each library (Tables S1–S4 in Text S1, where Table S1 represents the summary of multiple sequence alignments for three strains with sequence listings in Tables S2–S4 in Text S1).

Initial mapping of the sequenced fragments showed that many of the intron insertions were in the RIG donor plasmid rather than the chromosome (Fig. 3B and Figure S2 in Text S1). This result is consistent with our previous report that retrotransposition of Ll.LtrB intron occurs frequently into the donor plasmid, likely because of replication-coupled intron integration into ssDNA and the high number of forks per unit of plasmid DNA [22]. Strikingly, whereas only 5.2% of reads were mapped to the chromosome for the control D-C strain, 30.4% of the reads were chromosomal in the presence of pRS01, and 86.9% of the reads were chromosomal when the D-pLtrB strain expressed relaxase alone, suggesting the ability of relaxase to facilitate intron retromobility into the chromosome (Fig. 3B).

**Figure 4:**
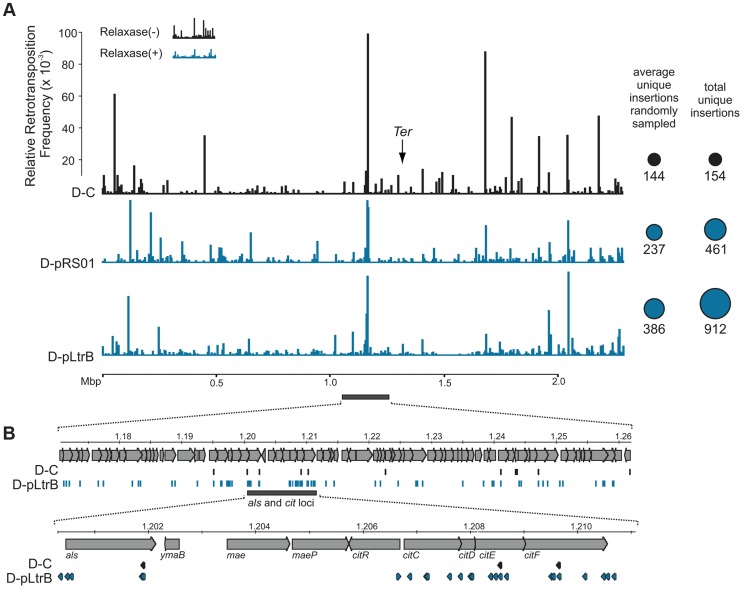
Mapping of retrotransposition events along *L. lactis* chromosome. (**A**) Mapping of sites. Randomly sampled libraries of the reads for D-C, D-pRS01 and D-pLtrB were mapped along the *L. lactis* IL1403 chromosome [25]. For sequences see Tables S2–S4 in Text S1. The relative retrotransposition frequencies are represented by vertical bars: black, D-C strain without relaxase; blue, D-pRS01 and D-pLtrB strains with relaxase. The average number of unique chromosomal insertion points in randomly sampled libraries and the total number of unique chromosomal insertion points in the libraries are shown by spheres (right) with the area of each sphere corresponding to the value indicated. The putative location of *Ter* is shown by arrow [25]. (**B**) High density of the events. The locus ∼60 Kbp upstream of the putative *Ter* is shown in details. Multiple retrotransposition events were observed in the D-pLtrB strain (blue) and less in control strain D-C (black). The *als* and *cit* loci have had the highest density of events in the D-pLtrB strain, while only a few were identified in control strain D-C.

We further established that there are no notable differences in the consensus sequences for the insertion sites among relaxase-positive and -negative libraries, indicating universal underlying mechanisms for retrotransposition in *L. lactis* under these different circumstances (Fig. 3C and Table S1 in Text S1). A distinguishing feature between invasion into dsDNA and ssDNA pathways is a T residue at position +5 relative to the insertion point (Fig. 1A and Fig. 1B). T+5 is required for IEP endonuclease-mediated nicking of the bottom strand in the dsDNA pathway. We therefore performed comparative analysis of the flanking sequences upstream and downstream of the intron insertions [23], [24]. Multiple sequence alignment profiles for each library supported the predominantly ssDNA nature of the insertions because the frequency of the T+5 appeared to be low (∼25%) (Fig. 1, Fig. 3C, and Table S1 in Text S1). In contrast, cytosine at position −6 seems to be very important for retrotransposition in *L. lactis* (C-6 scored ∼90% across libraries) irrespective of the presence of relaxase. The chromosomal reads were further mapped to the *L. lactis* IL1403 reference genome [25]. The total number of the unique chromosomal insertion points (the DNA loci invaded by the intron) in the libraries correlated with the presence or absence of relaxase (Fig. 4A). The D-C strain produced only 154 unique intron insertion points, whereas there were 461 and 912 unique insertion sites for D-pRS01 and D-pLtrB, respectively.

We also observed striking differences in the integration frequency distribution among libraries. Whereas, the relative retrotransposition frequency plotted throughout the bacterial chromosome for the D-C strain exhibited a few pronounced peaks, the two relaxase-expressing strains, D-pRS01 and D-pLtrB, showed more uniform frequencies among a larger number of reads. The distribution of the strong peacks in the D-C strain likely reflects replication fork dynamics since the majority of these peacks are located either in close proximity to the origin of replication (*oriC*) or chromosomal replication termination region, *Ter*. Interestingly, the highest frequencies from all the libraries were observed ∼60 Kbp upstream of the putative *Ter*, which is at position ∼1,260,000 in *L. lactis* IL1403 genome [25] (Fig. 4A). It is not clear if there is any advantage for the intron to disrupt the locus upstream to the *Ter* region at a high frequency and at multiple insertion points in *L. lactis* or if the silencing of the region improves the fitness of the bacterial host under the conditions of the experiment. Alternatively, clustering at *Ter* may be related to intron-encoded LtrA protein showing distinctive bipolar localization when expressed in *E. coli* [26], [27].

Although relaxase does not affect intron insertion specificity, more potential sites become available for intron retrotransposition when relaxase is present. We hypothesize that relaxase stimulation is related to primer availability for reverse transcription. Off-target, spurious nicking activity of the relaxase would satisfy the primer requirement by providing the 3′-OH for initiating reverse transcription (Fig. 1C). Although during conjugation, relaxases nick plasmid DNAs in a sequence- and strand-specific manner at *oriT* [16], there are a few examples of conjugative relaxases with loose specificity for *oriT* sequences such that these enzymes can process cryptic origins in bacterial chromosomes [28]. Similar spurious nicking at off-target sites may be the basis for enhanced retrotransposition.

### LtrB relaxase specifically cleaves its cognate *oriT* and also displays off-target activity

**Figure 5:**
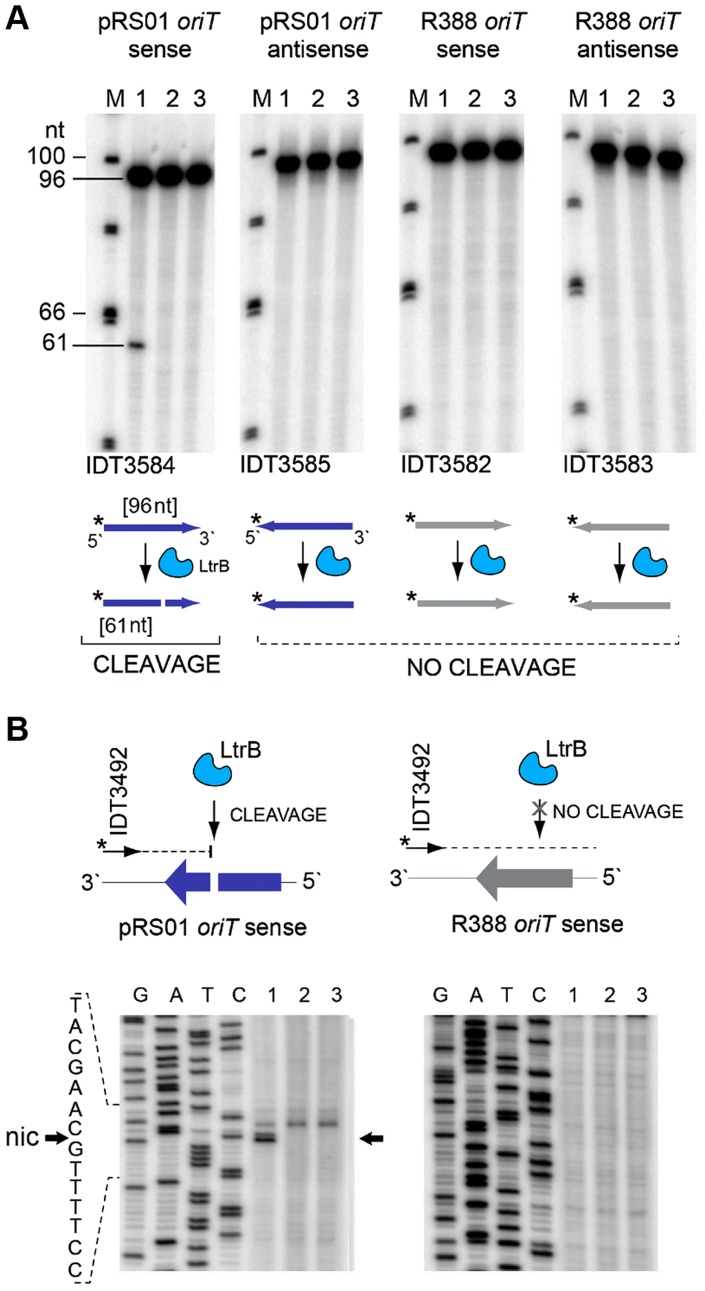
LtrB nicks ssDNA of its cognate *oriT* in sequence- and strand-specific manner. (**A**) LtrB relaxase can cleave ssDNA *oriT* in strand-specific manner. ^32^P-labeled oligonucleotides, containing either pRS01 *oriT* (blue) or R388 *oriT* (grey), were incubated with LtrB-HIS6 (Lane 1), LtrB (Y21A)-HIS6 (Lane 2) or buffer control (Lane 3) in cleavage buffer and products were separated by denaturing PAGE gel alongside φ×174 DNA/Hinf1 marker (Lane M). A line marks the substrate band (96 nt). An arrow shows the cleavage band (61 nt). Cleavage was only observed with wild-type LtrB-HIS6 on pRS01 *ori*T, its native target (Lane 1, left panel). The orientation of arrows, representing oligonucleotides, reflects the strand of *oriT*, either sense or antisense. The asterisk is the site of labeling. (**B**) Mapping of nick site. After incubation with relaxase, primer extension reaction was performed using primer IDT3492, which is specific for the pGEM vector. The cleavage was observed within pRS01 *oriT* and the sequence of *nic* was established (vertical arrow on schematic, horizontal arrows beside autoradiogram). The ssDNA was prepared from ds substrates pON*oriT*1 and pON*oriT*2, and incubated with LtrB-HIS6 (Lane 1), LtrB (Y21A)-HIS6 (Lane 2) or buffer control (Lane 3). These reactions were used in primer extension assays and separated by denaturing PAGE, alongside sequencing ladders. The cleavage band at the nick site is only present with wild-type LtrB-HIS6 (Lane 1) on its native target, pRS01 *ori*T. No cleavage was observed within R388 *oriT* indicating specificity of LtrB relaxase for its cognate *oriT*.

We tested specificity by the ability of pRS01 relaxase to cleave ssDNA of its cognate *oriT* site and an unrelated *oriT* site. The LtrB protein was expressed in *E. coli* and its activity was analyzed (Fig. 5; Figures S3–S4 in Text S1). The *oriT* region of pRS01 was predicted based on sequence similarity with other conjugative plasmids [29] and cloned into pGEM from which ssDNA was prepared. The *oriT* fragment from heterologous plasmid R388 served as a control (Fig. 5). The ssDNA was treated with either wild-type relaxase or the Y21A catalytic mutant relaxase and the reaction mixtures were used for primer extension analysis. A single cleavage band was observed only for ssDNA containing *oriT* from pRS01 treated with relaxase but not with the catalytic Y21A relaxase mutant or when ssDNA carried *oriT* from a heterologous R388 plasmid (Fig. 5A and 5B).

**Figure 6:**
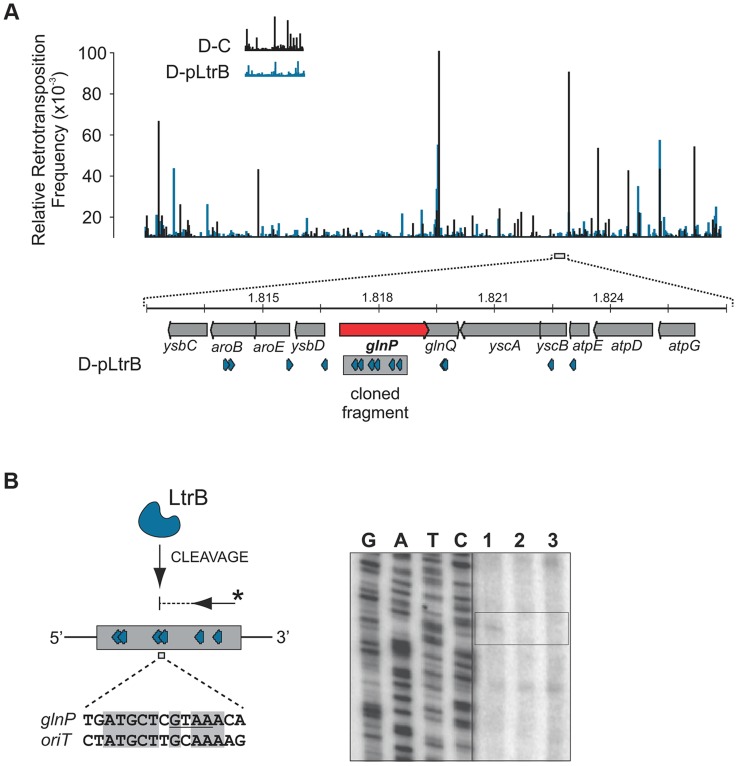
LtrB has off-target activity. (**A**). Mapping of sites and selection of the target sequence. Randomly sampled libraries of the reads for D-C and D-pLtrB were mapped along the *L. lactis* IL1403 chromosome [25] (Tables S2 and S4 in Text S1). The relative retrotransposition frequencies are represented by vertical bars: black, D-C strain without relaxase; blue, pLtrB strains with relaxase. For detection of possible relaxase off-target nicking activity, we chose the *glnP* gene (red arrow), in which six different retrotransposition events were observed in the D-pLtrB strain (blue), but none in control strain D-C. (**B**) LtrB relaxase has weak off-target activity. The ssDNA containing a fragment of the *glnP* gene was incubated with either LtrB-HIS6 (lane 1), catalytic mutant LtrB (Y21A)-HIS6 (lane 2) or left untreated (lane 3). Primer extension reactions (shown schematically) were used to detect cleavage sites. Weak relaxase-specific bands were detected (see also S4 Figure in Text S1). One of the cleavages was mapped between sites for two retrotransposition events detected in the D-pLtrB library. The cleaved sequence is similar to the *oriT* nicking site of pRS01 as shown by pairwise sequence alignment (bottom).

To test if LtrB is able to nick non-cognate chromosomal sites, we selected a 1323-bp genomic fragment containing the glutamine ABC transporter permease gene *glnP* (Fig. 6). This fragment was chosen because of the high density of intron integration events in this region in presence of relaxase. If relaxase is providing the 3′-OH for initiating reverse transcription, the sites for off-target cleavage might be detectable in the presence of relaxase in such regions for which many retrotransposition events occur. Six retrotransposition events with high relative frequencies were mapped in *glnP* and all six were present only in libraries with active LtrB relaxase (D-pRS01 and D-pLtrB). A number of faint primer extension cleavage bands was detected for the *glnP* gene fragment with wild-type relaxase but not with the Y21A relaxase mutant, demonstrating that active relaxase can cleave off-target sites with low efficiency (Fig. 6 and Figure S4 in Text S1). Interestingly, the most efficient nick site in *glnP* has 9 of 15 nucleotides identical to the *oriT* site (Fig. 6B). Thus, the LtrB relaxase not only nicks its cognate *oriT* with high efficiency, but it is also able to cleave chromosomal off-target sites with low efficiency *in vitro*.

## Discussion

**Figure 7:**
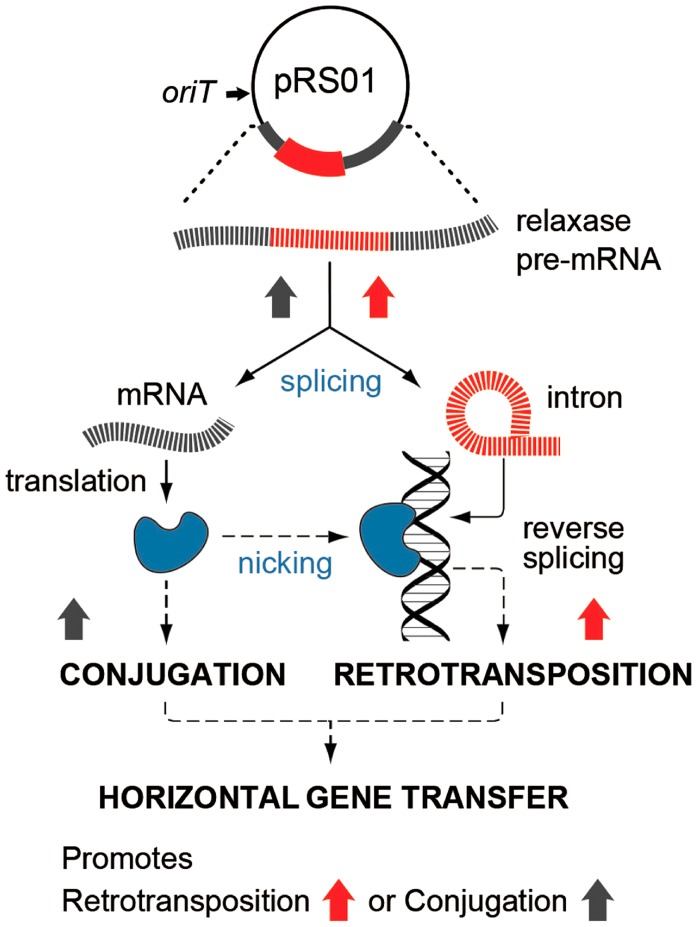
Interaction between pRS01 conjugation and intron retrotransposition. The Ll.LtrB intron (red) resides within the relaxase gene on conjugative plasmid pRS01. RNA splicing enables both formation of the mRNA encoding relaxase and retrotransposition of the intron itself, thereby, stimulating both conjugation and intron mobility (upward-directed arrows). Reciprocally, relaxase (blue symbol) both initiates conjugation and promotes intron dissemination by the dual processes of interbacterial DNA transfer and facilitating intron retrotransposition. Interaction between these two elements thereby promotes gene transfer.

In this study, we show that two such cohabiting elements that move by different DNA- or RNA-based pathways interact to promote gene transfer (Fig. 7). The intron splicing generates LtrB relaxase mRNA and thereby facilitates relaxase expression that initiates conjugation, whereas relaxase promotes retrotransposition, in terms of both the frequency and diversity of events. This stimulation is achieved by the relaxase introducing spurious nicks in recipient DNA, thereby providing 3′-OH ends that prime reverse transcription for retrotransposition. Thus, the promiscuous activity of this nickase is the driver that links conjugal mating and retrotransposition, which is elevated in both donor cells (this work) and in the recipient [19], [20].

In our model for relaxase-mediated stimulation of retrotransposition, we propose that when there is sufficient sequence similarity to the homing site to allow intron RNA and chromosomal DNA interaction upstream of the nick site, the intron will reverse splice into the degenerate homing site, while the 3′-OH of the nicked bottom strand provides a primer for reverse transcription (Fig. 1C). cDNA synthesis and repair will then proceed using standard retromobility reactions (*cf.* Figs. 1C with 1A and 1B). How the relaxase might be removed from the nick site is not known. Although reversal of the relaxase reaction might be invoked in ligating the nick to complete retrotransposition and remove itself from the DNA, our unpublished experiments suggest that this is not generally the case.

Transposition stimulated by DNA damage has been shown for a number of bacterial transposons [30], [31]; some prophages are also induced following the DNA damage [32]. Nicks introduced by relaxase, if they remain unligated, could be converted into DNA double-strand breaks during a replication fork collapse [33], [34]. It will therefore be interesting to determine if relaxases stimulate other transposons that utilize various DNA intermediates such as DSBs or 3′ ends to mobilize, e.g. Tn7 transposon in *E. coli* [30].

Transcription and translation of the *ltrB* gene is growth phase-dependent [35] suggesting that retrotransposition may be coupled to cellular physiology. Transcription of the relaxase operon (Fig. 2A) and the *ltrB* gene in particular is essential for Ll.LtrB retrotransposition since the intron RNA is the template for the synthesis of the first cDNA strand in chromosomal intron integration (Fig. 1). At the same time, efficient and precise splicing of Ll.LtrB is crucial for pRS01 conjugative transfer due the requirement for functional relaxase protein for the initiation of conjugation (Fig. 7). Splicing of the intron depends on the IEP LtrA, which is expressed at low levels under control of an internal promoter [35], in addition to being autoregulated [36]. This independent regulation of the expression of the IEP LtrA and LtrB relaxase, which control splicing/reverse splicing and conjugation, respectively, provide the opportunity for the intron to act as a sensor to control conjugation, and reciprocally, for the relaxase to promote retrotransposition when conjugation is stimulated and conditions are rife for horizontal gene transfer. This interplay is likely important in stressful environments, when retrotransposition of the group II intron is stimulated, as for example during starvation [37]. Likewise, conjugative elements in different bacterial systems respond to environmental and physiological cues including DNA damage [38], [39].

Not only does LtrB relaxase provide the first example of a stimulatory factor of RNA-based group II intron retrotransposition in a native host, but also the promiscuous activity of this nickase promotes the link between conjugation and retrotransposition. This scenario is consistent with the unusually high retromobility frequency of the Ll.LtrB intron immediately after conjugation in some transconjugant isolates [19]. Thereby, this link between the mobilization apparatus of a conjugative element and a group II intron provides an example of a stimulatory force for DNA spread among bacterial populations and the infectious transfer of mobile genetic elements which could lead to rapid evolution of bacterial genomes.

## Materials and Methods

### Bacterial strains and growth conditions

All strains and plasmids used in this study are listed in Table 1. *E. coli* DH5α, TOP10 and TOP10F′ strains were grown in Luria Broth (LB) medium at 37°C with aeration. *L. lactis* strains were grown in M17 with 0.5% glucose (w/v) (GM17 media) at 30°C without aeration. Where appropriate, the media contained erythromycin (150 µg/ml for *E. coli* and 10 µg/ml for *L. lactis*), spectinomycin (50 µg/ml for *E. coli* and 300 µg/ml for *L. lactis*), or chloramphenicol (25 µg/ml for *E. coli* and 10 µg/ml *L. lactis*).

Electroporation of both *E. coli* and *L. lactis* was performed with a Gene Pulser apparatus (BioRad). *E. coli* transformants were recovered in SOC media (0.5% yeast extract, 2% tryptone, 10 mM NaCI, 2.5 mM KCI, 10 mM MgCI_2_, 10 mM MgS0_4_ and 20 mM glucose) for 1 h at 37°C with aeration. *L. lactis* transformants were recovered in GM17 medium with 0.5 M sucrose for 3 h at 30°C without aeration.

### Plasmid methodology, enzymes and oligonucleotides

Plasmid DNA was isolated and purified using a QIAprep Spin Miniprep Kit (Qiagen). The digests and PCR fragments were visualized by electrophoresis in 0.7% (w/v) agarose gels and stained with ethidium bromide. DNA fragments were purified from agarose gels with QIAquick Gel Extraction Kit (Qiagen). T4 DNA ligase from Promega was used for ligation. The list of oligonucleotides used in this study is in Table 2. The sequences of all fragments generated by PCR were verified.

**Table 2:** Oligonucleotides used in present study.

Oligo ID	Sequence (5′→3′)	Application
W1746	TCAACTGCAGAGAGAGAAAAATAATGCGGTGC	Ll.LtrB:RIG fragment amplification
W1747	AGCCACTAGTTTAGGAATGACTTTCCAGTC	Ll.LtrB:RIG fragment amplification
IDT1544	CTAGACTAGTTAACTCAGCAGCTCTCTGAAATTC	intron-containing *ltrB* gene amplification
IDT1545	ACATGCATGCTTATAGTATTTTTCCTTTATTTTC	intron-containing *ltrB* gene amplification
IDT2014	TCTTTTTCCTTTTATTTCAAATCCAGA	Y21F mutagenesis
IDT2015	TTTGAAATAAAAGGAAAAAGATTTGTG	Y21F mutagenesis
IDT2016	TCTTTTTCCTGCTATTTCAAATCCAGA	Y21A mutagenesis
IDT2017	TTTGAAATAGCAGGAAAAAGATTTGTG	Y21A mutagenesis
IDT2018	CTCATCCAACGATTTTCTCCTGA	S117R mutagenesis
IDT2019	AGGAGAAAATCGTTGGATGAGGT	S117R mutagenesis
IDT2020	CTCATCCAAGCATTTTCTCCTGA	S117A mutagenesis
IDT2021	AGGAGAAAATGCTTGGATGAGGT	S117A mutagenesis
IDT2022	TGAACACATCCGTAACCATATCATT	H159R mutagenesis
IDT2023	GTTACGGATGTGTTCACGATCG	H159R mutagenesis
IDT2024	TGAACACATCGCTAACCATATCATT	H159A mutagenesis
IDT2025	GTTAGCGATGTGTTCACGATCG	H159A mutagenesis
IDT2026	ACATCCATAACCGTATCATTTTTAATT	H161R mutagenesis
IDT2027	AAATGATACGGTTATGGATGTGTTCA	H161R mutagenesis
IDT2028	ACATCCATAACGCTATCATTTTTAATT	H161A mutagenesis
IDT2029	AAATGATAGCGTTATGGATGTGTTCA	H161A mutagenesis
IDT3381	TGAGAGCTCAAGGAGGTTAATCTAATGCATCATCATCATCACCACGTTTACACAAAACACATTATTGTTC	*ltrB* gene amplification for cloning in pBAD33
IDT3277	ACAGCATGCTTATAGTATTTTTCCTTTATTTTCTTCAAG	*ltrB* gene amplification for cloning in pBAD33
IDT3509	TCTCACAAATCTTTTTCCTGCTATTTCAAATCCAGACAAGAC	Y21A direct mutagenesis
IDT3510	GTCTTGTCTGGATTTGAAATAGCAGGAAAAAGATTTGTGAGAT	Y21A direct mutagenesis
IDT3584	CGATCGACTAGTGCTATCCATTTCTTAAATTCCGTAAGATGCTATCATCTTACTATGCTTGCAAAAGGTCAAGGAAGTACATAGCATGCCAGTAGA	pRS01 *oriT*; activity assay; *nic* determination
IDT3585	CTACTGGCATGCTATGTACTTCCTTGACCTTTTGCAAGCATAGTAAGATGATAGCATCTTACGGAATTTAAGAAATGGATAGCACTAGTCGATCGA	pRS01 *oriT*; activity assay; *nic* determination
IDT3582	CGATCGACTAGTGCTAGAATGACTTACGCGCACCGAAAGGTCGTATTGTCTATAGCCAGGCGAATTCGTAGTGTTACTACATAGCATGCCAGTAGA	R388 *oriT*; activity assay
IDT3583	CTACTGGCATGCTATGTAGTAACACTACGAATTCGCCTGGCTATAGACAATACGACCTTTCGGTGCGCGTAAGTCATTCTAGCACTAGTCGATCGA	R388 *oriT*; activity assay
IDT3492	GGGCCCGACGTCGCA	LtrB *nic* determination; cleavage mapping
IDT3751	CTTTCTGATTACAGTGATCTTAAAGG	*glnP* locus cloning
IDT3752	GATAAAGATTGTTAATACTAAGAGCGG	*glnP* locus cloning; cleavage mapping pON*LLglnP*-R
IDT3822	GAAGCAGTTCCGTTTTTAGCACC	*glnP* locus cleavage mapping pON*LLglnP*-R
Illumina sequencing		
P7A	/5Phos/GATCGGAAGATCGTATGCCGTCTTCTGCTTG	Illumina adapter
P5A	ACACTCTTTCCCTACACGACGCTCTTCCGATC∧T	Illumina adapter
P7	CAAGCAGAAGACGGCATACGAGCTCTTCCGATCT	P7-specific primer
P-SJ	CAGCTCTCTTGGGTCTTTAAATGGAGTGTCTTCTTCCC	splice-junction specific primer
P5i1	AATGATACGGCGACCACCGAGATCTACACTCTTTCCCTACACGACGCTCTTCCGATCTatcacgNNNNNNCAAGGCGGTACCTCCCTACTTCAC *	D-C library amplification
P5i2	AATGATACGGCGACCACCGAGATCTACACTCTTTCCCTACACGACGCTCTTCCGATCTttaggcNNNNNNCAAGGCGGTACCTCCCTACTTCAC *	D-pRS01 library amplification
P5i3	AATGATACGGCGACCACCGAGATCTACACTCTTTCCCTACACGACGCTCTTCCGATCTcagatcNNNNNNCAAGGCGGTACCTCCCTACTTCAC *	D-pLtrB library amplification

*- sequence of the library-specific barcode is underlined.

### Construction of plasmids and strains

Construction of the intron donor pLNRK-RIG was accomplished by inserting a fragment of Ll.LtrB:RIG [11] into *Pst*I and *Spe*I sites of *L. lactis*/*E. coli* shuttle vector, pLNRK, a pLE1-based plasmid [40], containing a nisin promoter and *nisR* and *nisK* genes cloned into the ApaLI site. The Ll.LtrB:RIG fragment was generated by PCR with primer pair W1746/W1747 from plasmid pLERIG [11]. The strain D-C (D = Donor plasmid, Control) is *L. lactis* IL1403 transformed with pLNRK-RIG and selected for Cam^R^.

To create the D-pRS01 strain, a plasmid pRS01::pTRK28 (Erm^R^) from *L. lactis* DM2036 [40] was transferred to *L. lactis* IL1403 by conjugation using plasmid pDL278 (Spc^R^) as a marker for antibiotic resistance in the recipient and selecting for Erm^R^ and Spc^R^. The pDL278 marker plasmid was cured by growing in the presence of 2 µM ascorbic acid and selecting only for pRS01::pTRK28 (Erm^R^). The resulting strain (Erm^R^, Spc^S^ and Cam^S^) was transformed with pLNRK-RIG and clones were selected for Cam^R^ giving rise to D-pRS01 strain.

D-pRS01Δ*ltrB* strain was created in two steps. First, *L. lactis* IL1403 containing pRS01::pTRK28 was transformed with plasmid pBlueScriptΔ*ltrB*::*tet* (Tet^R^), in which the *Hin*dIII fragment of *ltrB* was replaced by a *tet* marker, and which does not replicate in *L. lactis*. Recombinants were selected for Erm^R^ and Tet^R^, and double crossover mutants that resulted in pRS01Δ*ltrB*::*tet* (Erm^R^, Tet^R^) were screened for the absence of the pBlueScript vector and *ltrB* gene and the presence of the *tet* ^R^ gene. Recombinants were confirmed by sequencing and the strain carrying pRS01Δ*ltrB*::*tet* was transformed with pLNRK-RIG followed by selection for Cam^R^.

The D-pLtrB strain was made by transformation of the D-C strain with complementation plasmid pLtrB:Ll.LtrB carrying the *ltrB* relaxase gene under a nisin-inducible promoter. The pLtrB:Ll.LtrB was constructed by amplifying the intron-containing *ltrB* gene from pRS01::pTRK28 as a template by PCR using primer pair IDT1544/IDT1545 and inserting the resulting fragment into *Spe*I and *Sph*I sites of the expression vector pCJK21 [41]. The pLtrB:Ll.LtrB plasmid contains an unmarked native copy of the Ll.LtrB intron so as to eliminate the Ll.LtrB group II intron homing site and ensure measurement of retrotransposition and not retrohoming into intron-less *ltrB*.

The relaxase catalytic mutant strains were prepared by transformation of the D-C strain with a plasmid carrying a mutated *ltrB* relaxase gene. The mutations were introduced using a two-step SOEing (Synthesis by Overlap Extension) PCR. First, the fragments of *ltrB* sequence were amplified, introducing the desired mutations using mismatched primer sequences. Second, the corresponding fragments were mixed and used as the templates to amplify the full-length mutated variants of the *ltrB* gene. The resulting PCR products were subcloned into TOPO vector using Zero Blunt TOPO PCR Cloning Kit (Life Technologies) and clones of interest were detected by colony PCR to confirm the presence of the insert using universal M13 primers. Plasmid DNA was digested with *Sph*I and *Spe*I for 2 h at 37°C and the insert was ligated into pCJK21 backbone into *Sph*I and *Spe*I sites. After propagation in *E. coli* DH5α and confirmation of the insertion with PCR, plasmid DNA was isolated and used for subsequent *L. lactis* transformations.

### Retrotransposition assay

Retrotransposition assays in *L. lactis* were performed in specific strains with the intron donor plasmid as previously described [11]. Overnight cultures were grown in GM17 medium supplemented with appropriate antibiotics at 30°C without aeration. Cultures were diluted 1∶100 in fresh GM17, grown to OD_600_ of 0.2, and intron expression was induced by addition of nisin at 10 ng/ml. Cultures were grown for an additional 3 h, plated on GM17 medium with and without kanamycin (160 µg/ml) and incubated at 30°C for 2 days. The number of colony-forming units on kanamycin plates was used to estimate retrotransposition frequencies relative to total number of colonies on plates lacking kanamycin.

### Preparation of Illumina libraries for RIG-Seq

Bacterial colonies growing on the selective plates (kanamycin 160 µg/ml) from three replicates of the retrotransposition assay were scooped from the plate surface, washed in phosphate buffered saline (PBS; 137 mM NaCl, 2.7 mM KCl, 10 mM Na_2_HPO_4_, 1.8 mM KHPO_4_, pH 7.4) and stored at −70°C. DNA was isolated using DNeasy Blood & Tissue Kit (Qiagen) following the protocol designed for purification of total DNA from Gram-positive bacteria. The quality and quantity of the resulting DNA was assessed using electrophoresis in a 0.7% (w/v) agarose gel stained with ethidium bromide and NanoDrop (Thermo Scientific), respectively. The total DNA (2 µg in 100 µl final volume of TE buffer) was fragmented using a Bioruptor Standard (Diagenode) with the following parameters: 30 min of 30 sec on/off cycles at 4°C. The sheared DNA was visualized on a 0.7% (w/v) agarose gel stained with ethidium bromide and fragments in the range of 200 bp–800 bp were isolated from the gel with a QIAquick Gel Extraction Kit (Qiagen).

Preparation of genomic DNA from *kan^R^* colonies was followed by ligation of standard adapters, P5A and P7A, containing Illumina flow-cell binding sites. End repair of fragmented DNA was performed using a NEBNext End Repair Module. The NEBNext dA-Tailing Module and NEBNext Quick Ligation Module were utilized for dA-tailing of the end-repaired DNA and adapter ligation of dA-tailed DNA. The Phusion High-Fidelity PCR Master Mix was used for PCR enrichment of adapter-ligated DNA. Purification of the fragments when necessary was performed with QIAquick PCR Purification Kit (Qiagen).

Specific primer P-SJ and the universal primer P7 enable amplification of the fragment containing the 5′ end of the *kan^R^* gene, the 3′ end of the Ll.LtrB intron, and the flanking genomic sequence of interest. At this step, the amplicons lack the necessary P5 adapter sequence which is reintroduced as ‘chimeric’ primer P5i (Fig. 3A). The region at the 3′ end of P5i is complementary to the short 3′ end fragment of the Ll.LtrB intron, joined by a six-base index sequence and a stretch of six random nucleotides to the 5′ adapter sequence (Fig. 3A). The product of the second PCR has P5A and P7A flow cell binding sites as well as the index sequence, which is specific for each constructed sequence library, allowing multiplex sequencing. Sequence libraries were constructed after selection of Kan^R^ retrotransposition events for the different strains, each bar-coded with a unique index sequence (Fig. 3A and Table 2) and sequencing was performed by the Core Facility at SUNY Buffalo (Buffalo, NY). The sequences containing the 3′ flanking genomic sequence, generated on an Illumina HiSeq 2000, were analyzed, to generate unambiguous profiles for each of the libraries.

### Illumina read analysis and mapping

The adapter and intron sequences were removed from reads using custom BioPhyton script (scripts available on request). Not only were adapter and barcode sequences removed, but also the sequence corresponding to the 3′ end of the intron had to be cut out, leaving only the flanking sequence of interest prior to mapping. The script allows precise detection of the intron fragment within the generated reads followed by the trimming of the intron and the upstream sequence leaving the flanking fragment. The 5 bp at the 3**′** end of the reads appeared to be of low quality and were removed as well. The resulting 15–16 bp reads were analyzed.

The frequencies and distribution of the reads along the *L. lactis* IL1403 chromosome could not be compared directly even after digital normalization. This was because Illumina libraries derived from different cultures not only consisted of a different number of chromosomal retrotransposition events (Fig. 3B), but also exhibit striking differences in distribution of these events along the chromosome for different libraries. To overcome this obstacle, we first weeded out all non-chromosomal reads from the original libraries (including plasmid events and unmapped reads). Next, we performed random sampling of the reads for each library, generating new libraries containing only chromosomal reads. BioPython script (fastq_reservoir_sampling.py) was used for random sampling of the reads from each library for further comparative analysis. The 1,000 randomized subsets of the same size (250,000 reads) were automatically generated for each of the libraries. The number of unique insertion points was estimated for each subset and the average numbers are provided in Fig. 4A.

The analysis and mapping of the Illumina-generated reads was performed using the Galaxy server (https://usegalaxy.org/) [42]. Bowtie software was used for mapping of the reads under custom settings [43]. The settings were as follows: ‘Maximum number of mismatches permitted in the seed’ was set as ‘0’ (parameter -n), and ‘Whether or not to try as hard as possible to find valid alignments when they exist’ was set to ‘Try hard’ (parameter -y). Additionally, the setting was to report the ‘best’ singleton alignments in terms of stratum (the number of mismatches) and in terms of the quality values at the mismatched positions (parameter –best). FASTQ Groomer [44], SAMtools [45], deepTools [46], and BEDTools [47] were also used for analysis among other bioinformatic tools implemented in the Galaxy server (https://usegalaxy.org/) [42].

### Intron insertion site analysis

DNA sequences for 5′ and 3′ flanks of the intron insertion were aligned using ClustalW algorithm incorporated into Unipro UGENE software [48] and compared to the sequence of the Ll.LtrB homing site (Tables S1–S4 in Text S1). Since we used the whole set of sequences irrespective of the frequencies of the retrotransposition events for each particular insertion point, the scores and nucleotide distribution in each position reflect the whole spectrum of the sequences which might become a potential target for the intron. The alignments were optimized based on the relative frequency of each retrotransposition event to better reflect the overall preferences for the insertion site nucleotide composition in different libraries (Table S1 in Text S1). Sequence logos were drawn for each of the libraries based on the resulting alignments using WebLogo [49] (Fig. 3C). The cut-off for relative frequency was equal to 5.0×10^−3^, which means that only retrotransposition events with higher frequencies were used in the present analysis. The nucleotides relative to the insertion point are numbered on the top of each Table (Table S1): “−” indicates the location upstream relative to the putative insertion site, “+” indicates downstream. The grid profiles and consensus sequences (Cons.) were built using Unipro UGENE software [48] and its feature ‘Statistics’.

### Overexpression and purification of relaxase

The *E. coli* TOP10-pBAD33 host-vector expression system [50] was used to clone and overexpress the *ltrB* gene in plasmids pLtrB-HIS6 and pLtrB(Y21A)-HIS6. To construct the expression plasmid pLtrB-HIS6, the full-length intronless *ltrB* gene was amplified from pCY20 plasmid as template [29] using primer pair IDT3381/IDT3277. The resulting PCR fragment was digested with *Spe*I and *Sph*I at 37°C for 2 h. The fragment was ligated into pBAD33 [50], which had been digested with *Sph*I and *Spe*I overnight. The expression plasmid pLtrB(Y21A)-HIS6 was produced by site-directed mutagenesis from pLtrB-HIS6 with the GeneArt Site-Directed Mutagenesis System (Invitrogen, Life Technologies) using the primers IDT3509 and IDT3510 containing a mutation in the corresponding codon to convert Tyr to Ala at position 21.

*E. coli* TOP10 carrying LtrB-HIS6 or LtrB(Y21A)-HIS6 were grown in LB medium supplied with chloramphenicol at 30°C to O.D. ∼0.6. Induction was with 0.4% arabinose (w/v) for 4 h. Proteins were separated by 10% SDS-PAGE [51], and visualized by staining with Coomasie brilliant blue. For preparative purposes, cells from 700 ml induced culture were collected by centrifugation and stored at −70°C. Pellets were thawed on ice, suspended in 40 ml of Lysis Buffer (50 mM Tris-HCl, pH 8.0, 2 mM EDTA, 10 mM imidazole, 10% [v/v] glycerol) and incubated on ice for 30 min. Next, cells were lysed by sonication, and debris was removed by centrifugation. The HIS6-tagged proteins were purified using metal chelating chromatography under native conditions at 4°C. Lysis Buffer was used for equilibration of the column. Wash Buffer (50 mM NaH_2_PO_4_, 300 mM NaCl, 50 mM imidazole, pH 8.0) and Elution Buffer (50 mM NaH_2_PO_4_, 300 mM NaCl, 200 mM imidazole, pH 8.0) were used in further purification with the HisTrap HP (GE Healthcare) columns containing Ni Sepharose High Performance following the manufacturer instructions.

### Oligonucleotide cleavage assay

The IDT3584 and IDT3585 oligonucleotides 96-nt in length containing pRS01 *oriT* in sense and antisense orientation [29], and the IDT3582 and IDT3583 oligonucleotides 96-nt in length containing R388 *oriT* in sense and antisense orientation were used for activity assays [52]. Oligonucleotides were labeled with 1–2 U of T4 polynucleotide kinase (NEB) and 25 µM γ-[^32^P]-ATP (5×10^6^ cpm/µmol; Amersham) for 1 h at 37°C followed by purification with illustra MicroSpin G-50 columns (GE Healthcare). The reaction mixture (20 µl) contained relaxase reaction buffer (50 mM Tris-HCl pH 7.9, 100 mM NaCl, 10 mM MgCl_2_, 1 mM dithiothreitol), 100 µg/ml bovine serum albumin (BSA), 20 ng LtrB-HIS6 or LtrB(Y21A)-HIS6 protein, and 3 pmol 5′-labeled [^32^P]-ATP-oligonucleotide primer. After 1 h incubation at 37°C, reaction products were analyzed by denaturing polyacrylamide gel electrophoresis (PAGE) (8% [*w/v*] 29∶1 acrylamide/Bis-acrylamide, 7 M urea, 1× Tris/Borate/EDTA buffer) followed by imaging using phosphor screen in a Typhoon imager (GE Healthcare).

### Cleavage of ssDNA and mapping of the cleavage sites

The ssDNA was prepared from *E. coli* TOP10F′ cells transformed with plasmids: pON*oriT1*, pON*oriT2*, or pON*LLglnP*-R using M13KO7 helper phage (NEB) following the manufacturer protocol. Plasmids pON*oriT1* and pON*oriT2* were constructed by AT-cloning of the *oriT* 95-bp fragments from pRS01 [29] and R388 plasmids [52] into pGEM-T vector (Promega) in the direct orientation relative to the phage f1 origin (f1 *ori*) carried by pGEM following the manufacturer's recommendation. The following oligonucleotides were used: IDT3584 and IDT3585 which contain *oriT* from pRS01 [29]; IDT3582 and IDT3583 which contain *oriT* from R388 [52]. Plasmid pON*LLglnP*-R was constructed by AT-cloning of the fragment of *glnP* locus, 1323 bp in length, amplified from *L. lactis* IL1403 genomic DNA using the following pair of primers: IDT3751 and IDT3752. Plasmid pON*LLglnP*-R carried the fragment in direct orientation relative to the phage f1 *ori*.

The ssDNA (500 ng) was incubated for 1 h at 37°C with either LtrB-HIS6 or LtrB(Y21A)-HIS6 protein in 50 µl relaxase reaction buffer described above. Treated DNA was used directly in the primer extension reaction. Each extension reaction contained ssDNA (50 ng), reaction buffer (50 mM KCl, 20 mM Tris-HCl pH 8.8, 10 mM MgCl_2_, and 100 µg/ml BSA), 200 µM dNTP, 3 pmol 5′-labeled [^32^P]-ATP-oligonucleotide primer, and 1–2 U of Bst DNA polymerase Large Fragment (NEB). The reaction mix was incubated at 65°C for 15 min. The reaction products were analyzed by denaturing PAGE as described above, alongside sequencing ladders (USB 785001KT). The primer IDT3492 is specific for pGEM and was used for all ssDNAs; additionally, *glnP*-specific primers IDT3752, and IDT3822 were used.

## Supporting Information

Text S1 Supporting Information. **Figure S1.** Effect of relaxase mutations on retrotransposition. **Figure S2.** Donor plasmid copy number and mapping of retrotransposition events. **Figure S3.** Purification of LtrB relaxase and its activity with dsDNA targets. **Figure S4.** Mapping of off-target relaxase cleavage. **Table S1**. Grid profiles based on the multiple sequence alignments of the chromosomal insertion sites. **Table S2**. Multiple alignment of the chromosomal insertion sites of Ll.LtrB intron in the absence of the relaxase (D-C strain). **Table S3**. Multiple alignment of the chromosomal insertion sites of Ll.LtrB intron in the presence of the relaxase (D-pRS01 strain). **Table S4**. Multiple alignment of the chromosomal insertion sites of Ll.LtrB intron in the presence of the relaxase (D-pLtrB strain).
(PDF)
