# Supplementary material for: Interaction between Conjugative and Retrotransposable Elements in Horizontal Gene Transfer
Source: PLoS Genet. 2014 Dec 4;10(12):e1004853. doi: 10.1371/journal.pgen.1004853 (PMC4256276; doi:10.1371/journal.pgen.1004853)
Supplement: Text S1 — Supporting Information. Figure S1. Effect of relaxase mutations on retrotransposition. Figure S2. Donor plasmid copy number and mapping of retrotransposition events. Figure S3. Purification of LtrB relaxase and its activity with dsDNA targets. Figure S4. Mapping of off-target relaxase cleavage. Table S1. Grid profiles based on the multiple sequence alignments of the chromosomal insertion sites. Table S2. Multiple alignment of the chromosomal insertion sites of Ll.LtrB intron in the absence of the relaxase (D-C strain). Table S3. Multiple alignment of the chromosomal insertion sites of Ll.LtrB intron in the presence of the relaxase (D-pRS01 strain). Table S4. Multiple alignment of the chromosomal insertion sites of Ll.LtrB intron in the presence of the relaxase (D-pLtrB strain). (PDF) [file pgen.1004853.s001.pdf]

Supplementary Materials:

Supplementary Figure Legends

Figure S1. Effect of relaxase mutations on retrotransposition.

(**A**) Results of retrotransposition assays using mutants of D-pLtrB. The mutation in the catalytic tyrosine (likely Y21 in LtrB; GenBank: AAB06502), results in a 4-fold reduction in RTP frequency with alanine (strain Y21A) or phenylalanine (Y21F) substitutions in comparison with wild-type LtrB relaxase (Figure 2C). Other mutants have a more subtle phenotype.

(**B**) Multiple sequence alignment of relaxases. Amino acid sequences of the three most conserved and catalytically important motifs (Motifs I-III) are shown. Amino acid sequences of relaxases were obtained from NCBI GenBank Protein Database (http://www.ncbi.nlm.nih.gov/protein); the accession number for each sequence is indicated in square brackets. The conserved amino acids are shown with different background shading within alignment; the invariable amino acids are white on black background. The sequence logo for each motif shown at the top was built using WebLogo [49]. The putative catalytic tyrosine is highlighted in blue, it corresponds to Y21 in LtrB relaxase from pRS01 conjugative plasmid (GenBank:U50902).

Figure S2. Donor plasmid copy number and mapping of retrotransposition events.

(**A**) Copy number of donor plasmid.We estimated the relative copy number of donor plasmid pLNRK-RIG among strains after using standard protocols for DNA-DNA hybridization of DNA isolated as described in Materials and Methods. The 5′-labeled [^{32}P]-ATP-oligonucleotide primer (MB14td 5'-TGTCACCATGCAGAGCAGAC-3') complementary to the group I intron in RIG of pLNRK-RIG was used to detect the donor plasmid.

(**B**) Retrotransposition events into pLNRK-RIG. We performed mapping of the reads using pLNRK-RIG as a reference sequence with Bowtie software under custom settings [43] (see Materials and Methods). The most prevalent sequence fragment generated from the D-C library appeared to be the 3' region flanking the Ll.LtrB intron derived from the pLNRK-RIG vector itself (position 5782; shown by grey arrow) indicating the high level of the background amplification. The distribution of the RTP events in the pLNRK-RIG plasmid varied between libraries but supports the previous observation concerning cold-spots for intron invasion in the

essential replication region of *repD, repE*, and pAMβ *ori* [22]. Hot-spots for RTP events were scattered throughout the intron sequence but not in the RIG cassette (*kan*^{R} and group I intron).

Figure S3. Purification of LtrB relaxase and its activity with dsDNA targets.

(**A**) LtrB-HIS6 protein purification (see Materials and Methods). Abbreviations: M, marker; incl. bodies, inclusion bodies; FT, flow through; W, wash.

(**B**) DNA target fragment. The pRS01 *oriT* (96 bp fragment; dark blue) from the conjugative element was cloned into pGEM-T vector (Promega) to create pON*oriT1*. High quality primarily supercoiled plasmid was isolated from fresh *E. coli* DH5α overnight culture and was used for relaxase activity assays.

(**C**) LtrB relaxase from pRS01 cannot nick its cognate *oriT* in dsDNA. Plasmid pON*oriT1* (dsDNA) was incubated with 20 ng of either LtrB-HIS6 (Lane 1) or LtrB(Y21A)-HIS6 (Lane 2) or left untreated (Lane 3) for 1 h at 37°C in reaction (50 µl) containing relaxase reaction buffer (50 mM Tris-HCl pH 7.9, 100 mM NaCl, 10 mM $MgCl_2$, 1mM dithiothreitol) and 100 µg/ml bovine serum albumin (BSA). Half of the reaction mixture was treated with 0.5 µl of proteinase K (NEB) for an additional 30 min at 37°C. Untreated and treated samples were analyzed on a 0.7% agarose gel after staining with ethidium bromide. Supercoiled plasmid, nicked circles and plasmid-relaxase complexes are shown.

Figure S4. Mapping of off-target relaxase cleavage.

(**A**) Cloning of the *glnP* locus for ssDNA preparation. The genomic *glnP* locus was chosen for further tests of LtrB relaxase activity on non-cognate substrate. Plasmid pON*LLglnP*-R was constructed for ssDNA preparation. pON*LLglnP*-R carried the fragment in direct orientation relative to the phage f1 *ori*.

(**B**) Schematic representation of the cloned fragment of *glnP* gene and mapping strategy. The positions of cloned fragment (grey box) and six intron insertion sites (blue arrows) are shown. The positions of primers used in the primer extension reactions are shown on the top by black arrows with oligonucleotide number. Cleavages (designated as 1 and 2) were observed within the cloned fragment and the location of the cleavage sites was established (vertical arrows).

(**C**) LtrB relaxase has off-target activity. Primer extension products were analyzed on denaturing PAGE gels. Some subtle additional bands were observed in several reactions (black spheres, and

boxed on gel) with LtrB-HIS6-treated samples (Lane 1), but not LtrB(Y21A)-HIS6-treated DNA (Lane 2) or the negative control (Lane 3). Results are shown for pON*LLglnP*-R. The position of the cleavage is shown schematically at the top of each gel, and the primer used in the reaction is indicated at the bottom of the gel.

Supplementary Figures

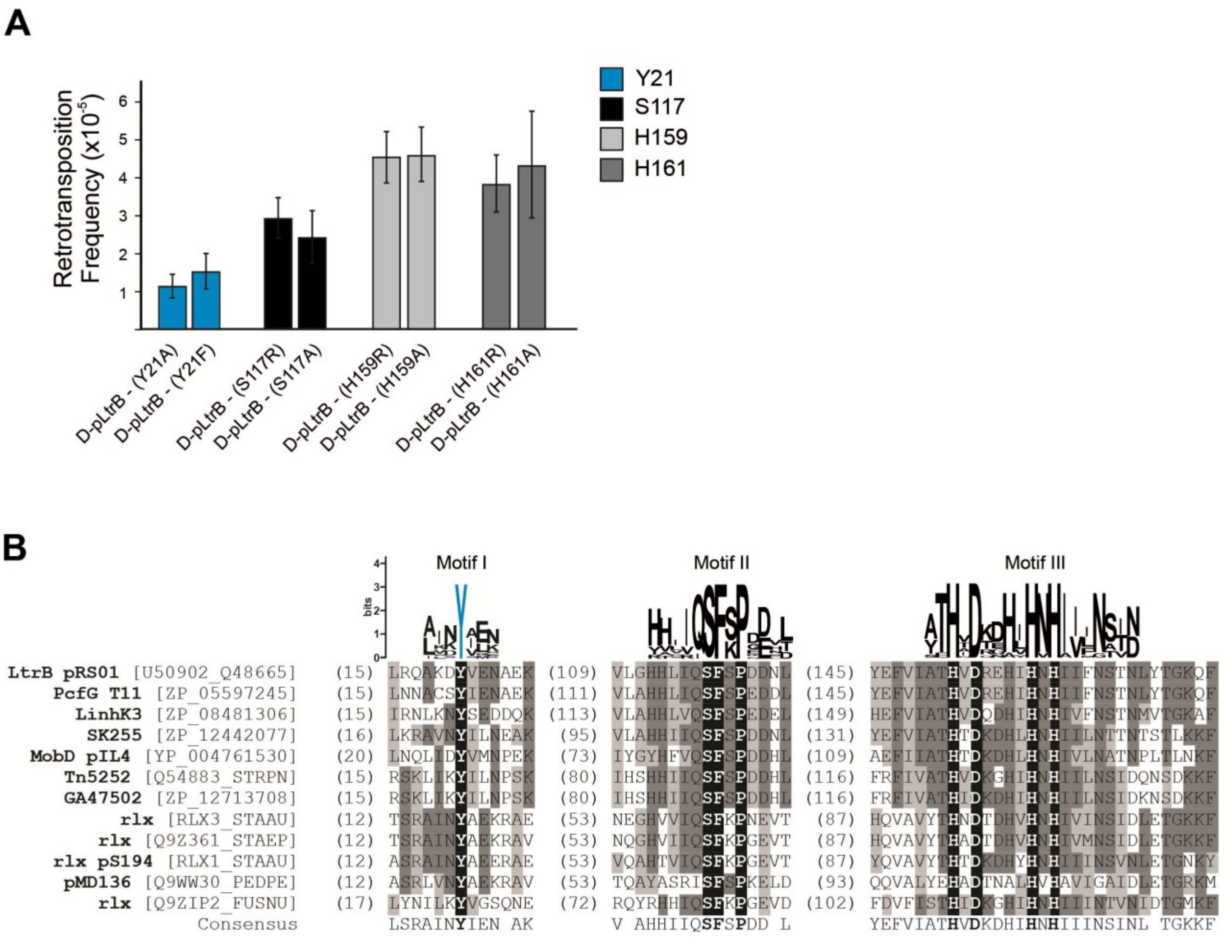

Figure S1. Effect of relaxase mutations on retrotransposition

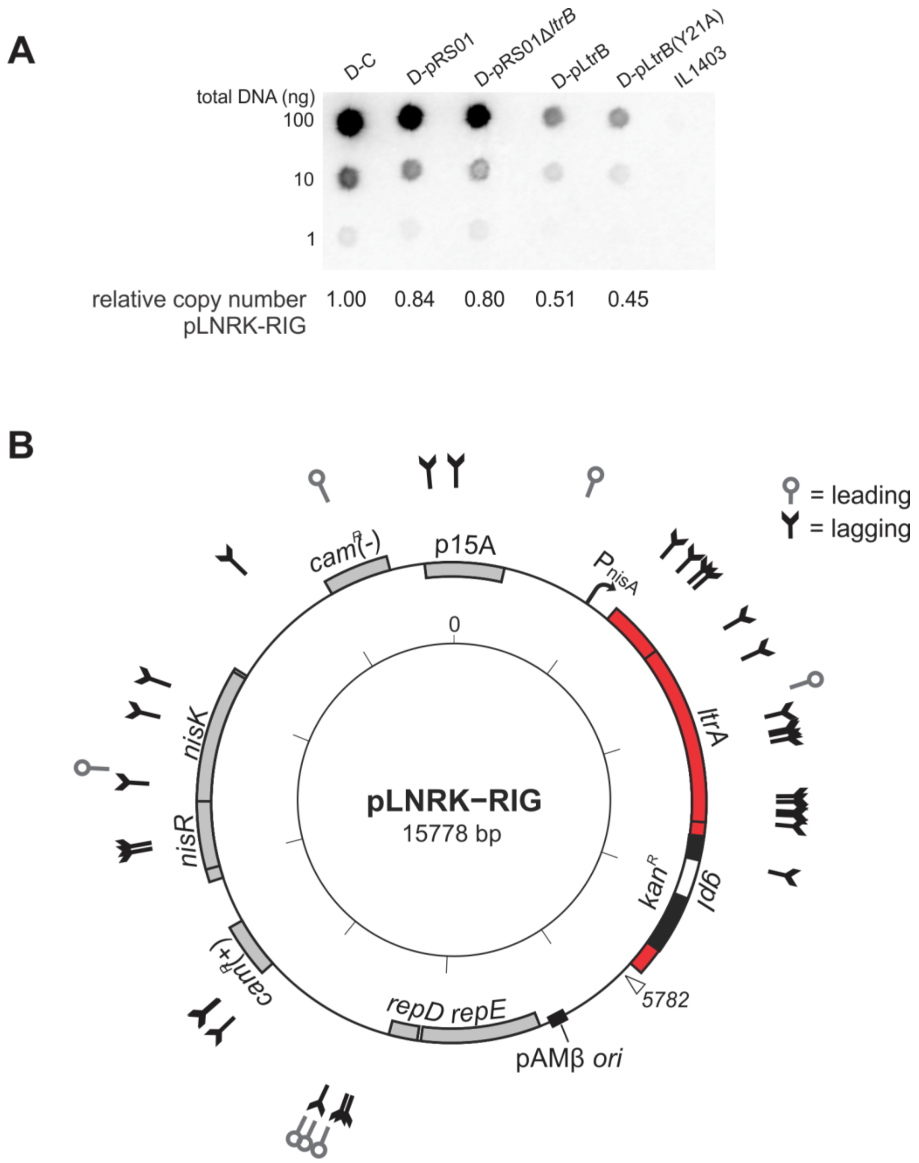

Figure S2. The relative copy number of donor plasmid between strains and mapping of the retrotransposition events onto the donor plasmid

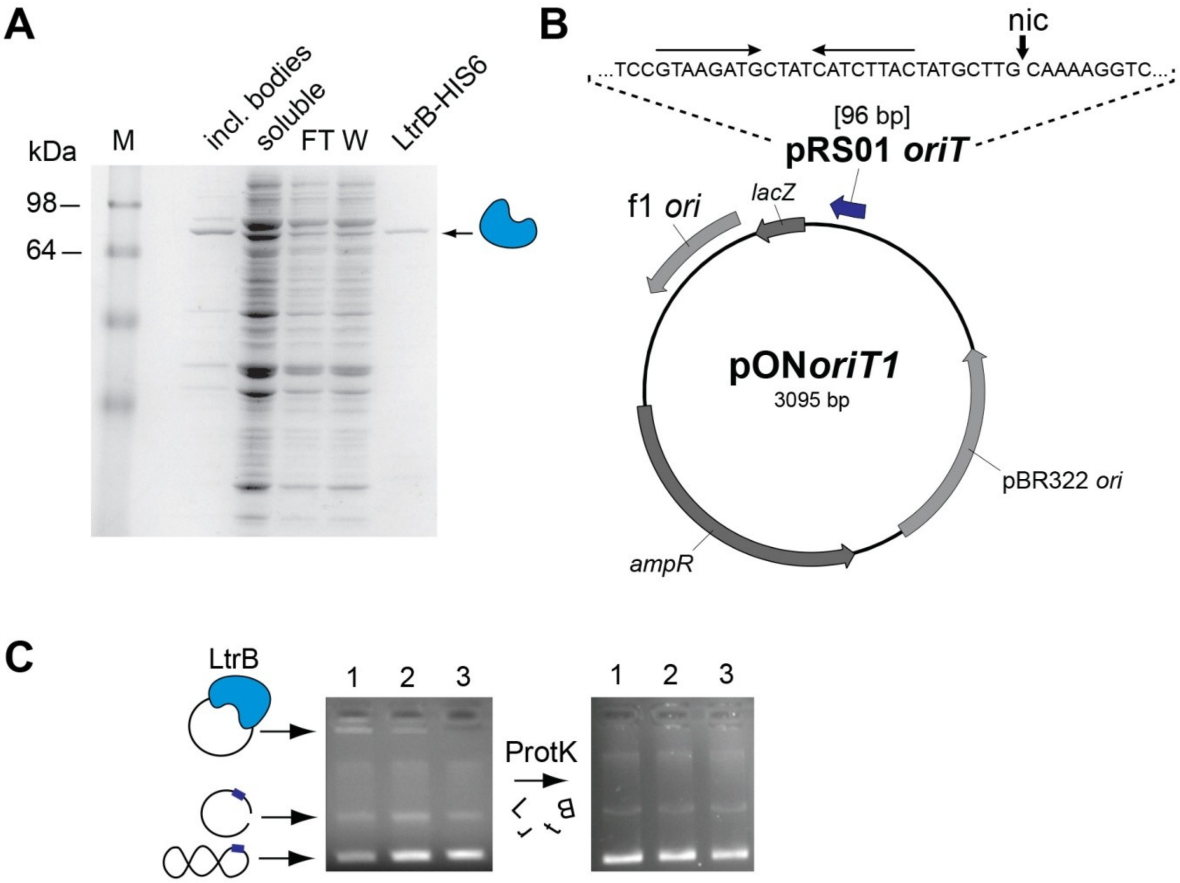

Figure S3. Purification of LtrB relaxase and its effect on dsDNA target

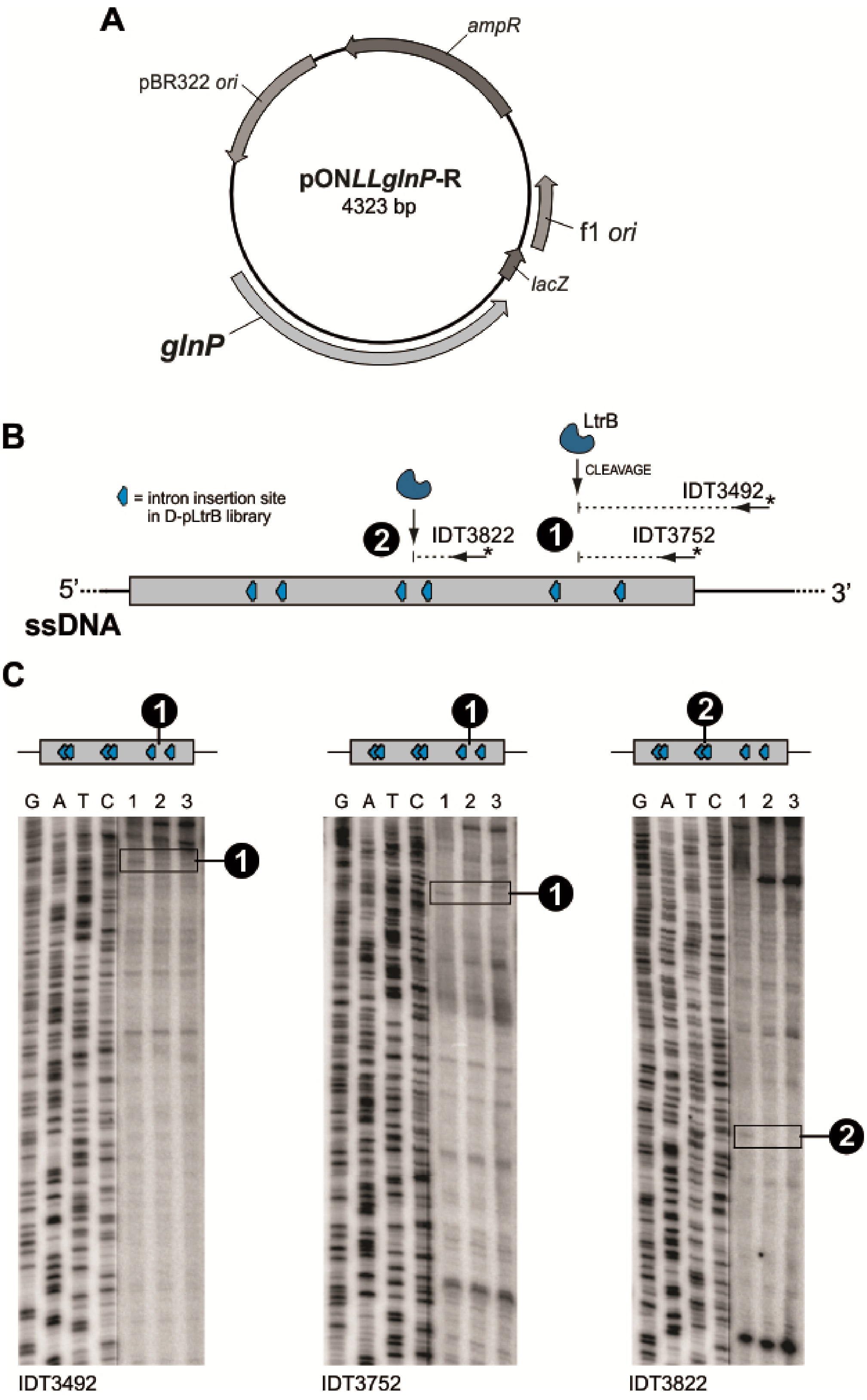

Figure S4. Mapping of off-target relaxase cleavage

Supplementary Tables

Table S1. Grid profiles based on the multiple sequence alignments of the chromosomal insertion sites.

C-6 | intron insertion (between -1 and +1) | T+5

D-C

	-25	-24	-23	-22	-21	-20	-19	-18	-17	-16	-15	-14	-13	-12	-11	-10	-9	-8	-7	-6	-5	-4
Cons.	A	A	*T*	T	*G*	*A*	*T*	A	A	A	C	C	T	T	T	T	T	*T*	T	*C*	*A*	*T*
A	31	54	13	23	40	44	25	37	47	60	12	26	31	19	33	9	28	21	22	0	52	3
C	29	18	22	10	14	12	26	32	5	16	39	32	16	23	6	26	20	12	18	97	6	13
G	22	10	20	29	41	7	18	28	20	10	22	17	12	18	24	8	4	3	20	0	7	3
T	18	18	45	38	5	37	31	3	28	14	27	25	41	40	37	57	48	64	39	3	35	82

	-3	-2	-1	+1	+2	+3	+4	+5	+6	+7	+8	+9	+10	+11	+12	+13	+14	+15	+16	+17	+18	+19
Cons.	*A*	*A*	*C*	*C*	*A*	*T*	G	*T*	A	T	*T*	A	G	A	*T*	T	*A*	*T*	A	A	*T*	*A*
A	51	76	2	5	76	16	13	35	46	22	38	40	31	42	16	22	56	17	39	41	32	54
C	18	1	73	61	14	25	20	20	6	25	16	22	7	16	29	29	3	22	18	27	26	7
G	22	1	1	11	3	9	48	7	18	22	6	10	44	3	7	17	20	25	19	16	9	3
T	9	22	23	23	7	50	18	37	31	31	40	28	18	39	49	32	20	35	24	17	33	35

D-pRS01

	-25	-24	-23	-22	-21	-20	-19	-18	-17	-16	-15	-14	-13	-12	-11	-10	-9	-8	-7	-6	-5	-4
Cons.	A	T	G	G	A	*A*	*T*	A	A	C	A	*A*	T	*C*	T	T	T	*T*	A	*C*	*A*	*T*
A	55	17	11	13	46	45	30	36	52	20	36	51	25	23	16	10	23	24	34	7	55	2
C	13	34	8	17	30	4	23	27	19	31	35	6	7	34	11	20	31	1	27	92	2	8
G	19	8	47	53	13	11	2	23	11	28	12	24	19	13	13	22	2	0	20	1	20	16
T	12	41	34	17	11	41	45	14	18	20	17	19	48	30	60	48	43	75	19	0	22	73

	-3	-2	-1	+1	+2	+3	+4	+5	+6	+7	+8	+9	+10	+11	+12	+13	+14	+15	+16	+17	+18	+19
Cons.	*A*	*A*	*C*	*C*	*A*	*T*	C	A	T	T	A	C	*T*	A	A	*A*	T	*T*	C	A	A	C
A	47	80	0	12	81	19	7	39	27	18	41	20	25	40	31	39	30	33	20	37	36	27
C	16	16	75	65	14	28	46	13	18	27	19	30	23	17	27	14	17	23	40	23	18	34
G	4	5	1	7	0	18	19	23	27	18	18	23	18	19	17	16	16	8	11	6	16	28
T	34	0	24	16	5	35	28	25	29	37	22	27	34	24	25	31	37	36	29	34	30	12

D-pLtrB

	-25	-24	-23	-22	-21	-20	-19	-18	-17	-16	-15	-14	-13	-12	-11	-10	-9	-8	-7	-6	-5	-4
Cons.	A	A	*T*	T	*G*	*A*	*T*	A	A	A	C	C	T	T	T	T	T	*T*	T	*C*	*A*	*T*
A	33	53	13	24	39	45	24	39	47	58	14	26	30	19	33	9	29	22	23	0	51	2
C	28	18	23	11	14	12	26	30	6	17	39	32	16	24	7	26	20	12	18	97	7	14
G	21	10	19	28	43	7	19	28	20	10	22	16	11	18	24	8	3	3	22	0	6	3
T	18	18	45	38	5	36	30	3	28	15	26	26	42	40	36	57	47	64	37	3	36	81

	-3	-2	-1	+1	+2	+3	+4	+5	+6	+7	+8	+9	+10	+11	+12	+13	+14	+15	+16	+17	+18	+19
Cons.	*A*	*A*	*C*	*C*	*A*	*T*	G	*T*	A	T	*T*	A	G	A	*T*	T	*A*	*T*	A	A	n	*A*
A	49	76	2	7	76	16	13	34	46	22	38	40	31	43	16	23	54	16	38	40	32	53
C	19	1	76	59	14	25	20	20	6	25	16	22	7	16	29	28	3	24	18	26	26	7
G	22	1	1	11	3	10	49	7	18	22	6	10	43	3	7	18	22	26	20	16	9	3
T	10	22	21	23	7	49	18	39	30	31	41	28	19	39	48	31	21	34	24	18	32	36

Legend **10%** **25%** **50%** **70%** **90%**

Integration sites of the Ll.LtrB intron were mapped in the absence/presence of the relaxase. Each cell represents the score of a particular nucleotide (A, C, G, or T) in the corresponding position shown as a percentage (%), color-coded according to the key at the bottom, from the total number of the sequences in the alignment. The nucleotides in consensus sequences that are identical to the nucleotides of the homing site in the corresponding position are highlighted with *italics*. The most conserved position C-6 and position T+5, important for endonuclease activity, are shown on the top. The intron insertion site is indicated by arrow.

Table S2. Multiple alignment of the chromosomal insertion sites of Ll.LtrB intron in the absence of the relaxase (D-C strain). Names of the genes and relative frequencies are given in which insertions of Ll.LtrB were detected; the orientation of the insertions relative to the direction of the replication (leading strand, lead; lagging strand, lag) and transcription (sense, S; antisense, AS) as well as genomic coordinates are indicated on the right. The sequence of the homing site and location of the insertion are shown on the top. T+5 is highlighted in red.

	IBS2 IBS1 (insertion ⇩)					
homing_site	CGTCGATCGTGAACACATCCATAAC	CATATCATTTTTAATTCTA				
citE(104.7E-3)	CAGGATAAGACTTGGTTTTCATGTC	CACGCGGTAGTCCATACCA	lead	AS	1208563	1208578
malG(90.6E-3)	CATTGACGTAGAATTTTTGCATAAC	TATGAATAAGATTGTAATA	lag	AS	1742504	1742519
yafC(63.5E-3)	ACAAGTTCAGCCTCATTACCATAAC	CATGTTCTGACTGACTAAA	lead	AS	55665	55680
citF(57.5E-3)	GATTACCGACCGCATCAATCTTAAC	CACGTAATCGACTTGCGTT	lead	AS	1209659	1209674
ywfG(49.4E-3)	TGCTGAGCAATCGTATTTCCTTGTT	CATCCACATAATTAGCAAT	lead	AS	2258691	2258706
ysjA(48.7E-3)	AACTCAGCAATTTATGCCACTTCAC	GCTCACTCTATTCACGCAT	lag	S	1890475	1890490
arcA(37.0E-3)	ACCCATTACCAAATGCCTACTTCAC	ACGTGACCCACAAGCTTCA	lag	S	2115042	2115057
pi134(36.8E-3)	AATAGATATTGCTCACACACATAAC	TTATTTAATTATATGAGGT	lag	S	464964	464979
ytiA(36.3E-3)	TTTAGAGAAAACATCTATGCCCTAC	CATGTATTATTGGTGGAAT	lag	S	1986494	1986509
ybeH(17.4E-3)	GATGATTATACCATACGGTCATAAT	CATAAAAAGCTCAAAGCA	lag	AS	143096	143111
ydiA(16.6E-3)	GAGAAGTCTATGATGTATTCAAAAT	TCATATCATTTTTAAAACA	lag	S	381887	381902
llrB(15.3E-3)	TCTGATACAATAGTTACTTCGTAAT	CATTTTGCCGAAAATTATA	lag	AS	1458230	1458245
yljJ(14.0E-3)	AATGTTCATCTTCTAAAAGCTCTTC	AAGATTAATGACAGCTTCA	lead	AS	1200004	1200019
xynB(13.3E-3)	ATGGACAGACCCAATCAAGCTCAAT	GGTCTTGGCTTTGACGCTT	lag	S	1544533	1544548
purA(13.1E-3)	GTTCCATATTCATGTCCTACTTCAC	GAATTTGATGTCCAACTTC	lead	AS	2029172	2029187
malG(12.7E-3)	AACACGTAGAAACCTAATCCAGAAC	CACCTACATAGACCAGTGC	lag	AS	1742154	1742169
ypjC(11.5E-3)	ATTCAACGACTGTCACAATCACAAT	CGTATTAAGATAAAGGCTG	lead	AS	1592521	1592536
rexB(11.4E-3)	ATTTCGCATTTTTCAATCCTGCTAA	TAAAATATCTAAAAACTCC	lead	AS	4716	4731
ysdA(10.0E-3)	GTCCGATGATTCCCACATACATCAT	CATCAATGTGAAAATTAAA	lead	AS	1834288	1834303
ypdA(9.5E-3)	CGGCTAAGATTGTCAAAATCGTCAT	CAGTGGAACAACTTGAGGA	lead	AS	1530265	1530280
intergenic(9.5E-3)	TATCCAAAATAAAGTTTTCCCAAC	CAAGGATTACTCGTTTCAA	lag	.	2064332	2064347
oppA(9.4E-3)	GTGGGCAAGATGGTCTCTTCTTTAC	CAACAGTTCATTTAAATAT	lag	S	1906589	1906604
yxdC(9.3E-3)	TTCGGATAATGGCTTTTTTCTGAGC	CATATTTTCTACACCGTAA	lag	AS	2335310	2335325
ymgK(9.1E-3)	GCCTCTCCAACTGCTCCTTCGCTAT	CATAATTAGTTGAGGTATC	lag	AS	1268130	1268145
ybhB(8.9E-3)	ACATTTAATAAAACATGTGCCCAAC	CAATATGGATAAAGAGTGC	lead	AS	173543	173558
intergenic(8.9E-3)	GGCTGATTAAGCACATTTTCATTTT	CATTTAGGACTGATTTTAT	lead	.	1880376	1880391
murC(8.2E-3)	GTAATTTCATGATGGTCTACTTCAC	GTGCTGAACCGTAAATTTG	lead	AS	2118446	2118461
ycjH(8.1E-3)	CGTAAAATAATGCCAGCTTCCTCAG	CCAAATTAACTAAATATTG	lag	AS	295716	295731
intergenic(7.5E-3)	GTTGTATCTACGCCAATTGCATAAA	CACAAACTATCTGCACCAA	lead	.	1891667	1891682
yudI(7.4E-3)	GCTTCAACCAGAGTATCTACTTCAC	CCCCAAAAATTTGCATTGA	lag	AS	2036117	2036132
ykjI(7.2E-3)	GTTGACTGTCCCCTTTTTTCATGAT	TATTTTTCCATTAAAAGTT	lead	AS	1098929	1098944
ypcA(7.2E-3)	TCTTGATAATGGTCAAAATCATAAT	CAAAAACATTGTCATGCTG	lead	AS	1521196	1521211
ldhX(7.0E-3)	ATAAAGATATCCAAACCTACTTCAC	GAATTTCTTGATCAATTTC	lead	AS	1143058	1143073
rpsI(7.0E-3)	TGTGGGATGTAGCTTTCTACTTCAC	GTCCGTTAACTGTGATTTT	lead	AS	2346610	2346625
celB(6.0E-3)	GTTGACCCAGTTGTCAATGCTGTGC	CATGGGTAACACCACCAAT	lag	S	178859	178874
pi242(6.0E-3)	GCCCTTCGGTTGCGACTACTTCACG	TTGAAAGGCACGATATTGT	lead	AS	1061365	1061380
ymeA(5.8E-3)	CGCTGACGGTTCTTTTATCTACAAC	TAAAATGCTCACTATTCAA	lead	S	1242476	1242491
lcnC(5.3E-3)	TTAGGCGCTAGTGAAGCTACTTCAC	AAGAAGAGATTATTAGAGC	lag	S	84331	84346
sigX(5.3E-3)	GATTAGAGGAGCCACTGGTCATAAC	CAGCTTTTTCAAGTTGTTT	lag	AS	2242934	2242949
als(5.2E-3)	ATTGCTTCAGCATATTTTACGTAAT	CAACATAGCCAAAATCAAC	lead	AS	1201917	1201932
gltD(5.1E-3)	TTAGGAGCAGCTTCGGTTTCTCAAT	TAGAAATCACACCAAGTTT	lag	S	1315536	1315551
ychH(5.0E-3)	TGAGCCAGGAGCAATTATTCGTGAC	CAAGTAACTATTGGCGATA	lag	S	279638	279653
uvrB(5.0E-3)	AAAATTGCCAAATGGTCTACTTCAC	CTAAAACTTGTCCGGTCAA	lead	AS	556835	556850
yljE(4.9E-3)	TCGACGGACAACAATATTTCGCAAC	CAACCTTTTCTTGTTTTCT	lead	AS	1194711	1194726
yidC(4.8E-3)	TTTGGAAACGATACATATTCATATT	CATTTCTTTCATAAGATCT	lead	AS	833054	833069
apu(4.6E-3)	TATTTACGATTCTCAGGGTCTTTGC	CACCTGACAATCCTGCTTC	lead	AS	703842	703857
L200119(4.6E-3)	CAAGCCGGTGTGTTAACCACTTCAC	CACATCCGCCATAATGAAA	lead	AS	2340666	2340681
yyaL(4.5E-3)	ACGTGAATGATGGCATCTACTTCAC	GAATATTGGCAAGAAATTT	lead	AS	11386	11401
yriC(4.5E-3)	GGAAGGCTACGGTCATATCCATATT	CAATTAAATAACGGTAAAC	lead	AS	1787588	1787603
cysD(4.2E-3)	TCAGGAAAATTACCATTTGCCCAAT	CAAAGTTTCCGCCATCAAC	lag	AS	76567	76582
yoaB(4.0E-3)	TGAATGGATTGACCCAGCCCACGAC	CAAAGCAAAGACAATCGCA	lead	AS	1403668	1403683
ysaA(4.0E-3)	GGACCTGTCATTTTTGCTACTTCAC	TCACAGGAGGAAGCGGGAA	lead	AS	1806395	1806410
L200143(3.9E-3)	AGCGCTTATCCCTTCCCTACATAGC	TATCCAGCCGTGCTCCTGG	lead	AS	542122	542137
yfcI(3.8E-3)	TATCTCCTGAGCAATTCTACTTCAC	ATCTCATTATTGATCCTGG	lead	S	529084	529099
menD(3.8E-3)	AAGGGAACATTGATTTGCACAGGAC	CCGAAAGAGGGGAAATAGC	lag	AS	737583	737598
codY(3.7E-3)	GAAGCGATAACATGCCCTTCTTCAC	CATCAAGCTCTTCAATGAT	lead	AS	164615	164630
yqgG(3.7E-3)	GAAGGATCAAGATTGACTCCATGAG	TACTGATGATGTCCATAAA	lag	AS	1664217	1664232
noxD(3.4E-3)	GGCGGGTCAAAAATCTGTCCTTAAT	CAATCTAGTTATTCTTCGG	lag	S	2195930	2195945
yriB(3.2E-3)	CTGAAGCAGTTGTGATGTTCTTAAT	CATACCAATGATAATCGGT	lag	S	1786760	1786775
dnaA(3.0E-3)	TGCTGTCGATGTCTTTTTGAACATC	CATATCATTTTTCATCTTA	lead	AS	1668	1683
icaC(2.7E-3)	GTTGAGAAAGCCCTCCATCCGTTGC	CCTCGTTGAATAATCGATA	lead	AS	684217	684232
ptnC(2.7E-3)	AGGAAGAAGAATGGCCATACTTCAC	GAGTAGCCATAAGGTTGAT	lag	AS	1763895	1763910
gatB(2.5E-3)	CTATTCAAATATTGAGCCACTTCAC	CTTGCAACCAGTTTGACAC	lead	AS	168767	168782
pfl(2.5E-3)	TTCAAATTCTTGATGGATACTTCAC	ACCAGGTGCTTTGATTAAT	lag	S	659540	659555
hemH(2.4E-3)	TGCAGATTGTTTAGAAACTTTATTT	GAATTAGAAATTGAAAATA	lag	S	1608898	1608913
yreB(2.4E-3)	CTTTCCATACTTCATTAACTGAATT	CATATTATTTTTAATTGGA	lag	AS	1744102	1744117

clpE(2.3E-3)	ACACGTTTAATTTCTTCATCACGCC	CAATCACTGGGTCAATTTC	lead	AS	550771	550786
yidC(2.3E-3)	TTGGATTGTTTTTAATGAGCATAAT	CTTTATTTTGCTGACGGTT	lag	S	833363	833378
ycdJ(2.1E-3)	ATATAATTCGGCACATATTCATAAC	CATTGGCATAATAAGTCGT	lead	AS	239415	239430
intergenic(2.0E-3)	TGTTGTAGTTTATTACCTACTTCAC	TTCTATTCTAATAGATATT	lead	.	1299735	1299750
yijC(1.9E-3)	GAACCTCTGCCCATTCCTCCTTCAC	GTACACCAAATCCACCAGC	lead	AS	893748	893763
glpD(1.8E-3)	ACCTTGATAGCATCTACTCCTTCAC	GCATAAACAAGATGTGGTT	lead	AS	1270237	1270252
mgtA(1.8E-3)	TGGTGACAAGCATCATATACATCAC	TACTGTTAAAATTAGCCAT	lead	AS	1285837	1285852
yxeA(1.8E-3)	TTGGTGATAAAATTAAATTAGCAAC	CAATAATGAAGTATTGACT	lead	S	2348003	2348018
glgD(1.6E-3)	TGATGCCAATATGGTCATGCTCGAC	CCCCAAAATTTTACCTCTC	lag	S	697924	697939
pspA(1.6E-3)	TCAAAGAGATACCATTGGCCATTAC	TATCTTTTTGTTGTCCATA	lag	AS	2304139	2304154
pspA(1.6E-3)	CCATTATATAATGCCACTACTTCAC	AGGCTTGATAAGCTCCGAT	lag	AS	2304622	2304637
intergenic(1.5E-3)	TATATAAGTCCCTGGGCTACTTCAC	TTTTGGTTGAAAGTAGCTC	lead	.	133745	133760
L200091(1.5E-3)	GCAGAGCCATGTGTTACCACTTCAC	TACGCCGCCACAACATTAA	lead	AS	424310	424325
pi234(1.5E-3)	TTCATCCATAATTTGCCTCCTTCAC	GATTTGAACATAGCGATCA	lead	AS	1058576	1058591
yrjC(1.5E-3)	GGATAAATCGAACCATAAGCATGTC	CACCAATTTGACGATAAAC	lag	AS	1792740	1792755
yfbJ(1.4E-3)	AATAGTCTATATAATTCCACTTCAC	ATTGGCATAAAGTAGATAA	lag	S	515575	515590
ysgC(1.4E-3)	AATGTATTATAGCGAGCCACTTCAC	GCGCAATTGCCGCCGGAAT	lead	AS	1870118	1870133
intergenic(1.3E-3)	CGTAAATGCGCTAAAGCGCAAACGG	CTATCCTATGACAGAAGTT	lag	.	235061	235076
ycdJ(1.3E-3)	TTAGAGGCAAGTTCACCAACTTCAC	AAGCAAGCTTAGCTCCAAG	lag	S	237900	237915
ydiB(1.3E-3)	TTTGCTAGCCCTCCTTACACTTCAC	CAGGAGCTTTGAAGAAAGC	lag	S	383788	383803
pheS(1.3E-3)	GTTTTACTTCGTACGCATACTTCAC	CTATGCAAGCTCGGACAAT	lag	S	2009589	2009604
yudK(1.3E-3)	TCAGTTCCTGAATAGGCTCCTTCAC	CGTGTAAATATGTTGCGCT	lead	AS	2038474	2038489
preA(1.2E-3)	GCAATCGCTTCATCATTTACTTCAC	GAGCCAAGAGTAAAGGTAA	lead	AS	187014	187029
nrdF(1.2E-3)	TCATCAGACATGGCTTCCACTTCAC	CAAGAAGGTATGAATTACC	lag	AS	1001348	1001363
intergenic(1.2E-3)	AAAAGTTCTTTAAAATAGATTTCTT	CATATCATTTTTATTTACT	lead	.	1677069	1677084
L200065(1.2E-3)	TGAAGATAAAACCTCACAACTTCAC	TAGATAATTCCTGTTTTCT	lead	AS	1745995	1746010
recO(1.1E-3)	ATATTTTTATAAACGACCACTTCAC	TGACATCCTCGATAAAAGA	lead	AS	58059	58074
citB(1.1E-3)	CCTGATAATCGTGTTGCTACTTCAC	TTAAATCAACATTAATCGT	lead	AS	669535	669550
yrjD(1.0E-3)	TTTCTTTACCAATTTTCCACTTCAC	CAATTGGTCTGAAATAACA	lag	AS	1793542	1793557
fbaA(1.0E-3)	TCACCACCAATTGAACCAACTTCAC	ATTCAACTGAGATGCCTTT	lead	AS	1979895	1979910
hpt(996.6E-6)	ACATCTAGGATTAATTTTACTTCAC	CAGAGGATTTTGTTCCGCC	lead	AS	25358	25373
L200159(988.4E-6)	TTTACGATCCGAAAACCTTCTTCAC	TCACGCGGCGTTGCTCGGT	lead	AS	2364671	2364686
rpoC(980.2E-6)	GAACGGTAAGTTTTTGTACTTCAC	CGAGCAAGTAAGTTTCAAC	lead	AS	1857655	1857670
noxB(963.8E-6)	AACTTTATGCGTGGTCATACTTCAC	GTCAAGGAAATGTACTTTG	lag	S	842595	842610
hrcA(951.5E-6)	ATAAATTGATTTGTTCTTACTTCAC	CAGTCCCTAATGTTATAAC	lead	AS	977222	977237
yxbD(951.5E-6)	GCAGGTCACGTTGTCCCTGCTTCAC	GTCGTGGTGAAGGAATTGG	lag	S	2319277	2319292
rpsH(918.7E-6)	TACCAGATGTAGGCAATTACTTCAC	CACCAACATTTGTTTCACG	lead	AS	2162238	2162253
scrK(881.8E-6)	GCGGAAAATCATTGGTTTACTTCAC	AATTGGAACAGGAATTGGT	lag	S	1518450	1518465
thrA(849.0E-6)	ACTTTTCGGCCGAATCCAACTTCAC	GGTTCATCAGATATTTACT	lag	AS	747685	747700
yfhL(832.6E-6)	CCTCTAAGTCCTCCTTTTCCATTGC	TGTTCATTTTTCCTTCAAG	lag	AS	579452	579467
hemH(803.9E-6)	AGTCTATTTTGGTCAACTACTTCAC	TTAATACTCTAGTAAAGTC	lead	AS	1608810	1608825
gidB(791.5E-6)	CCTCGAAAATGTGACTTTACTTCAC	GGACGAGCTGAAGATTTTG	lead	S	1381124	1381139
relA(787.4E-6)	TTTTGTTTGGCTCTCTCAACTTCAC	GACGTTCATTTTCAGTTAA	lead	AS	108865	108880
yabA(779.2E-6)	TCAGCGAACAAGCGATAATCGCAAC	TAACCAGTACCACCAGTCA	lag	AS	10402	10417
ssbB(754.6E-6)	GAAGTTCCGCCTCCCATACCTTCAC	GAGCAGATCTACTTTCCAA	lead	AS	2274356	2274371
asnB(746.4E-6)	GTTCGTAAGAAATCTGCCACTTCAC	GAGCATATTTCAAATCAAT	lead	AS	356847	356862
yeaC(746.4E-6)	TGGAGATCATCCTCATTTACTTCAC	GCACAATCGTGTGGTGAAT	lead	AS	405707	405722
intergenic(746.4E-6)	TGTGGTTCTAATACACATACATCAT	CATTAAATCGAACAATTGC	lead	.	1260552	1260567
fbaA(742.3E-6)	ACACAATGTGCCAATCGTACTTCAC	GGTGGTTCTGGTATCCCTG	lag	S	1979644	1979659
llrG(725.9E-6)	ATTTTTTCCTTGGTCACCACTTCAC	CATTTCCACGAAAAAGAAG	lead	AS	1805025	1805040
yveC(725.9E-6)	AAGTAACGACCTTCTGTAACAGGAC	CAGAAACGTAACCTACGAC	lag	AS	2143762	2143777
intergenic(701.3E-6)	CCCTCTGGGTCAGCTCGTACTTCAC	GCCGTCACTGCACATATTT	lag	.	2016727	2016742
ps105(693.1E-6)	AAGGGCTTCACTCCCTCATCTTCAC	ACCATGCACGATATAAGGC	lag	AS	37893	37908
zitP(680.8E-6)	AGAGTTTTAATGTCAGATACATTATC	ACATGTATCATTAGCCGGT	lag	S	2182929	2182944
ptnC(668.5E-6)	GAAGATGCAGTACCATTTCCAGAAC	CACCAGAAGCTTGGAGGTT	lag	AS	1764021	1764036
ptsI(643.9E-6)	GTAGATGGATCAAGAATTACTTCAC	CAGTCAATCCTGAAACAGC	lead	AS	121312	121327
yjiB(639.8E-6)	TGAAATTAGACATTATCTACATGCT	CATCCCGAAATATCAGAAA	lag	S	982384	982399
purA(635.7E-6)	GGTGTTTGTAAAGCCTATACTTCAC	GTGTGGGTGATGGTCCATT	lag	S	2029214	2029229
ydgC(627.5E-6)	CAGGCGTCAGGACAGGTTACTTCAC	TCAGTGGTCTTATTGGTTA	lead	S	362514	362529
L200142(623.4E-6)	TACGGCTACCTTGTTACGACTTCAC	CCCAGTCATCGGTCTTACC	lead	AS	539039	539054
fabD(607.0E-6)	GCATAATCAACCGCCTCAACTTCAC	CACCAATAACAATTTGTGC	lead	AS	783367	783382
alaS(598.8E-6)	TTAGCGTCAGGAACAGTTACTTCAC	TGGCGATAAAGTTGAGAGA	lead	AS	1778652	1778667
intergenic(594.7E-6)	TCTAGTTAAGAAATCCCTATTTCAC	ATAAAATAAAAGACCGTTC	lag	.	767701	767716
yqbK(590.6E-6)	TTACAGATTTATGCCAATACTTCAC	CAACTACTGGTACTTTTAA	lag	S	1619709	1619724
uppP(582.4E-6)	AAGGTAAATTCTGCGGCAACTTCAC	GACTTACACCAACTAATAA	lead	AS	2275479	2275494
ymeA(578.3E-6)	ATTCAATCGCAATTACTGACAGAAC	TGTCAGCTGCGCTGACGGT	lead	S	1242654	1242669
def(570.1E-6)	AAGGTTACATCATTTGCCACTTCAC	GTAAAGTTGGATAACCTTC	lag	AS	555416	555431
asnB(561.9E-6)	GTTTCTAAGTGCCAAATCACTTCAC	GGACTGCTGAAAGTACATC	lead	AS	356922	356937
rplM(553.7E-6)	AGTATATTACTCACACTCACTTCAC	CCAGGTGGATTGAAATCAG	lag	S	2346912	2346927
nrdD(549.6E-6)	TCATAGTTTGGTGTCCCTACTTCGA	GGTTAAGACCACGTTTGAG	lead	AS	271457	271472
ilvD(549.6E-6)	TCGATGCTGTCAGCAATCACTTCAC	GACTGACCAAAGAATATCT	lead	AS	1246083	1246098
pi301(541.4E-6)	TTCTGCAGATACAGATCCACTTCAC	GAAAGATTATATAGAGTTT	lag	S	1414528	1414543
ysjD(541.4E-6)	TTTTTTACCTTGTCAGCCACTTCAC	CTGCTTCACCAGATAATCC	lead	AS	1893720	1893735
comFA(537.3E-6)	TCAACAGTGAGCACCCAAACTTCAC	TAAATCCGCACTGATACAA	lead	S	1097125	1097140
pstC(529.1E-6)	TCACTTCGAGCAATTCCAACTTCAC	AACGTGAGGGTGGTTTGGC	lag	S	1773284	1773299
rgpA(520.9E-6)	CAGGATTTGTCCCCCCTACCTCATG	ACCATGTAAATAAGCAAAG	lead	AS	202268	202283
ybcH(512.7E-6)	AATGATACTTATGTCCCTACTTCGA	TGGTTTACGAAAATTATAA	lead	S	123666	123681
intergenic(512.7E-6)	ATACCGCGGTACCACCCTTATTCAC	AATAGAATTTCTATCATCT	lead	.	1781130	1781145
L200142(508.6E-6)	CTTGCGGTTAGGCAACCTACTTCGG	GTACTCCCAACTCCCGTGG	lead	AS	538980	538995
gyrA(496.3E-6)	AAGCGGCGAAATATCTCTTCTTCAC	AACACGTAACGGAATTGTT	lag	S	1123616	1123631
ycfD(492.2E-6)	AAGCCGCCTAATCCATCTCCTTCAC	CATTAAAAAGACGAAAGGC	lead	AS	252972	252987
yujG(475.7E-6)	AACAGTAGAGCAACACTTTCTTAAT	CATTTCTCTAACTTCAGTA	lead	AS	2099884	2099899
yfjF(471.6E-6)	CTTCAAAGTGACTTACATACTTCAC	TAGACATGATTCAGTGGGC	lead	S	599610	599625
ymcD(471.6E-6)	GTATGGTTTGAGCCATATCCGCAGC	ACTCACTTCTTTTTCTCGG	lead	AS	1221884	1221899
ponA(426.5E-6)	AATCCATATACTAACCCTACTTCTG	CTAAATGGCGCCGTGATAT	lead	S	530491	530506
leuA(418.3E-6)	ATTCATGTTTTCATTGCCACTTCAC	CTATTCACATGAAATATAA	lag	S	1240133	1240148
intergenic(410.1E-6)	TGACAAACTCCCAAAAGAGTGACCC	CACCAATTGAACCAAAGTT	lead	.	426554	426569
clpC(410.1E-6)	ACACTTGCTTTATGCCCTACTTCAA	GTAGAAGATGGATTTGGTC	lag	S	630146	630161

Table S3. Multiple alignment of the chromosomal insertion sites of Ll.LtrB intron in the presence of the relaxase (D-pRS01 strain). Names of the genes and relative frequencies are given in which insertions of Ll.LtrB were detected; the orientation of the insertions relative to the direction of the replication (leading strand, lead; lagging strand, lag) and transcription (sense, S; antisense, AS) as well as genomic coordinates are indicated on the right. The sequence of the homing site and location of the insertion are shown on the top. T+5 is highlighted in red.

	IBS2 IBS1	insertion				
	CGTCGATCGTGAACACATCCATAAC	CATATCATTTTTAATTCTA				
L200140(43.5E-3)	ACGGATTTCGAGTCCGCTGCATTAC	CACTCTGCCATGGCTCCAC	lag	AS	126580	126595
menB(42.1E-3)	ATGGAACAACAGTATTAACCATTCC	CATGTCCATTGCTTCTTGG	lag	AS	734626	734641
als(41.8E-3)	ATTGCTTCAGCATATTTTACGTAAT	CAACATAGCCAAAATCAAC	lead	AS	1201917	1201932
citE(37.1E-3)	AGGATAAGACTTGGTTTTCATGTCC	ACGCGGTAGTCCATACCAG	lead	AS	1208563	1208578
yccB(34.0E-3)	GTGGATAATACATTTTTTTCGTTAC	TATCAGTTGTAAATAAATG	lag	AS	220464	220479
arcA(28.6E-3)	ACCCATTACCAAATGCCTACTTCAC	ACGTGACCCACAAGCTTCA	lag	S	2115042	2115057
malG(25.1E-3)	CATTGACGTAGAATTTTTGCATAAC	TATGAATAAGATTGTAATA	lag	AS	1742504	1742519
clpP(20.4E-3)	ATGGCAAGACCAGCAGATACAGAAC	CACCAGGTGTATTTACGTA	lag	AS	672765	672780
ycgD(16.5E-3)	GCTGAATCATAAACTCTACCATAAT	CACCAGCAACCGTTGGAAT	lag	AS	263794	263809
ydgC(14.3E-3)	CCATAGAAGTAAAGGCAAACATAAC	CATTTGTAAGGCACCAACA	lag	AS	362156	362171
hrcA(14.2E-3)	TCTGCAAGATTGTCAGTTGCATAAT	CAAAGATATTTTTACGCCC	lead	AS	977477	977492
purA(13.5E-3)	GTTCCATATTCATGTCCTACTTCAC	GAATTTGATGTCCAACTTC	lead	AS	2029172	2029187
intergenic(11.4E-3)	TTGCTTTAGCCTCTTTTTCCACAGC	TACAGCTTGGAAAAGATAT	lag	.	492183	492198
yljE(9.9E-3)	TCGACGGACAACAATATTTCGCAAC	CAACCTTTTCTTGTTTTCT	lead	AS	1194711	1194726
L200158(9.6E-3)	CAGCGCTTATCCCTTCCCTACATAG	CTATCCAGCCGTGCTCCTG	lead	AS	2360508	2360523
ponA(9.5E-3)	GATTGACTTATTGTTTGACCAGAAC	TATCTGTTTTAACAAGAGC	lag	AS	529213	529228
ypaE(9.3E-3)	CTGGAGCGCTTTTGCCTATGCTAGC	CATTTCTTTAATTCCTAAA	lag	S	1505183	1505198
ysfA(9.0E-3)	ACGGACCAGTCAACTGTTGCATTAT	CACCAATCAATCCGCGACG	lead	AS	1850611	1850626
murC(8.6E-3)	GTAATTTCATGATGGTCTACTTCAC	GTGCTGAACCGTAAATTTG	lead	AS	2118446	2118461
tgt(7.9E-3)	GATTGACGACGTAAATCTTCAAAAC	CAGCTCCTTGAACAATTCC	lag	AS	155934	155949
yveC(7.7E-3)	AAGTAACGACCTTCTGTAACAGGAC	CAGAAACGTAACCTACGAC	lag	AS	2143762	2143777
ldhX(6.7E-3)	ATAAAGATATCCAAACCTACTTCAC	GAATTTCTTGATCAATTTC	lead	AS	1143058	1143073
yljG(6.5E-3)	AAGTCTTCAGATTCTCTTTCGTAAT	CATGAGTCTTTTGAGCCAT	lead	AS	1197156	1197171
rpsI(6.4E-3)	TGTGGATGTAGCTTTCTACTTCAC	GTCCGTTAACTGTGATTTT	lead	AS	2346610	2346625
menF(6.3E-3)	AAGCATAAAGTTTCTTGTCCACAAT	CAAAGCCGAACGAATCCCG	lag	AS	738249	738264
proC(5.8E-3)	TCTTGAACTGTAGCATAAGCAGAAT	CATCAACAAATTCATTTTT	lag	AS	1953085	1953100
ygdA(5.6E-3)	TTAACATAGGCTGTTACTTCATGAC	CATCGTTGAAATATTTATC	lead	AS	632471	632486
intergenic(5.5E-3)	ATAATCTCTTGGATTACTTCACAAT	CATTTTATCAGATAATAGA	lead	.	1200372	1200387
yahG(5.5E-3)	GCAGCGTCAGCTTCGGCTTCATAAC	CACCCATTTCGCCAAATTC	lag	AS	74462	74477
yjgC(5.4E-3)	GCACGAGCAACCTCAATATCGTAAC	CCGTTAAATTTCCTTGACT	lead	AS	962423	962438
yudI(5.3E-3)	GCTTCAACCAGAGTATCTACTTCAC	CCCCAAAAATTTGCATTGA	lag	AS	2036117	2036132
pi242(5.3E-3)	CGCCCTTCGGTTGCGACTACTTCAC	GTTGAAAGGCACGATATTG	lead	AS	1061365	1061380
citE(5.3E-3)	CCTCAAAAATTCCTGCATCCTTAAT	CATGGCTGGGTTATTCCCA	lead	AS	1208134	1208149
llrB(5.2E-3)	TCTGATACAATAGTTACTTCGTAAT	CATTTTGCCGAAAATTATA	lag	AS	1458230	1458245
glk(5.1E-3)	AATTCTCCAGCAGCAGAAACACCAC	CACCGATGACGATTGAAGC	lag	AS	2101484	2101499
queA(5.1E-3)	TAAGCGTCAAGAACAAAATCACGGC	CAGCAAAAGCAGCGACTAA	lag	AS	1617701	1617716
malG(5.0E-3)	AACACGTAGAAACCTAATCCAGAAC	CACCTACATAGACCAGTGC	lag	AS	1742154	1742169
citE(4.9E-3)	CTGCATCTTCCAAGTCAAACATAAT	CGAGTCAGCCTCAAAAATT	lead	AS	1208167	1208182
yrbA(4.8E-3)	TCTTGATAGTCAAGAATTCCAACAC	CAGCTAAATCAACGCCTGA	lag	AS	1710778	1710793
ppiA(4.7E-3)	TTGTATTATTTTTTTTATTCGTATT	CATAGGAATATTTTAACAT	lag	AS	369301	369316
pspB(4.6E-3)	CATTGACCTGTCCTTTTCTCATGAT	TCCAGAAGAGCGATCAAAG	lead	AS	2306115	2306130
ydaG(4.6E-3)	TGATAATGGCAAACATAATCATTCC	CATATAAGTCATGAATGTA	lead	AS	309995	310010
noxA(4.3E-3)	CATGATTTTCTTCCCAATCGCTTC	CATGTACTCATTTGCTTGA	lead	AS	840783	840798
rliA(4.3E-3)	TTAAAGACAAGACATCATCCATAAC	TACAAGAGCTGTTTCATTT	lead	AS	1728090	1728105
clpE(4.2E-3)	CATGAATTTCATCAATAAACATAAT	CACATCATTACGTTTTCTG	lead	AS	551043	551058
ponA(4.2E-3)	AGAGGATCTAAAGTAATTCCTAGAC	CATTAACAAATTTGCTAGA	lag	AS	529831	529846
L200119(4.2E-3)	CAAGCCGGTGTGTTAACCACTTCAC	CACATCCGCCATAATGAAA	lead	AS	2340666	2340681
yjgC(4.1E-3)	TTGCCAAAGAGGAAATATTCATAAC	CGTCATGTTCAGCATCACC	lead	AS	962933	962948
ydgF(4.0E-3)	TCTTGTCTAATGCAGCAAGCCCAAT	TGGAAGATGTTGCTCATAA	lag	AS	364642	364657
glk(4.0E-3)	TCACCTGAAGCGATAATTCCGCCAC	CAACACCAGTTCCAAGAGT	lag	AS	2101115	2101130
apt(3.9E-3)	GTAGCAGCATAGTTATAGGCATTTC	CGTCTGCCATCAAAGGGCT	lead	AS	622901	622916
yccJ(3.9E-3)	TTAAAGTGTCCACGTTCATCATGTC	CAAAGACACTGACTACGAT	lead	AS	228492	228507
ycjH(3.9E-3)	GTAAAATAATGCCAGCTTCCTCAGC	CAAATTAACTAAATATTGC	lag	AS	295716	295731
gntR(3.8E-3)	GAATTGAAAGCCTCGCTGCACTATC	AATATTCGCAATTAAATAG	lag	AS	2271599	2271614
als(3.8E-3)	CCTGTGAAAAGGAAGCCTCCATCAC	CAGAGTGTGAATAAACTTT	lead	AS	1201755	1201770
yljF(3.7E-3)	AAAGCCGCAAACATTCCTGCGGGAC	CAAATCCAATGACTACAGG	lead	AS	1195835	1195850
argR(3.7E-3)	CATTCGCCAACACATCGGCCTCACC	CAATTTTGTCCGAATAACC	lag	AS	2117884	2117899
L200143(3.6E-3)	ATCACCTGAGGCTACTCGCCTCGAC	CACCTGTGTCGGTTTGCGG	lead	AS	541048	541063
clpE(3.6E-3)	TTAGTTCGTCCCTCACGAGCAGCAC	CAAATCCGACAGAGGCTTC	lead	AS	552211	552226
rpoE(3.6E-3)	ATGTCAACTTCTTCATCTACATTAT	CAAGTTCAACTTCTGCAAG	lead	AS	624031	624046
L200143(3.6E-3)	GCCTACTGCGTCCCTCCATCACGAT	CATATCTAGTACTGGAATA	lead	AS	540838	540853
rnhA(3.6E-3)	AGAAAATTCAAGTTTTCTTCACTGA	CAAAACTAGCAGTAACCAT	lag	AS	2349720	2349735
typA(3.5E-3)	ATTGACAAGTGCAATTCACCACGAC	CAGAAACAACCCATTTATC	lead	AS	2093418	2093433
ysaA(3.5E-3)	GGACCTGTCATTTTTGCTACTTCAC	TCACAGGAGGAAGCGGGAA	lead	AS	1806395	1806410
yvdB(3.5E-3)	AACCGATCTTTTTTCGCTTCATGCC	CACCAAGCCATACCATTGC	lead	AS	2132319	2132334
upp(3.3E-3)	GAATCAACTGCTAAAATTGCTGAAC	CACCAGTCGCAAGCATTGG	lead	AS	1992330	1992345
yxbC(3.2E-3)	TCACGATGAAGTGCTTTTTCTTGAT	CTTCACCCGCTTCAATTTC	lag	AS	2316615	2316630
mae(3.2E-3)	AATCTACGTGCTGAACATTCGCACT	CAATAAACCTTTAAACAAG	lead	AS	1204442	1204457

feoB(3.1E-3)	GCCCCTTCAAGTCGATGGTCATAAT	CTAAAGTCTGACTTTCATG	lag	AS	191636	191651
yhfB(3.1E-3)	TCTTATATTTATCAAAGTCAGTAGC	CATAATCATTTTAATTAAA	lead	AS	752675	752690
uvrB(3.0E-3)	AAAATTGCCAAATGGTCTACTTCAC	CTAAAACTTGTCCGGTCAA	lead	AS	556835	556850
xerD(3.0E-3)	CATAGAACTCAGGAAATTCCTTAAT	CATTGGATGATAACTTTGA	lead	AS	1310409	1310424
intergenic(2.9E-3)	AATAAAAAAGCCCTGAAATCAGGGC	CTTTAAGATTTACATCATT	lag	.	672356	672371
pflA(2.9E-3)	TCATCAATTTTTTCGGTTACATATT	TATCAGTCAAATATGGTTG	lead	AS	1880878	1880893
ycjC(2.9E-3)	AATCGATAGTGAACACCTTCTCGGT	CATGTTCAAGTTCAATGTC	lead	AS	292573	292588
citD(2.9E-3)	GCTTACCAAACTGTTTTTTCACATC	TGAAATCAAATCAATCGAA	lead	AS	1207881	1207896
murA1(2.8E-3)	GTTGGACGAGGGCCTAAATCACAGC	CACCAGGCAAGCCTACTGT	lead	AS	313400	313415
pepXP(2.8E-3)	CCAGGAGAACGGACAAGACCATTTT	CACGATAGTAATTATACCA	lag	AS	2135812	2135827
citF(2.8E-3)	GGACTTTAGCACTCACGCGCTCAAT	CACAAGGTCCTTGACTTCG	lead	AS	1209068	1209083
ydgF(2.8E-3)	TATGCCCTAGATAATCATTCTTGTC	TAATGCAGCAAGCCCAATT	lag	AS	364624	364639
ycbC(2.8E-3)	ATAGCCGAAGTTGTATAGGCATTAT	CATTTCTCCTTTCCTAATA	lead	AS	212015	212030
ycbB(2.8E-3)	ATAGCCGAAGTTGTATAGGCATTAT	CATTTCTCCTTTCCTAATA	lead	AS	212015	212030
yffD(2.7E-3)	ACCTTGTGGGAAGCCCCAGCTCATC	CCATCTGCTCGTTTACCTA	lead	AS	555725	555740
citR(2.7E-3)	TTAGAATAATTGACCATTTCACTCC	CCAAGAACATTCTGACCTT	lead	S	1206297	1206312
noxD(2.7E-3)	AAACTATCTCTTTATTAGTCGTTTC	CACACAATCTACCTTTCAT	lead	AS	2194773	2194788
yvjA(2.7E-3)	AAACTATCTCTTTATTAGTCGTTTC	CACACAATCTACCTTTCAT	lead	AS	2194773	2194788
uvrA(2.6E-3)	CCATCAACACGAACACGAACATAAC	CATCTTTTTGGATACGCTC	lead	AS	1887923	1887938
yoaB(2.6E-3)	AAAGTTGTAAAGGCCATAGCCACAC	CCATTTCAGGTGATGTTTT	lead	AS	1403419	1403434
hslO(2.6E-3)	TCACCAGTAATTAAATCAACTTGAC	CACTATAAGGTTGTTTAAG	lag	AS	2034682	2034697
intergenic(2.6E-3)	CCAGCAAGAGCAGTTGCAACATAAC	CTACAACTTGTCCAAGAGC	lag	.	492284	492299
yngF(2.6E-3)	AATCCGTAAACAACACCACCAAGAC	CAGCAAGGGCACCAGCGAC	lag	AS	1367812	1367827
intergenic(2.6E-3)	CAAAGATAAGCGAGATAATCGTTGT	CCAAAGTTCAGGGTCTGGA	lag	.	492237	492252
yahG(2.6E-3)	TTTTGTCAATACAAATTGACGCAAC	CAGTCAAGAATAGAGTCAC	lag	AS	73624	73639
citE(2.5E-3)	CTTCGATACCGGCTTTCACCATGGC	CCGCACATCTTTTTGCCAA	lead	AS	1208323	1208338
ysjA(2.5E-3)	GCCTGTGCAATGACTTTTCCAGAAC	CTAAAAATAGTCCTGAACC	lead	AS	1891336	1891351
mutS(2.5E-3)	CTGCCCCAAGTTCATCAAACAAGAC	CAAACTATTTTCATCTGCT	lead	AS	1692844	1692859
rgrB(2.5E-3)	TTGGTGATATTTAGTTTTTCACATT	CAACCTCATTTGGCGCCGT	lead	AS	1461344	1461359
L200146(2.4E-3)	CCTTCCAGCACCGGGCAGGCGTCAC	CCCCTATACATCACCTTGC	lead	AS	1982423	1982438
citC(2.4E-3)	ATGGATTAGCATTCATGACCACTGC	CGCGATTTTTTTCGCACCA	lead	AS	1207184	1207199
yrbB(2.4E-3)	TCGTGAGTAATGATATAAGCCCATT	CGCTAGCAGAATAGCCCGC	lead	AS	1711588	1711603
clpE(2.4E-3)	ACAATTTTAAGGAGATTTTCTTTGC	TCAAAGCAGAGAATTCAAT	lead	AS	552319	552334
citD(2.4E-3)	CTACCGCAATCGCTCTGGCCTTAAT	CACCAAATCCAAAGCCCCT	lead	AS	1207998	1208013
citF(2.4E-3)	TCACATCAGGCACAACTGTCGCAAT	CCGACCGCGCACCAAAGGT	lead	AS	1210229	1210244
ypbD(2.3E-3)	CATGCTAAAATACCAATGCCAAAAC	CAATTAGGTAAAGCAGGCG	lead	AS	1514931	1514946
ycgD(2.3E-3)	GGTTGACAATTCGTTTAATCACAAT	ATTCTCACGTTCTTTCACG	lag	AS	264608	264623
nrdG(2.3E-3)	AGTGAGCAGCGCACCCCTTCACCAT	CAACGAAATTGAAGGGTTT	lead	AS	272739	272754
gidC(2.3E-3)	CCAGAAGCAGCTGATTCTACATAGC	CCTCTACTCCTGTCATTTG	lead	AS	1257813	1257828
citF(2.3E-3)	CGACACCTTTTTCAATAGCCTCAAC	CAAAATATCATTCATGACA	lead	AS	1209302	1209317
pfl(2.2E-3)	GGCCCAGCAAGAAAGCTTTCATCAC	CATCATATGGTTTGTAGTT	lead	AS	657523	657538
pepDB(2.2E-3)	CGTAAATCAACAAATAAATCATAAT	TAGAATCACCAAGAACAGC	lead	AS	1600812	1600827
yneH(2.2E-3)	TCTGACAATATCTTCTTTACACAAT	GAAGTTTTGGAAATGACAA	lead	AS	1346280	1346295
yfcI(2.2E-3)	AGTGCTGACGAATCACTTCCAGGAT	CAATAATGAGATGTGAAGT	lead	S	529084	529099
potA(2.2E-3)	GTTGAATGAATGAGCCATTCGTTAT	TAAATTCATCGTAGGCTAC	lead	AS	1176611	1176626
lcnC(2.2E-3)	TTAGGCGCTAGTGAAGCTACTTCAC	AAGAAGAGATTATTAGAGC	lag	S	84331	84346
sdaB(2.2E-3)	CACCGATTGAAATTCCAGTCACGGT	CATTGTTCGATCCCCTTTT	lead	AS	843781	843796
yjhD(2.2E-3)	ATTGATTTAATCAAAATTTCATCAC	CTTTTTGATAGCCTTTTTG	lead	AS	973074	973089
ygdA(2.2E-3)	CGTAGAAATCATGTCCCAACATATC	CATCTGAAGCACTGCTTCT	lead	AS	632818	632833
intergenic(2.1E-3)	GACAACAAAAAAAGCCGGCCATCTC	CCCAGATAAACGACTTTAA	lag	.	506679	506694
yyaL(2.1E-3)	ACGTGAATGATGGCATCTACTTCAC	GAATATTGGCAAGAAATTT	lead	AS	11386	11401
hflX(2.1E-3)	TTGCACCAAGGCACTTTTTCGTAAT	TCAGGTAACTGATAGGCTT	lead	AS	225887	225902
citF(2.1E-3)	GCGCCACGAATGACACCGTCAGAAC	CAGACATAACGTTAACGTT	lead	AS	1210134	1210149
glnQ(2.1E-3)	TTTTGCATAACGGCTAGAACGTCAC	CAACCATTTCAGGGTCAAG	lag	AS	1819568	1819583
yoaD(2.1E-3)	TGTGCTTTAGCCGCGAAAATACATC	CGCAATAACATTGACGATA	lead	AS	1406099	1406114
ywiA(2.0E-3)	TCTTGCGCAAATTCACGAACATCTC	CAATATTACCTGGATTTAC	lead	AS	2280342	2280357
pepO(2.0E-3)	AACCAATGAAAGGAGTAATAGCATC	CAATTTTTCAATGGCTTTT	lag	AS	1867496	1867511
citC(2.0E-3)	GTCGCTGAAATGATTTCTTCATTAT	CATCAGCTTTTTTTCGCTC	lead	AS	1207623	1207638
potB(2.0E-3)	AAAATGATTGCCATAAAAGCATGGC	CAAAGGGACGATAACAAAG	lead	AS	1177014	1177029
ylhB(2.0E-3)	CAATAATAATTTTAGCCGGCATAAT	CATTGGAATTAAAATTCCA	lead	AS	1173553	1173568
intergenic(2.0E-3)	TAGTAATAAATAAACAAGGCTTGCT	CATTATAGTGCAAAAAAAT	lag	.	494197	494212
pnpA(2.0E-3)	AGCGCTCCGTGACCAATTTCACGAC	GTCCAGGTGCCCCGTAACG	lead	AS	1923022	1923037
ylhB(1.9E-3)	TCAGGATGTTTCTTTAGGACATAAG	CACCCACTGTCATTACAGG	lead	AS	1173752	1173767
intergenic(1.9E-3)	TACTGATTATTATGATAAACATAAC	TATTTTAAAATAGTTAAAA	lag	.	2316411	2316426
ynbC(1.9E-3)	GTAAGAAAATCATGATATCCACTAT	CATAATCACAGTTCCCAGC	lead	AS	1311522	1311537
intergenic(1.9E-3)	TTGCGAAAAACAAATCGTTCTCGAC	TTTCAAATCAGCCCATTCG	lead	.	1501790	1501805
ytgG(1.9E-3)	TTACCATCATCAATATGTACTCGAT	CATAAAAAGCTGTCTTTAA	lead	AS	1969437	1969452
tra904E(1.9E-3)	CCAAAGAAGGCACCTTATACTTAGC	CGTGAATATCGACGTTTTT	lead	S	626963	626978
citF(1.9E-3)	TAAGGCGAATTCACAATAACATCAT	TAACCATAGAGGCAATTTT	lead	AS	1209756	1209771
yoaB(1.9E-3)	CTCGTTTAGTTTGCACGACCGCAAC	CACTGAATTAATCATCAAA	lead	AS	1405483	1405498
ygfC(1.9E-3)	ACTTGTGAAATTGGATTATCATTTT	CACTAGCTGGGACGGTTGC	lag	AS	653935	653950
pacL(1.8E-3)	GATCCCCTGTAATCATAACCACATT	AATATTTGCATCATGAAGT	lead	AS	677838	677853
pepDA(1.8E-3)	AGACGATTAGGAGCAATAACATAAG	CGTCGTCGGGGATACGAAT	lag	AS	249255	249270
ydiA(1.8E-3)	GAGAAGTCTATGATGTATTCAAAAT	TCATATCATTTTTAAAACA	lag	S	381887	381902
yrfE(1.8E-3)	GTTTATACTTTTTTTCATTCATTGT	CATATTATTATATCAAAGT	lead	AS	1760358	1760373
potA(1.8E-3)	CTCAATTGGTTCATTTTTACGCATC	CCACCATCGACAGCTTCAA	lead	AS	1176465	1176480
yahA(1.8E-3)	AAGAACCAGCGTATTCAAACATTTC	CAAATCATTCATTTGGTCA	lag	AS	72409	72424
glnP(1.8E-3)	GGGAGATTGATGGGAAGTTCATTGC	TAAATCTTGCCCTTGTTTC	lag	AS	1817470	1817485
ptnC(1.8E-3)	GAAGATGCAGTACCATTTCCAGAAC	CACCAGAAGCTTGGAGGTT	lag	AS	1764021	1764036
malG(1.8E-3)	TAGATTTAAGAATTGAATACATTGC	AATCATTCCCATGATTCCT	lag	AS	1742084	1742099
yudI(1.8E-3)	CCGCAGTGACGCCTGTTTCATCAAT	CATGCGTTTCGCGTCTTCA	lag	AS	2036542	2036557
yljG(1.8E-3)	GCCAAAAGATTTCCATTAGCACAAC	CAACATCTAAGATACTTGA	lead	AS	1197300	1197315
pepQ(1.8E-3)	TTACGTTCAGCAGCAGTGGCAAAAC	CAATTTCAAAACACTTGTC	lag	AS	1697596	1697611
frdC(1.7E-3)	ACCAAATAACCACCAACTGCTGAAC	CATCATGTGGTCGGTGAGT	lead	AS	1138403	1138418
pepQ(1.7E-3)	AAAGACAGTCTTCAATACGCACACC	TACTTTACCAGGAATATAA	lag	AS	1698129	1698144
yqjD(1.7E-3)	TTGCTACTCAAACCATTAGCATTAC	TACTTGTGCTGGCATTTGT	lead	AS	1698715	1698730
ywcC(1.7E-3)	GTTGTCAAAGAATCATGTCCACGAT	TAGAAGTATAGAGGAATTT	lag	AS	2229225	2229240
clsB(1.7E-3)	CCTTATTAAGCCCTATATACAAGAC	CATTTATAATATCGGACTA	lead	AS	1188583	1188598
deoB(1.7E-3)	AATGGTGACAAAGCATAGTCACGAC	GTCCATCAGTCCGTTCAAA	lead	AS	955858	955873
dexA(1.7E-3)	CCTTGCAAATCTCCAACACCATCAC	CATTAGCATCCATAAAAGA	lead	AS	1736621	1736636

glnP(1.7E-3)	AAATAACTGGAACTTCGTCCATTGC	TCCAGCAATATTACCAGAG	lag	AS	1817416	1817431
ptnC(1.6E-3)	AGGAAGAAGAATGGCCATACTTCAC	GAGTAGCCATAAGGTTGAT	lag	AS	1763895	1763910
glnP(1.6E-3)	CCTGACATCATTCCGTCAGCATGTC	CAGATTGAACAGAGTCAAC	lag	AS	1817828	1817843
citG(1.6E-3)	GATGCTCTTCAAAATCAATCATAAT	CGTCTTTATCATTTTCAAA	lead	AS	1210864	1210879
mae(1.6E-3)	GAACAGCTGTTCCATCAGTCACAAC	GGCCACCAGTTTTCCGCTC	lead	AS	1203656	1203671
ycgD(1.6E-3)	AATCACCCAAATAACCTTGCTCAAT	CACTTTTTTGACGGTTAAA	lag	AS	264275	264290
ybaA(1.5E-3)	GATGCAGTTTTATTTTATAATCCCG	CTAAATCACGTTTTGATGA	lag	S	101429	101444
pbuX(1.5E-3)	GTGTGGCAACCATACCAAACATAAT	CAACATCGCTCCACCCAAT	lead	AS	1161385	1161400
clpP(1.5E-3)	CGTGAATAAATATCATAAGCACGTT	CGCCACGGCTTGATTGTTC	lag	AS	672927	672942
rliB(1.5E-3)	TTTCCTCGTGTTCTACTGACAAGAC	CAAGCTGTTCTAATTCAGT	lag	AS	1535352	1535367
L200097(1.5E-3)	TTTGATTAAAAGTCAAATGCTCTAC	CAACTGAGCTAACGAGTCA	lead	AS	542710	542725
yoaB(1.5E-3)	CTTCAGGAATTGCAGCAACCGCAAC	CGCAACTGCAAACATGAAT	lead	AS	1404904	1404919
gatB(1.5E-3)	CTATTCAAATATTGAGCCACTTCAC	CTTGCAACCAGTTTGACAC	lead	AS	168767	168782
uvrC(1.5E-3)	CTTGACCAGCTCCACCATCCATTGC	AATCAAATCAGGAAGAGCC	lead	AS	857972	857987
intergenic(1.5E-3)	TGTTGTAGTTTATTACCTACTTCAC	TTCTATTCTAATAGATATT	lead	.	1299735	1299750
pycA(1.5E-3)	TCGTTACCACGCGCATAAACATCTT	CTTCTGTTAAATCATTTTG	lead	AS	666290	666305
rmaA(1.5E-3)	CTCTGCTAACGTATTCTTTTTCAAT	CAGGCCTTTAATTGCTCGA	lag	AS	749971	749986
ybdC(1.4E-3)	CAGCAGCAAAGTTAACCCCCATAAT	CAAGATGAAAATAGGCATG	lead	AS	132449	132464
ywaH(1.4E-3)	GTAAGCATTACTTCGCCTTCACGTT	CAAAATTATAAGTTTCTTT	lag	AS	2208331	2208346
yahG(1.4E-3)	TAATCATTTCTCCTTCAATCGCAAC	TGAAAGATTTTCTACACGA	lag	AS	73866	73881
gidC(1.4E-3)	TTGTACAAAGATGCGGAAGCATTAT	CTTGGCGAAGTTGAACAAC	lead	AS	1257582	1257597
malF(1.4E-3)	GTTGGAATATGCTTATTGTCACGAT	TAAGTGCATAGAGGTGACG	lag	AS	1740735	1740750
als(1.4E-3)	CTCCTTGCTCATGACGAGTCACGAC	CATTTGAGGGCCTTCTTCA	lead	AS	1200572	1200587
feoB(1.4E-3)	CGGAGAGAATAATCATGGACATTCC	CAAAAAGTAAGCACTAGGA	lag	AS	190731	190746
rliB(1.4E-3)	TTTTCTTTAAGGTAACTTTCGTAAG	TGATAAATACGGTTGTGTT	lag	AS	1535526	1535541
pbp1B(1.4E-3)	TTGCCGAAACTTTATCTCGCTTAAT	CAAATAATTATAGATATCA	lead	AS	392472	392487
fabG(1.3E-3)	GTCGTATAAGCGACGCCACCACCAC	CGGCAATCAAGCCAGCAAC	lead	AS	801923	801938
yxaB(1.3E-3)	TTTGTATTAGTTTCATTGACGTAAT	CATGAGCGTACTGTTCAAT	lag	AS	2301202	2301217
yvhB(1.3E-3)	ACTCGCGAAGGAGCGAATCCGTCAC	CAAATAGCCCGAGAGAACA	lead	AS	2176801	2176816
rlrD(1.3E-3)	TGAATCATAGGGTCTTTTTCTTCAT	CAAAAGAAACTCCTAGAAA	lag	AS	380962	380977
yngF(1.3E-3)	ATGTATCCAAATCCGTAAACAACAC	CACCAAGACCAGCAAGGGC	lag	AS	1367821	1367836
glpD(1.3E-3)	ACCTTGATAGCATCTACTCCTTCAC	GCATAAACAAGATGTGGTT	lead	AS	1270237	1270252
ysgC(1.3E-3)	AATGTATTATAGCGAGCCACTTCAC	GCGCAATTGCCGCCGGAAT	lead	AS	1870118	1870133
yscB(1.3E-3)	AGGAAAATAGTCTCTCATCCATAAC	CATTGGGAGCCTATACGTA	lead	AS	1822184	1822199
cysM(1.3E-3)	GTTGATAAGTAGCGTTCTCCATTAT	CAGGAGCAATCGCTAACAC	lag	AS	526318	526333
intergenic(1.2E-3)	TTGAGCCACAGATTCTCTCCATTTC	CTAGATTAATAATCTAAAA	lag	.	1765153	1765168
yijC(1.2E-3)	GAACCTCTGCCCATTCCTCCTTCAC	GTACACCAAATCCACCAGC	lead	AS	893748	893763
ynhD(1.2E-3)	AGAGAACTAATCCAAAAATCACATT	CACGATTCCTAGATGACCA	lead	AS	1376527	1376542
intergenic(1.2E-3)	ATCTATAAGAATTTTCTTCGACAAT	CATGAGCCTTTTCAAAATT	lead	.	600720	600735
citD(1.2E-3)	CTGGCGCAATCATGATTTGCACATC	TGAAGACTCCAAAGTCCCC	lead	AS	1207824	1207839
yudI(1.2E-3)	ACCACATCATAAACTTTATCAGGAT	CAAGCAAAAGACGAGAACC	lag	AS	2036249	2036264
codY(1.2E-3)	GAAGCGATAACATGCCCTTCTTCAC	CATCAAGCTCTTCAATGAT	lead	AS	164615	164630
hemH(1.2E-3)	TGCAGATTGTTTAGAAACTTTATTT	GAATTAGAAATTGAAAATA	lag	S	1608898	1608913
yngF(1.2E-3)	TATTGAAGAGACCTACTTTCATTGC	TACCGCAAAAGATAAAGCG	lag	AS	1367256	1367271
yuhE(1.2E-3)	AGTGCGTCAGTATCTATTTCATTGC	CAGGAGTAAGGGCACCAAA	lag	AS	2073300	2073315
yudH(1.1E-3)	GACGGAAAAAGTGAAATTCCGTCTC	CTAGTAAAACTGGGGCAAT	lag	AS	2035630	2035645
intergenic(1.1E-3)	CCTCCCGAAGGAGAATTCTCTTTTC	CCTATTTGAATCAGATATT	lead	.	1641659	1641674
mapA(1.1E-3)	GTTTCACTAAAATTTCCGCGCATTC	CCATGTAGCCATTGCCAAT	lead	AS	1731080	1731095
pbuX(1.0E-3)	AATGAACCAAACATTGCTCCAGAGC	CATGTCTTTGACCAATAAT	lead	AS	1160642	1160657
maeP(1.0E-3)	AAATGATAAATCTTCCTGCCAAAAC	CACAATCGTATGAAAATCA	lead	AS	1205444	1205459
menB(1.0E-3)	TTCAAACGAGGAATTTGGTCTTCAC	CAACATATCCACCATTTCC	lag	AS	734925	734940
intergenic(1.0E-3)	ATGCTTAAAGCGTCATTTTCACGAC	GAAATATATTATAACACAG	lead	.	618453	618468
yrjE(1.0E-3)	ATTGCCCAACCATCACTTACTTGTC	CATTGTTTGCATTATAAAC	lead	AS	1794110	1794125
menF(1.0E-3)	TTAATTCGGTTGTTCCAACCGTTAC	TGTCTCGGCTTTTTCAAGC	lag	AS	738516	738531
ycgF(1.0E-3)	TGTGCTTTACCTAAGCCTTCTTTTC	CAATGATGGAGAAAGATGA	lead	AS	265738	265753
ythB(1.0E-3)	AACTTCCGAAAGTACCAAGCACAAT	CAGAACCCGAATTACATTA	lead	AS	1974386	1974401
pepXP(1.0E-3)	GCAGCAAGCACATCTAAATCTTCAC	CAGGAAAGCCACCAGGAGA	lag	AS	2135848	2135863
yuhI(1.0E-3)	ATATCAAGGAAGGCAAGAACATCAT	CATCTTCATAAATTTTGGT	lag	AS	2077617	2077632
clpC(1.0E-3)	AAACGGTCAATTTCAGCTTCTCGTC	CAATCATTGGGTCAAGTTT	lead	AS	630372	630387
menB(1.0E-3)	GGAATTTGGTCTTCACCAACATATC	CACCATTTCCACGTACTTT	lag	AS	734934	734949
intergenic(997.7E-6)	AGCAGATTTTATTCGCTTTCATTTC	ACAATGTCTATTATATCAC	lead	.	779365	779380
ppiA(993.9E-6)	TCATATCTTGGGAATTTTGCACAAT	GAAAAATTGTGAACTGCTA	lag	AS	368737	368752
pstA(988.2E-6)	ATCTTTCACTCCTTTTAAACGCAAT	CCATAAACCACATTTTCAT	lead	AS	1771561	1771576
intergenic(985.4E-6)	AGTTGACCAAATTCGACTTCGCATG	TAAAAAATAATACTTTTTC	lag	.	461287	461302
aroH(983.5E-6)	ATGACTAAGAGGATTCTATCGTCTT	CACCCTTAATTATCGCTTC	lead	AS	1281265	1281280
pbp2A(983.5E-6)	TTAGGTGAAGCTTTCCAAGCAGAAC	CATTAGTATAAGTCCCCAG	lead	AS	2177528	2177543
yfiI(972.1E-6)	AAAGAGACTCTATCTTTTCCGCAAC	TACCCAAACATCTCCATTT	lead	AS	587196	587211
intergenic(964.5E-6)	AGTCCAAAAGACATCCATTCGTCTT	CTCCTTTATTATATAAATT	lead	-.	1204656	1204671
ykiF(960.7E-6)	TCTAAATCAAGCCAATCTTCAGAAC	TCAAAGTTTCTTTGATAAA	lag	AS	1087045	1087060
ysiC(957.0E-6)	CGATTGATTAGAAGAGTTGCATAAC	CATGACCTCTTGCTTCTTT	lag	AS	1884434	1884449
yudI(956.0E-6)	ATTCGTTGTAAATTTTGTCCATAGT	CATAAAGAATATTATAGCA	lag	AS	2035870	2035885
dacA(954.1E-6)	TTTTGAGTTAATTCATAAGCATAAT	CTGAAATAGGAACTTTAGT	lag	AS	2356058	2356073
ysjA(942.7E-6)	AACTCAGCAATTTATGCCACTTCAC	GCTCACTCTATTCACGCAT	lag	S	1890475	1890490
L200091(932.3E-6)	GCAGAGCCATGTGTTACCACTTCAC	TACGCCGCCACAACATTAA	lead	AS	424310	424325
ycdJ(925.7E-6)	TTAGAGGCAAGTTCACCAACTTCAC	AAGCAAGCTTAGCTCCAAG	lag	S	237900	237915
yogG(916.2E-6)	CTTGATTGTCTTCATAATACATATT	TGTTTTAACCATTAATTTT	lead	AS	1466341	1466356
intergenic(915.3E-6)	TTGCGCAAGACGCAAAAGGCTCAAC	TATGCTAGTGTCTTTGTCA	lead	.	1815757	1815772
Intergenic(901.1E-6)	ATAAAATTGATTAATATTACGTAAT	CTTTTAGGAAATAATGCTA	lead	.	1516165	1516180
pspA(868.8E-6)	CCATTATATAATGCCACTACTTCAC	AGGCTTGATAAGCTCCGAT	lag	AS	2304622	2304637
ponA(867.9E-6)	TAAGCAGCAGCCATTTTTTCAGAAC	TTGCTCCACCATTTTTAGA	lag	AS	529759	529774
ycgE(844.2E-6)	CTGAACTATGTTCACCCCACATAAT	CATGTGGAAACCAGTTTGA	lag	AS	265242	265257
nrdF(837.6E-6)	TCATCAGACATGGCTTCCACTTCAC	CAAGAAGGTATGAATTACC	lag	AS	1001348	1001363
citC(837.6E-6)	GCGTAAATGGATTAGCATTCATGAC	CACTGCCGCGATTTTTTTC	lead	AS	1207190	1207205
ykhD(830.0E-6)	AGCACTTTCGTTTCATAGCCGTATC	CACGTCGGCCTAATTCGCC	lag	AS	1072513	1072528
pheS(821.5E-6)	GTTTTACTTCGTACGCATACTTCAC	CTATGCAAGCTCGGACAAT	lag	S	2009589	2009604
intergenic(817.7E-6)	TCGCTTAGTCTGTAAGTTGCGTAAG	CAAGCAGATAGGAGTGATA	lag	.	2281324	2281339
yxbD(811.0E-6)	GCAGGTCACGTTGTCCCTGCTTCAC	GTCGTGGTGAAGGAATTGG	lag	S	2319277	2319292
mscL(809.2E-6)	CCAACGGCTAAGTCCAATACGTTCC	CACGTAAAATAAAGTTTTT	lag	AS	2170604	2170619
yhcA(803.5E-6)	GTTGGCTCAACTTTTTTGACGTTAT	CAACTTTTTTTCAATTTTGA	lag	AS	720821	720836
L200142(781.7E-6)	TTTACGATCCGAAAACCTTCTTCAC	TCACGCGGCGTTGCTCGGT	lead	AS	537959	537974

clpE(771.3E-6)	GTCAATTGACCAGCTTCTTCATAAC	CCACATATCCTGGAGGGGC	lead	AS	551980	551995
queA(768.4E-6)	CCAGCAGTTGGAGCAGCAGCAGAAC	CATTCTCTTTTGCAAAAAC	lag	AS	1617278	1617293
yafC(759.9E-6)	ACAAGTTCAGCCTCATTACCATAAC	CATGTTCTGACTGACTAAA	lead	AS	55665	55680
phoL(756.1E-6)	ACAAAAGTCCCAAACTATTCATCAC	CATAGGCTTTGATAATTTC	lead	AS	1094072	1094087
intergenic(735.2E-6)	GGTAACTACGGCACCAATCTTAATC	CTCTGTGGCCCACTTTTGA	lag	.	1259597	1259612
rlrD(706.8E-6)	CTTTGATATGGGAAATAATCATCTC	ATTGCTATTCATACGAACA	lag	AS	381092	381107
intergenic(701.1E-6)	TATATAAGTCCCTGGGCTACTTCAC	TTTTGGTTGAAAGTAGCTC	lead	.	133745	133760
intergenic(694.5E-6)	ATCGTAAAACAAAAACTTACACTTT	CATGTTATCATAGTTAATA	lag	.	172797	172812
llrB(688.8E-6)	GATAAAAGGGTCAAAATTGCCCGAT	CATCATCAGCAATTAAAAT	lag	AS	1458185	1458200
tra904C(678.4E-6)	GAAGATAAAAGCTGTCTTTCATGAG	CATAAGGGACGCTATGGTG	lead	S	140716	140731
intergenic(678.4E-6)	CTTCTATAAATTAAACATACTCATC	TATTCTACCTAAAATTCAG	lag	.	565296	565311
intergenic(678.4E-6)	TTCTTAAACCATAACCAATCGTAAC	TACATCAAAATTTTCATTT	lead	.	1718258	1718273
trmB(674.6E-6)	GTGATGAAAGCTCCTTTTCCACAAC	CCACTTCAATATGAATCGG	lead	AS	756416	756431
dacA(669.9E-6)	ACGAGTTGAACAGATTTTTCTTTTC	CATCAATCACAGTAATTTC	lag	AS	2356769	2356784
aroK(662.3E-6)	GTTAAATCGGCCACTTTTGCATAGT	CATTTTTTCTTTTTTTCAAA	lead	AS	1801096	1801111
citF(633.9E-6)	GATTACCGACCGCATCAATCTTAAC	CACGTAATCGACTTGCGTT	lead	AS	1209659	1209674
pflA(626.3E-6)	ACGTGACGAACCCACATTGCATTAC	CACGGTCTGATAAATACTG	lead	AS	1880728	1880743
pnpA(606.4E-6)	GCTCGTTCACCGAGCGCTCCGTGAC	CAATTTCACGACGTCCAGG	lead	AS	1923010	1923025
pi234(605.4E-6)	TTCATCCATAATTTGCCTCCTTCAC	GATTTGAACATAGCGATCA	lead	AS	1058576	1058591
recO(604.5E-6)	ATATTTTTATAAACGACCACTTCAC	TGACATCCTCGATAAAAGA	lead	AS	58059	58074
pacL(604.5E-6)	AATTGAAGTTCATCAAGCTCTAACC	ATCTTATATCGGCCTCCAC	lead	AS	676631	676646
dxsB(598.8E-6)	GTCAAATCACGCGCTTTGGCATAAC	CTAAGGCATTAGCAATTGA	lead	AS	1724988	1725003
yahI(596.9E-6)	GCACCAAGTGGCATATAGACATCTC	CACCAATTGATGAAATATT	lag	AS	77909	77924
yciC(595.0E-6)	TGGCAGTTCCATTCCCAATCGCAAT	CATATTCACACCATATTCT	lead	AS	285581	285596
hom(593.1E-6)	TCTGCCAAAGCACGCAATTCAGTTC	CATGAACAGCCAGTAAATC	lead	AS	1171374	1171389
rnhA(593.1E-6)	AAACCGAGATAGAAGTTTTCCCAAC	CTTGGCAAAAAAGTGATG	lag	AS	2349530	2349545
pbp2B(591.2E-6)	GAAGGAGCAAAGGCCACTGCATTAT	TAGCTGTTACAATAACTTG	lead	AS	339692	339707
ythB(589.3E-6)	ACGGTGTCATCAATAGACTCGCAAT	CACATAAACAATGAGTAAT	lead	AS	1974335	1974350
intergenic(573.2E-6)	CCCTCTGGGTCAGCTCGTACTTCAC	GCCGTCACTGCACATATTT	lag	.	2016727	2016742
pfl(567.5E-6)	TTCAAATTCTTGATGGATACTTCAC	ACCAGGTGCTTTGATTAAT	lag	S	659540	659555
dukA(538.2E-6)	TAAATCGAGCGGTCTAATACATTAT	TATTATCAGACAAGGCTCT	lag	AS	493902	493917
rpoC(526.8E-6)	GAACGGTAAGTTTTTTGTACTTCAC	CGAGCAAGTAAGTTTCAAC	lead	AS	1857655	1857670
gidB(520.2E-6)	CCTCGAAAATGTGACTTTACTTCAC	GGACGAGCTGAAGATTTTG	lead	S	1381124	1381139
trpD(520.2E-6)	TCCACCCAGCGATGAAATACTTCAC	GCCAGTCCGCAGACAACTC	lag	S	1498901	1498916
gntR(519.2E-6)	CCCCGCGACTCTTCAAAATTTCAAC	CAAGTGCATTGTATCTCTG	lag	AS	2272093	2272108
intergenic(513.5E-6)	TTCTGGATTTAAGAAATTACGCAAT	TGAGATTATAACTTATTTA	lead	.	1327864	1327879
asnB(509.7E-6)	GTTCGTAAGAAATCTGCCACTTCAC	GAGCATATTTCAAATCAAT	lead	AS	356847	356862
glnP(504.1E-6)	CAAAGATAATTCCGACGATCATTGC	TAAGATAAATGAGAGAATG	lag	AS	1818448	1818463
yseI(493.6E-6)	AATTGATTTAGAAGTTCTTCTTTGC	CATAAAGAACGCCAATTCC	lead	AS	1849361	1849376
hrcA(488.0E-6)	ATAAATTGATTTGTTCTTACTTCAC	CAGTCCCTAATGTTATAAC	lead	AS	977222	977237
dut(485.1E-6)	CTTGATTTTCATCACCATCAGCGAC	CAAGAAAGGCATGAAGACT	lead	AS	181536	181551
noxB(484.2E-6)	AACTTTATGCGTGGTCATACTTCAC	GTCAAGGAAATGTACTTTG	lag	S	842595	842610
ypgC(483.2E-6)	ATCTGACGAATAATTGGTTCGTAAC	CTTGACTTTCAGAAAGGAA	lag	AS	1562864	1562879
purA(483.2E-6)	GGTGTTTGTAAAGCCTATACTTCAC	GTGTGGGTGATGGTCCATT	lag	S	2029214	2029229
preA(482.3E-6)	GCAATCGCTTCATCATTTACTTCAC	GAGCCAAGAGTAAAGGTAA	lead	AS	187014	187029
rpsH(475.6E-6)	TACCAGATGTAGGCAATTACTTCAC	CACCAACATTTGTTTCACG	lead	AS	2162238	2162253
yogJ(470.0E-6)	ACATCCCTGAAGATTTTTTCTCTTC	CATAACTCTCTCATTTCCT	lead	AS	1468954	1468969
busAA(464.3E-6)	TCTGCTGTAATAACTTTTGCACGAT	CTACATCTTCTACGAAAGT	lead	AS	1474868	1474883
udk(460.5E-6)	TGTGAACGCGTATGTTTTGCATAAT	CATAAATCGGAATATCAAC	lead	AS	1709603	1709618
dxsB(459.5E-6)	GCACCAGCCGCAGCCATTGCACCAC	CAAAAGTAATGGTAAATTG	lead	AS	1724352	1724367
yrgI(459.5E-6)	ATGGACTGGAAAAAATTTGCTCGAT	TGATACATTTGCAAAAGCA	lag	AS	1769762	1769777
yreB(451.9E-6)	CTTTCCATACTTCATTAACTGAATT	CATATTATTTTTAATTGGA	lag	AS	1744102	1744117
citE(447.2E-6)	CCCTTGACAAAGCCTTCTTCATCAT	CAACATTGCCAAAGACCGT	lead	AS	1208681	1208696
yudI(442.5E-6)	AGTGGATGAGGGACCCCTTCAAAAT	CCATCATTTCCAAAGTCTT	lag	AS	2036072	2036087
yljF(426.4E-6)	GTTGGTTTCACTCCTAAAGCATAAC	TAGGTTCAACTGTTCCCAT	lead	AS	1196804	1196819
yriD(417.8E-6)	TCACTTCGAGCAATTCCAACTTCAC	AACGTGAGGGTGGTTTGGC	lag	S	1773284	1773299
dnaA(415.0E-6)	TGCTGTCGATGTCTTTTTGAACATC	CATATCATTTTTCATCTTA	lead	AS	1668	1683
kdgR(410.3E-6)	TTTTGTCCAGGACGATATTCATTCC	TGAGAATTTTGCCTGTAAT	lag	AS	1680250	1680265
ysfD(409.3E-6)	GTTGGATTTGGAAAGTTAACATTCC	CACATTTCCAAAGCGATTT	lead	AS	1854261	1854276
pknB(408.4E-6)	TTTGGATAGACTGAGTAAACATAAC	CATCAGCTTGTTGATTCGT	lead	AS	1955328	1955343
rplM(406.5E-6)	AGTATATTACTCACACTCACTTCAC	CCAGGTGGATTGAAATCAG	lag	S	2346912	2346927
gpsA(405.5E-6)	TTTCCCCAGATACGAACTTCATGAC	CATTATCATTAAGAACTTG	lag	AS	1376845	1376860
citB(401.7E-6)	CCTGATAATCGTGTTGCTACTTCAC	TTAAATCAACATTAATCGT	lead	AS	669535	669550
yvdE(400.8E-6)	AGCCCAAGGCACTTTCTTGTTCAAC	ACTAATATGATGAGTTGGA	lag	AS	2137423	2137438
yhfD(398.9E-6)	TTCCCAAAAAATGCATTGCCAGAGC	CACCTTTAACAGGCGTCAT	lead	AS	755426	755441
intergenic(398.9E-6)	ATTTCGCAAATGCTTACCCCATTTT	CATTCATTGATGATACTTT	lag	.	1002525	1002540
pacL(389.4E-6)	GTAGAGCAGGAATAAAAATCACAAG	TAGATATACAGGTAATGTT	lead	AS	678810	678825
pspA(389.4E-6)	GATAAAATTTTGAGGGTTTCATAAT	GGGTTTCTCCATGAAGTTA	lag	AS	2303688	2303703
uppP(381.8E-6)	AAGGTAAATTCTGCGGCAACTTCAC	GACTTACACCAACTAATAA	lead	AS	2275479	2275494
thrA(375.2E-6)	ACTTTTCGGCCGAATCCAACTTCAC	GGTTCATCAGATATTTACT	lag	AS	747685	747700
ydiF(369.5E-6)	CTTCACCTTGCAAAACACTCATAAC	CCGACGAGGGAAAGAGAAA	lag	AS	388917	388932
intergenic(369.5E-6)	CATAAGTCCTCCATTTAGACATAAT	CTTTTATGATTTTGTTTCT	lead	.	1166149	1166164
ypjC(355.3E-6)	ATTCAACGACTGTCACAATCACAAT	CGTATTAAGATAAAGGCTG	lead	AS	1592521	1592536
ptnC(353.4E-6)	ATAAGGTTGATAACCATTGCATAAC	CAACGGCAACAACCATCCC	lag	AS	1763862	1763877
ymjE(348.7E-6)	CTGTATAGATTGTCAAAAGCATAAT	CACAACAACTTCAAAAATT	lead	AS	1297570	1297585
nth(345.8E-6)	CTTGGGCAGATAAAAATTGTCGCAAT	CAATAGTTGAAAAGGAGTT	lead	AS	1083938	1083953
llrB(344.9E-6)	AGCCCTGTCAGTTTCATTTCATCAT	CATCTCGAGCAGTTAAAAT	lag	AS	1458407	1458422
asnB(341.1E-6)	GTTTCTAAGTGCCAAATCACTTCAC	GGACTGCTGAAAGTACATC	lead	AS	356922	356937
ydiF(341.1E-6)	TTTCATCTCGTGTTGTTTCCATTGC	CGCCTTTGTTTTCTTGAAA	lag	AS	387696	387711
intergenic(339.2E-6)	AGAAAGTTGTTATTGTCAAAAGGCC	CTTTTTGTTGTAAAATTGA	lag	.	1816604	1816619
ydiB(338.3E-6)	TTTGCTAGCCCTCCTTACACTTCAC	CAGGAGCTTTGAAGAAAGC	lag	S	383788	383803
ps123(332.6E-6)	TTGTGGTAGCTGGCAGGCTCTCAAC	CATTTTATTAAGAATGGCC	lead	AS	49501	49516
ysjD(330.7E-6)	TTTTTTACCTTGTCAGCCACTTCAC	CTGCTTCACCAGATAATCC	lead	AS	1893720	1893735
yrjD(326.9E-6)	TTTCTTTACCAATTTTCCACTTCAC	CAATTGGTCTGAAATAACA	lag	AS	1793542	1793557
fbaA(326.9E-6)	ACACAATGTGCCAATCGTACTTCAC	GGTGGTTCTGGTATCCCTG	lag	S	1979644	1979659
ptsI(325.0E-6)	GTAGATGGATCAAGAATTACTTCAC	CAGTCAATCCTGAAACAGC	lead	AS	121312	121327
pcrA(313.6E-6)	GACATCGAGATCCCACTGGCAAAAC	CACCAGATTTATAAGAAGC	lead	AS	1135794	1135809
yudK(312.7E-6)	TCAGTTCCTGAATAGGCTCCTTCAC	CGTGTAAATATGTTGCGCT	lead	AS	2038474	2038489
citG(307.9E-6)	CGTCCACAATTTTATTCTGCATTTC	CTACAATCCTATTCATTGC	lead	AS	1211075	1211090
intergenic(305.1E-6)	ACTCGCTATAGCTTATTTACGTAAC	CATGACTTTTAGGTAAACG	lead	.	1838537	1838552

yeaC(304.1E-6)	TGGAGATCATCCTCATTTACTTCAC	GCACAATCGTGTGGTGAAT	lead	AS	405707	405722
yfbJ(304.1E-6)	AATAGTCTATATAATTCCACTTCAC	ATTGGCATAAAGTAGATAA	lag	S	515575	515590
fbaA(304.1E-6)	TCACCACCAATTGAACCAACTTCAC	ATTCAACTGAGATGCCTTT	lead	AS	1979895	1979910
ps105(302.2E-6)	AAGGGCTTCACTCCCTCATCTTCAC	ACCATGCACGATATAAGGC	lag	AS	37893	37908
pcrA(297.5E-6)	GATTGATTGGTCAGCATCCCCAACG	ACACAGATATTTTGAAAAC	lead	AS	1134512	1134527
L200150(296.6E-6)	TACGGCTACCTTGTTACGACTTCAC	CCCAGTCATCGGTCTTACC	lead	AS	2221669	2221684
intergenic(294.7E-6)	GCACGTCATAGAGAGTTTTCATTAC	TACATTTTACCGTATTTGA	lag	.	2100424	2100439
intergenic(291.8E-6)	AAGGATAATATTTCTTGTTCACAAC	CTTGCTAGCCTCTCTGGAC	lead	.	1401689	1401704
recD(290.9E-6)	TAGGTCTTTCGATTCGCTCCTTCAC	GCGCCACCGCTTGTGCAAA	lead	AS	1797401	1797416
intergenic(289.9E-6)	TTTGTTCTAATTCATATTTCATAAT	CTCTTGGATTACTTCACAA	lead	.	1200392	1200407
ypgC(287.1E-6)	TAGTTTGGGAATAGTTTGTCGCTGC	CATTACACTTCTCCTGATT	lag	AS	1562527	1562542
ptnAB(287.1E-6)	CTTTATCCACCCCGGCAGACGCATC	CATCATGCGTTCTGTGTAG	lag	AS	1762532	1762547
yrgI(287.1E-6)	GGCCAAAAATCAGCTATTTCATGGC	CAACATTTCTTTCTCTAAA	lag	AS	1769877	1769892
yejH(286.1E-6)	TTATAAAAAGTTTTACTTCCACGTC	CACGTAAAAGAGTATTCAA	lead	AS	499252	499267
ylaD(286.1E-6)	AGACGTTTTGGAATATCTTCTCGCC	CATGATTCCCCGCTTCAAA	lead	AS	1105621	1105636
scrK(283.3E-6)	GCGGAAAATCATTGGTTTACTTCAC	AATTGGAACAGGAATTGGT	lag	S	1518450	1518465
fhs(279.5E-6)	ACAGTGACATGACGAAGGGCACGGT	CATTCAAATCAACAACACG	lead	AS	960252	960267
topA(273.8E-6)	AAAGCATTAATTTCATTCTCGCGGT	CAACAATTAGTTTTAAGGC	lead	AS	1255147	1255162
ymjE(271.9E-6)	GGATAGCAGAATTAACAATCATAAT	CAGTGTATAAATTACAATG	lead	AS	1296421	1296436
comFA(271.0E-6)	TCAACAGTGAGCACCCAAACTTCAC	TAAATCCGCACTGATACAA	lead	S	1097125	1097140
fabD(269.1E-6)	GCATAATCAACCGCCTCAACTTCAC	CACCAATAACAATTTGTGC	lead	AS	783367	783382
intergenic(268.1E-6)	AAAGTCGGCAGTCCAATTCCTTTTC	TATACAATTTGTGCTATCA	lead	.	498594	498609
ytbB(268.1E-6)	TCTTGAATGTTTGTAAATTCATAAT	CAGCAATCGTTTCTTTTTC	lead	AS	1913605	1913620
sdaB(262.5E-6)	GTGTATCAACTTCTAAAATCATAAT	CGCTTTTTCACCCTTAGCT	lead	AS	843991	844006
leuS(254.9E-6)	TCAACAACATCATCAGGATTAACAA	CATTTTTAAGAGATTTAGA	lead	AS	829278	829293
ilvD(252.0E-6)	TCGATGCTGTCAGCAATCACTTCAC	GACTGACCAAAGAATATCT	lead	AS	1246083	1246098
nrdD(251.1E-6)	TCATAGTTTGGTGTCCCTACTTCGA	GGTTAAGACCACGTTTGAG	lead	AS	271457	271472
citD(245.4E-6)	CAATCGCACGTTCCACTACCGCAAT	CGCTCTGGCCTTAATCACC	lead	AS	1208013	1208028
rplC(245.4E-6)	ACAGTGATTTCTGCTCCTACTTCAG	CGCCTTCGAGTCCTTTGAA	lead	AS	2169681	2169696
yfjF(236.9E-6)	CTTCAAAGTGACTTACATACTTCAC	TAGACATGATTCAGTGGGC	lead	S	599610	599625
yrjE(236.9E-6)	CGATAAGCCCTGCGAAAATCACAAC	ACCTACTAGGCTAATAAGG	lead	AS	1794180	1794195
clpC(233.1E-6)	ACACTTGCTTTATGCCCTACTTCAA	GTAGAAGATGGATTTGGTC	lag	S	630146	630161
ymjE(231.2E-6)	TCTTGACGATTAGTTTTTACCCAAT	CATTTTTAGAGTTGAAATA	lead	AS	1296165	1296180
relA(227.4E-6)	TTTTGTTTGGCTCTCTCAACTTCAC	GACGTTCATTTTCAGTTAA	lead	AS	108865	108880
ssbB(226.4E-6)	GAAGTTCCGCCTCCCATACCTTCAC	GAGCAGATCTACTTTCCAA	lead	AS	2274356	2274371
yeiF(225.5E-6)	TATTTGTACCTCGCACGTTCTCCAC	CAATGAGTTTAGGACGTGA	lead	AS	486830	486845
gyrA(222.7E-6)	AAGCCGCGAAATATCTCTTCTTCAC	AACACGTAACGGAATTGTT	lag	S	1123616	1123631
llrG(222.7E-6)	ATTTTTTCCTTGGTCACCACTTCAC	CATTTCCACGAAAAAGAAG	lead	AS	1805025	1805040
floL(221.7E-6)	GCTTCGGCCATTGCTGTTACTTCAC	GTTGTTTAGCAGTGGCTTT	lead	AS	746840	746855
ylaD(221.7E-6)	CCAATTTGAACTGCAACTCCTTCAC	GACCAACACTGGCACCAAA	lead	AS	1105705	1105720
L200115(220.8E-6)	GTCTGACAATGGAGCCTAGCGGGAT	CGAACCGCTGACCTCCTGC	lead	AS	2344208	2344223
parC(217.0E-6)	TCAGGTTACATCAAACGTACTTCAC	CACGTTCATTTGCTTCCTC	lag	S	1012443	1012458
rmaJ(215.1E-6)	AATTGCGAGAGAAGTAAGTCATAAT	TAATATTCTCGTTGCAAGA	lag	AS	583465	583480
leuA(211.3E-6)	ATTCATGTTTTCATTGCCACTTCAC	CTATTCACATGAAATATAA	lag	S	1240133	1240148
rpsM(200.9E-6)	CGTTTGATGTTAAGTGACACTTCAC	GACGAAGGTCTCCTTCGAG	lead	AS	2153631	2153646
intergenic(199.9E-6)	CATTTTACTGAAGTGCTTTCATTTC	TATTGTAATTATAACTGAT	lag	.	1712489	1712504
ponA(199.0E-6)	AATCCATATACTAACCCTACTTCTG	CTAAATGGCGCCGTGATAT	lead	S	530491	530506
yieH(196.1E-6)	GCGACATACTGATGATATGCATCCC	CAGCCAAAATTGACCGTGA	lag	AS	850840	850855
purL(188.5E-6)	ATATCCACAACCCCAGCTCCTTCAC	CAGGCCCCATCAAGACTTG	lead	AS	1576156	1576171
intergenic(186.7E-6)	AAAAAGTTCTTTAAAATAGATTTCT	TCATATCATTTTTATTTAC	lead	.	1677069	1677084
L200065(185.7E-6)	TGAAGATAAAACCTCACAACTTCAC	TAGATAATTCCTGTTTTCT	lead	AS	1745995	1746010
hemH(184.8E-6)	AGTCTATTTTGGTCAACTACTTCAC	TTAATACTCTAGTAAAGTC	lead	AS	1608810	1608825
menX(181.9E-6)	GTCCCCCAAATACGGACAGCATAAT	CAAAACCATCAATAGTAAT	lag	AS	736367	736382
hpt(178.1E-6)	ACATCTAGGATTAATTTTACTTCAC	CAGAGGATTTTGTTCCGCC	lead	AS	25358	25373
yqbK(178.1E-6)	TTACAGATTTATGCCAATACTTCAC	CAACTACTGGTACTTTTAA	lag	S	1619709	1619724
Intergenic(175.3E-6)	AACTGTTACACAAGAAAAACTTTTC	CAAATTTTTTTATGGAAAA	lead	.	841200	841215
ybcH(173.4E-6)	AATGATACTTATGTCCCTACTTCGA	TGGTTTACGAAAATTATAA	lead	S	123666	123681
def(172.4E-6)	AAGGTTACATCATTTGCCACTTCAC	GTAAAGTTGGATAACCTTC	lag	AS	555416	555431
L200156(172.4E-6)	CTTGCGGTTAGGCAACCTACTTCGG	GTACTCCCAACTCCCGTGG	lead	AS	2344471	2344486
L200146(169.6E-6)	GCAGGCGTCACCCCCTATACATCAC	CTTGCGGTTTAGCAGAGAG	lead	AS	1982437	1982452
yyaL(167.7E-6)	CCAACATATTCTTTTCCTTCTTCAC	GCAAGCGACCGGCTTCACG	lead	AS	12154	12169
pepA(165.8E-6)	AAGAAACGAAGCGTTGTTCCTTCTC	CAAGACGACCGTTGTCATC	lag	AS	394211	394226
ydbA(163.0E-6)	ACACTTAAAACAGTGGCAACTTCAC	ATGCAGCCAGCACTTCTAC	lag	S	311295	311310
lcnC(162.0E-6)	TTTTAATGCCTTGCTTTCTTACTTT	ACAAATCCAATTACTAATA	lag	S	83878	83893
infB(161.1E-6)	TGTTGAGTGATACCACCTGCTTCAC	CCTCAGTCACACGAGATTC	lead	AS	777180	777195
sdaA(160.1E-6)	AGGAATAATTTTTAGAATCCTCGAT	TAATTCTTCTATATTTTTA	lead	AS	844130	844145
gyrB(157.3E-6)	CCTGATTTTTTACGTGTCACTTCAC	GCGCACGTTTTGCTGCAAT	lead	AS	929120	929135
yqcA(155.4E-6)	TAAAAGTTCAAATGACCGACTTCAC	AACAGCTGATAGTAATTTA	lag	S	1624880	1624895
recF(155.4E-6)	TCAAGCATGATTTTATTACCATGTT	CAGCTAATTGTCCGTCAAG	lag	AS	2051618	2051633
ydgC(153.5E-6)	CAGGCGTCAGGACAGGTTACTTCAC	TCAGTGGTCTTATTGGTTA	lead	S	362514	362529
pbp2A(153.5E-6)	GCTGCTTCATCAACAGTTACTTCAC	TTGCTGGAACACCAAAGTA	lead	AS	2178563	2178578
dnaE(152.5E-6)	AACGCATCTAAAATCATTACTTCAC	GTTTGCGATCACGTAATTG	lead	AS	496795	496810
nucA(152.5E-6)	TATGTAAAATGCTAAGTTTCATTAT	CATAACCAAACCTATATAT	lead	AS	1100790	1100805
intergenic(151.6E-6)	TCAGGAACAAAGTCATTTACTTCAC	GATTGAGCCTATAAGAATA	lead	.	1213718	1213733
clpC(147.8E-6)	GTCAATTGACCACCCTCATCATAAC	CTACATACCCTGGAGGAGC	lead	AS	631593	631608
clpB(146.9E-6)	AGGTGAACGTGAAAAATTACTTCAC	TTGCCAGAAACACTGCATC	lag	S	1567554	1567569
vacB1(145.9E-6)	GAAAATTATACGCACTTTACTTCAC	CAATTCGTCGTTATCCCGA	lag	S	968723	968738
proB(145.0E-6)	TCAACGAACTCGTTCCAATCTTAAC	TACAATTCGTTTAACACTC	lead	AS	1652644	1652659
pacB(144.0E-6)	AGCCTGGGAAATACAAAGTCGCACC	CATTAACCCTTTTTCGTTG	lead	AS	1903831	1903846
prsA(141.2E-6)	AACTTGCGCGCACGTCCTACTCCAC	CATGGTCAGGCGAAACGAC	lead	AS	826166	826181
ymdE(141.2E-6)	CTTCAAAGACGTTGGTCAACTTCAC	GGAGGAATTGCCACATACG	lead	S	1238775	1238790
ps201(137.4E-6)	TATGGATTTTTTATAAAAGCTCCAC	CATTCTTCAGCTATATCTG	lag	AS	503656	503671
yujG(137.4E-6)	AACAGTAGAGCAACACTTTCTTAAT	CATTTCTCTAACTTCAGTA	lead	AS	2099884	2099899
pi301(136.4E-6)	TTCTGCAGATACAGATCCACTTCAC	GAAAGATTATATAGAGTTT	lag	S	1414528	1414543
ddl(134.5E-6)	AACTCAACATCTTTCACAACTTCAC	CTGGAAGCGTTGCAGAAAC	lead	AS	341407	341422
purC(133.6E-6)	GATGAAATTTCACCTGATACTTCAC	GTCTCTGGGATGCGAATGG	lag	S	1577698	1577713
ycjB(132.6E-6)	TTTTTCAAATTTAATGTTACTTCAC	CAAGTAATATTCCGTCAGC	lead	AS	291861	291876
gltB(132.6E-6)	AAGAAGTCGTTGACAAGTACTTCAC	AGGTACGAGTACAAGAATT	lag	S	1318756	1318771
ythB(132.6E-6)	ACATAATTTATCCTTTCTTCATAAT	CGTTGAATTAACCTTGTGA	lead	AS	1974292	1974307
yhfB(130.8E-6)	TAGCCACCGCTGCTCCAAGCGCATC	CATATCTGTAAAGCGATGT	lead	AS	752126	752141

ptnC(130.8E-6)	CATTGGTCAAGGATACCTTCAAGAC	CAGCAAGGAAGGCAACAAC	lag	AS	1763328	1763343
potB(127.9E-6)	TCATGACAAGCAACATGGCTACAAC	TAAAACAACAGCAATGGTT	lead	AS	1177686	1177701
ylfD(126.0E-6)	TGCTGTTTATAGGCAGTATCTTCTC	CATCCTTGCTTGCTGAATT	lead	AS	1154773	1154788
yrdB(124.1E-6)	GTGACGAGATAAGGGTTTTCTTATT	CATATGGGCTAACTCCTTC	lag	AS	1748651	1748666

Table S4. Multiple alignment of the chromosomal insertion sites of Ll.LtrB intron in the presence of the relaxase (D-pLtrB strain) arranged by the frequency of the event. Names of the genes and relative frequencies are given in which insertions of Ll.LtrB were detected; the orientation of the insertions relative to the direction of the replication (leading strand, lead; lagging strand, lag) and transcription (sense, S; antisense, AS) as well as genomic coordinates are indicated on the right. The sequence of the homing site and location of the insertion are shown on the top. T+5 is highlighted in red.

	IBS2 IBS1	insertion				
homing_site	CGTCGATCGTGAACACATCCATAAC	CATATCATTTTTAATTCTA				
citE(60.5E-3)	CAGGATAAGACTTGGTTTTCATGTC	CACGCGGTAGTCCATACCA	lead	AS	1208563	1208578
arcA(51.2E-3)	ACCCATTACCAAATGCCTACTTCAC	ACGTGACCCACAAGCTTCA	lag	S	2115042	2115057
L200140(35.8E-3)	ACGGATTTCGAGTCCGCTGCATTAC	CACTCTGCCATGGCTCCAC	lag	AS	126580	126595
purA(26.5E-3)	GTTCCATATTCATGTCCTACTTCAC	GAATTTGATGTCCAACTTC	lead	AS	2029172	2029187
als(25.7E-3)	ATTGCTTCAGCATATTTTACGTAAT	CAACATAGCCAAAATCAAC	lead	AS	1201917	1201932
menB(22.9E-3)	ATGGAACAACAGTATTAACCATTCC	CATGTCCATTGCTTCTTGC	lag	AS	734626	734641
ycgD(17.2E-3)	AGCTGAATCATAAACTCTACCATAA	TCACCAGCAACCGTTGGAA	lag	AS	263794	263809
murC(16.9E-3)	GTAATTTCATGATGGTCTACTTCAC	GTGCTGAACCGTAAATTTG	lead	AS	2118446	2118461
rpsI(15.3E-3)	TGTGGGATGTAGCTTTCTACTTCAC	GTCCGTTAACTGTGATTTT	lead	AS	2346610	2346625
yccB(14.8E-3)	TGTGGATAATACATTTTTTTCGTTA	CTATCAGTTGTAAATAAAT	lag	AS	220464	220479
ldhX(13.5E-3)	ATAAAGATATCCAAACCTACTTCAC	GAATTTCTTGATCAATTTC	lead	AS	1143058	1143073
malG(12.4E-3)	CATTGACGTAGAATTTTTGCATAAC	TATGAATAAGATTGTAATA	lag	AS	1742504	1742519
malG(12.2E-3)	AACACGTAGAAACCTAATCCAGAAC	CACCTACATAGACCAGTGC	lag	AS	1742154	1742169
yudI(12.2E-3)	GCTTCAACCAGAGTATCTACTTCAC	CCCCAAAAATTTGCATTGA	lag	AS	2036117	2036132
pi242(11.5E-3)	CGCCCTTCGGTTGCGACTACTTCAC	GTTGAAAGGCACGATATTG	lead	AS	1061365	1061380
yafC(11.3E-3)	ACAAGTTCAGCCTCATTACCATAAC	CATGTTCTGACTGACTAAA	lead	AS	55665	55680
L200119(10.8E-3)	CAAGCCGGTGTGTTAACCACTTCAC	CACATCCGCCATAATGAAA	lead	AS	2340666	2340681
citC(10.1E-3)	ATGGATTAGCATTCATGACCACTGC	CGCGATTTTTTTCGCACCA	lead	AS	1207184	1207199
yvdD(9.9E-3)	ATTACACCAAGTCCATAACCCCAAC	CAATAATCATCCAAGATTG	lead	AS	2134373	2134388
llrB(9.4E-3)	TCTGATACAATAGTTACTTCGTAAT	CATTTTGCCGAAAATTATA	lag	AS	1458230	1458245
clpP(9.4E-3)	ATGGCAAGACCAGCAGATACAGAAC	CACCAGGTGTATTTACGTA	lag	AS	672765	672780
citD(9.1E-3)	CTACCGCAATCGCTCTGGCCTTAAT	CACCAAATCCAAAGCCCCT	lead	AS	1207998	1208013
yljG(8.7E-3)	AAGTCTTCAGATTCTCTTTCGTAAT	CATGAGTCTTTTGAGCCAT	lead	AS	1197156	1197171
yahG(8.1E-3)	GCAGCGTCAGCTTCGGCTTCATAAC	CACCCATTTCGCCAAATTC	lag	AS	74462	74477
citC(7.8E-3)	GTCGCTGAAATGATTTCTTCATTAT	CATCAGCTTTTTTTCGCTC	lead	AS	1207623	1207638
ysaA(7.5E-3)	GGACCTGTCATTTTTGCTACTTCAC	TCACAGGAGGAAGCGGGAA	lead	AS	1806395	1806410
yxbC(7.3E-3)	TCACGATGAAGTGCTTTTTCTTGAT	CTTCACCCGCTTCAATTTC	lag	AS	2316615	2316630
uvrB(6.9E-3)	AAAATTGCCAAATGGTCTACTTCAC	CTAAAACTTGTCCGGTCAA	lead	AS	556835	556850
yljE(6.4E-3)	TCGACGGACAACAATATTTCGCAAC	CAACCTTTTCTTGTTTTCT	lead	AS	1194711	1194726
yuhE(6.1E-3)	AGTTGGTCAATTGCTTTTTCTTGAT	CAGTGACTTCATCAAAAGC	lag	AS	2073393	2073408
gntR(6.0E-3)	TGAATTGAAAGCCTCGCTGCACTAT	CAATATTCGCAATTAAATA	lag	AS	2271599	2271614
lcnC(5.9E-3)	TTAGGCGCTAGTGAAGCTACTTCAC	AAGAAGAGATTATTAGAGC	lag	S	84331	84346
citF(5.7E-3)	GATTACCGACCGCATCAATCTTAAC	CACGTAATCGACTTGCGTT	lead	AS	1209659	1209674
citE(5.5E-3)	CCTCAAAAATTCCTGCATCCTTAAT	CATGGCTGGGTTATTCCCA	lead	AS	1208134	1208149
queA(5.4E-3)	GCATCAACAACTTTCCATTCATAGC	CAGGTTTAATGAAAATGTC	lag	AS	1617614	1617629
yyaL(5.1E-3)	ACGTGAATGATGGCATCTACTTCAC	GAATATTGGCAAGAAATTT	lead	AS	11386	11401
citE(5.0E-3)	GCGCGATTTCCAGCGCATTCATGAC	GCCAAGAGCAGACTCAAGT	lead	AS	1208482	1208497
L200150(5.0E-3)	TGTGTACAAGGCCCGGGAACGTATT	CACCGCGGCGTGCTGATCC	lead	AS	2221784	2221799
intergenic(4.5E-3)	TGTTGTAGTTTATTACCTACTTCAC	TTCTATTCTAATAGATATT	lead	.	1299735	1299750
menB(4.0E-3)	GCGGCAAGTAAACCAGCACCATAAC	CAGCATCAAAAGAACCAAC	lag	AS	734727	734742
ptnC(3.9E-3)	AGGAAGAAGAATGGCCATACTTCAC	GAGTAGCCATAAGGTTGAT	lag	AS	1763895	1763910
typA(3.9E-3)	TTGATTGTTCCACGGAAAACACGTC	CAATACCAATACGTCCAAC	lead	AS	2093826	2093841
ywaB(3.9E-3)	AAAGTCAAAATCACACCTTCACAAT	CAAGTTCAATTGTTCCTTT	lead	AS	2201396	2201411
codY(3.8E-3)	GAAGCGATAACATGCCCTTCTTCAC	CATCAAGCTCTTCAATGAT	lead	AS	164615	164630
plsX(3.8E-3)	GTGACACTTGCTTAATTCTCTTAAC	CAAAAGTAAGCCGGCGGTT	lag	AS	71836	71851
yljJ(3.5E-3)	TCATACAAAGCAGCCGCTTCAGAAT	CATTTTCCAAAGCTTTATG	lead	AS	1200077	1200092
ysiC(3.4E-3)	CGATTGATTAGAAGAGTTGCATAAC	CATGACCTCTTGCTTCTTT	lag	AS	1884434	1884449
gatB(3.3E-3)	CTATTCAAATATTGAGCCACTTCAC	CTTGCAACCAGTTTGACAC	lead	AS	168767	168782
yijC(3.3E-3)	GAACCTCTGCCCATTCCTCCTTCAC	GTACACCAAATCCACCAGC	lead	AS	893748	893763
acmA(3.3E-3)	ATTCGATTCAGGGAAGCACCATAAT	TAGGGTCAGTTGCATATTC	lag	AS	269483	269498
clpC(3.2E-3)	GTCAATTGACCACCCTCATCATAAC	CTACATACCCTGGAGGAGC	lead	AS	631593	631608
apt(3.2E-3)	TGCATTAGAACTTTATAATCGTAAT	CTCCGATTTTTTCGCGACC	lead	AS	623294	623309
proC(3.1E-3)	TCTTGAACTGTAGCATAAGCAGAAT	CATCAACAAATTCATTTTT	lag	AS	1953085	1953100
aroC(3.1E-3)	AGCTGTGGCAAAAAAACTACTTCAC	GAATTAGAAATTGAAATAG	lag	S	1810915	1810930
citD(3.1E-3)	TAGCTTCAATCGCACGTTCCACTAC	CGCAATCGCTCTGGCCTTA	lead	AS	1208019	1208034
ysgC(3.1E-3)	AATGTATTATAGCGAGCCACTTCAC	GCGCAATTGCCGCCGGAAT	lead	AS	1870118	1870133
yjgC(3.1E-3)	TTGCCAAAGAGGAAATATTCATAAC	CGTCATGTTCAGCATCACC	lead	AS	962933	962948
citF(3.0E-3)	GCGCCACGAATGACACCGTCAGAAC	CAGACATAACGTTAACGTT	lead	AS	1210134	1210149
ycgE(3.0E-3)	CTGAACTATGTTCACCCCACATAAT	CATGTGGAAACCAGTTTGA	lag	AS	265242	265257
yvdF(3.0E-3)	TTATTTGCCGCAATTTGTGCACGAC	CACCGTCTAAAGCAGCTAA	lag	AS	2138104	2138119
als(2.9E-3)	CCAAAATCAACGGCTGCTGAACGAC	CATATTTCATTTCTTCTTG	lead	AS	1201884	1201899
ycjC(2.9E-3)	AATCGATAGTGAACACCTTCTCGGT	CATGTTCAAGTTCAATGTC	lead	AS	292573	292588
glnQ(2.8E-3)	TTTTGCATAACGGCTAGAACGTCAC	CAACCATTTCAGGGTCAAG	lag	AS	1819568	1819583
feoB(2.7E-3)	CGGAGAGAATAATCATGGACATTCC	CAAAAAGTAAGCACTAGGA	lag	AS	190731	190746

malG(2.7E-3)	AATACATTGCAATCATTCCCATGAT	TCCTGGGAACATGCCAATA	lag	AS	1742069	1742084
ywaG(2.7E-3)	TTTGACGAATGGTATCTTCCATTTT	CATTGGAAAACGTTCCGCC	lag	AS	2207986	2208001
noxC(2.7E-3)	TTGACAAAAGCTGCCATTTCGCGAT	CAATGGTTGGTAAAACATG	lead	AS	794587	794602
noxA(2.6E-3)	TACGCCAATCAATCAATTCTCCGGC	CATTTCAATTCCAGTGAAT	lead	AS	840414	840429
L200091(2.6E-3)	GCAGAGCCATGTGTTACCACTTCAC	TACGCCGCCACAACATTAA	lead	AS	424310	424325
ycgE(2.5E-3)	ATTACTTTAGCAATTTCTTCTGAAC	TATGTTCACCCCACATAAC	lag	AS	265223	265238
glpD(2.5E-3)	ACCTTGATAGCATCTACTCCTTCAC	GCATAAACAAGATGTGGTT	lead	AS	1270237	1270252
hrcA(2.4E-3)	TCTGCAAGATTGTCAGTTGCATAAT	CAAAGATATTTTTACGCCC	lead	AS	977477	977492
intergenic(2.4E-3)	ATAATCTCTTGGATTACTTCACAAT	CATTTTATCAGATAATAGA	lead	.	1200372	1200387
fabG(2.4E-3)	TATTTTCAGTGAAATCAGCCGCATT	CATCGGTGTATTGATGGCA	lead	AS	802054	802069
ysjA(2.4E-3)	AACTCAGCAATTTATGCCACTTCAC	GCTCACTCTATTCACGCAT	lag	S	1890475	1890490
yljG(2.4E-3)	GCCAAAAGATTTCCATTAGCACAAC	CAACATCTAAGATACTTGA	lead	AS	1197300	1197315
citD(2.4E-3)	CTGGCGCAATCATGATTTGCACATC	TGAAGACTCCAAAGTCCCC	lead	AS	1207824	1207839
mgtA(2.4E-3)	TGGTGACAAGCATCATATACATCAC	TACTGTTAAAATTAGCCAT	lead	AS	1285837	1285852
malF(2.4E-3)	AAAATATAAGTAAACCAATCGCTAC	TATTGCAAAAACACCAAAT	lag	AS	1740665	1740680
yvdE(2.4E-3)	AGCCCAAGGCACTTTCTTGTTCAAC	ACTAATATGATGAGTTGGA	lag	AS	2137423	2137438
nrdF(2.3E-3)	TCATCAGACATGGCTTCCACTTCAC	CAAGAAGGTATGAATTACC	lag	AS	1001348	1001363
pspA(2.3E-3)	CCATTATATAATGCCACTACTTCAC	AGGCTTGATAAGCTCCGAT	lag	AS	2304622	2304637
ywaG(2.3E-3)	ATTAGTCCTGTTCTTTGTCCTTATC	TATTTTAACATAACTTTGT	lag	AS	2208118	2208133
yhfB(2.3E-3)	ATTTATCAAAGTCAGTAGCCATAAT	CATTTTAATTAAATCGTTA	lead	AS	752669	752684
rpoE(2.3E-3)	ATGTCAACTTCTTCATCTACATTAT	CAAGTTCAACTTCTGCAAG	lead	AS	624031	624046
ycgD(2.3E-3)	AATCACCCAAATAACCTTGCTCAAT	CACTTTTTTGACGGTTAAA	lag	AS	264275	264290
potA(2.3E-3)	CTCAATTGGTTCATTTTTACGCATC	CCACCATCGACAGCTTCAA	lead	AS	1176465	1176480
yudI(2.3E-3)	AGTGGATGAGGGACCCCTTCAAAAT	CCATCATTTCCAAAGTCTT	lag	AS	2036072	2036087
clsA(2.3E-3)	TTTGTAATTGAAGCATATTCATCAC	CAACATTAAATCCACCTGT	lead	AS	987987	988002
intergenic(2.3E-3)	TATATAAGTCCCTGGGCTACTTCAC	TTTTGGTTGAAAGTAGCTC	lead	.	133745	133760
yveC(2.3E-3)	TTTTGTTTTTTTAAAATTCCATGAC	CATATTGTACCAAGTTTCT	lag	AS	2144055	2144070
ymcB(2.3E-3)	ATCAGTTCGGAGTGAACAGGTGTAT	CATTTTCGGCAATCCGAAG	lead	AS	1221103	1221118
hslO(2.2E-3)	TTGACCACAACTAAAAAGCCATTAC	CAACAAAAGGAGCAACTAA	lag	AS	2034628	2034643
yahG(2.2E-3)	TAATCATTTCTCCTTCAATCGCAAC	TGAAAGATTTTCTACACGA	lag	AS	73866	73881
ycdJ(2.2E-3)	TTAGAGGCAAGTTCACCAACTTCAC	AAGCAAGCTTAGCTCCAAG	lag	S	237900	237915
plsX(2.2E-3)	CACCAGTTGAGCCGGCAGACAAGAC	TGCATCAGCCTCTCCTTTT	lag	AS	71887	71902
L200140(2.1E-3)	CGCTGCATTACCACTCTGCCATGGC	TCCACTCCTTTATCTAAAT	lag	AS	126594	126609
ycgE(2.1E-3)	GAGTGATTACGGTAATTTCCGCATT	CTTTTTCAGCAACACCAGG	lag	AS	265127	265142
ycjH(2.0E-3)	GTAAAATAATGCCAGCTTCCTCAGC	CAAATTAACTAAATATTGC	lag	AS	295716	295731
ycgE(2.0E-3)	TCGCCTTTAGGCCCAGTTTCGCTAC	CGATTAAGCGGACATAAGG	lag	AS	265430	265445
ackA1(2.0E-3)	TTTGGAAAATTTCACCTCCAGCAAC	CACACGATGTCCTACACCT	lead	AS	2090840	2090855
yrbA(2.0E-3)	CATTTCCTGCGCATTTAATCGAAAC	TTTGTAATCTTGATAGTCA	lag	AS	1710810	1710825
rliB(2.0E-3)	AGCGCTTGAGCGGCTTGCTCACCAC	CATTAAAGTTATCTGAGGA	lag	AS	1535724	1535739
aspC(2.0E-3)	CCTTGAGCTGACAGTATTGCATAAT	CGCTTTACGAGCCGAGAAA	lead	AS	162890	162905
ytcD(2.0E-3)	TCGATATTATTTTCGTTTGCATAAT	TAATAATTTCTCTTTTAGG	lead	AS	1927243	1927258
feoB(1.9E-3)	TTTTTCGGTCACGTTCTTGCTCAAT	CGTTCTCGTCGCCATAATT	lag	AS	190872	190887
ysjA(1.9E-3)	ATGGATTAGTGACAATTTGCATGAT	GAAGATAATCGTTAAAGCG	lead	AS	1890176	1890191
pfl(1.9E-3)	TTCAAATTCTTGATGGATACTTCAC	ACCAGGTGCTTTGATTAAT	lag	S	659540	659555
yhgA(1.9E-3)	GGTCTCGAATTAAGCCAACCATCAC	CCCGTTGTAGCCTAAAGAT	lead	AS	760331	760346
rmaA(1.9E-3)	CTCTGCTAACGTATTCTTTTTCAAT	CAGGCCTTTAATTGCTCGA	lag	AS	749971	749986
queA(1.8E-3)	CAGCAAAAGCAGCGACTAACATGAC	CAAAGTTGATTTTGGTAAA	lag	AS	1617676	1617691
eutD(1.8E-3)	TCACCAAAAATATATTTTTCATTGT	CATCACGACCACGAACCAT	lead	AS	1708550	1708565
ppiA(1.8E-3)	TTGTATTATTTTTTTTATTCGTATT	CATAGGAATATTTTAACAT	lag	AS	369301	369316
pheS(1.8E-3)	GTTTTACTTCGTACGCATACTTCAC	CTATGCAAGCTCGGACAAT	lag	S	2009589	2009604
yseH(1.8E-3)	TCCATGATAACTCAATTTTCCTAAC	CAAAAAGTACATTGAGCTC	lead	AS	1848750	1848765
yxbD(1.8E-3)	AAAGCGTAGTAGCCAATTCCTTCAC	CACGACGTGAAGCAGGGAC	lag	S	2319277	2319292
intergenic(1.7E-3)	GCAACCTTGAAGCAGTGCTTCAAGC	CAACTGAGCTACCGGGTCA	lead	.	56230	56245
yahG(1.7E-3)	CTTCGGCTTCATAACCACCCATTTC	GCCAAATTCTGCCTCAAGT	lag	AS	74472	74487
ywcC(1.7E-3)	GTTGTCAAAGAATCATGTCCACGAT	TAGAAGTATAGAGGAATTT	lag	AS	2229225	2229240
sipL(1.7E-3)	GTGGCCACACTCGTAATTTCGCTTC	ACCAACAATTGCTGAACGT	lag	AS	2350891	2350906
menB(1.7E-3)	CAACAGTATTAACCATTCCCATGTC	CATTGCTTCTTGGGCAGTA	lag	AS	734632	734647
pi234(1.7E-3)	TTCATCCATAATTTGCCTCCTTCAC	GATTTGAACATAGCGATCA	lead	AS	1058576	1058591
yniH(1.7E-3)	GATTTTGTCCACCCATATTCATTCC	CATGAAGGCATTTGTCGCA	lag	AS	1387721	1387736
deoB(1.7E-3)	AAGTCTTCAATTGCTTTTCCGTAAC	CTTCAACATCGCGACGATG	lead	AS	956122	956137
ybeC(1.7E-3)	TCATGATTATGGTCATGTTCGTGAT	CATGTGTATGAGTCATTTT	lead	AS	142347	142362
intergenic(1.7E-3)	CATGGTTACTTCCCATACACATCAT	AAAGTCCGAACGCTTGTGT	lead	.	1712105	1712120
malE(1.7E-3)	ACCAAGAGCAACTTTTTTCCATGAT	TTCATTTTGTGTTTCCTCC	lag	AS	1739039	1739054
ypjC(1.6E-3)	ATTCAACGACTGTCACAATCACAAT	CGTATTAAGATAAAGGCTG	lead	AS	1592521	1592536
ybfA(1.6E-3)	GTTGAGGTAAGGAGTAGTCCAGAAT	CCTGCTAAACCACCTGAGA	lead	AS	157328	157343
ysfA(1.6E-3)	ATCAAGAGAATTGGATTTGCATCTC	CTCGTCCTTTATCAGAAAG	lead	AS	1850293	1850308
pknB(1.6E-3)	TTTGGATAGACTGAGTAAACATAAC	CATCAGCTTGTTGATTCGT	lead	AS	1955328	1955343
yveF(1.6E-3)	CGCAGTTTTTGAGTAATTTCATAAC	CATAAGTTTCCCCTTGACC	lead	AS	2145104	2145119
ylfD(1.6E-3)	CCAACTCAGAGCGAAAAGCCTCAAT	CCCTTGGTTCATATTTTTC	lead	AS	1154976	1154991
yeiF(1.6E-3)	GATAAACCAAGAACAAAGACTTGAC	CAGCTTTAATTTCTGTTTG	lead	AS	486389	486404
lysQ(1.6E-3)	TAGCTAAAAAGAGGACAATCGTAAT	CACTTTAATCGTTGATAAC	lead	AS	370073	370088
recO(1.6E-3)	ATATTTTTATAAACGACCACTTCAC	TGACATCCTCGATAAAAGA	lead	AS	58059	58074
yccB(1.5E-3)	CCATACCAAGCTATACCTTCATAAC	TCCATCCTAATGTGGATAA	lag	AS	220428	220443
htrA(1.5E-3)	GCAAGGTCTGTGTATTCATCATAAC	CAACCACAGAAGCTTTGAC	lag	AS	2204776	2204791
pepXP(1.5E-3)	ATTTCTGCTAAACGTTTTTCGTATT	CATCATTTCCTTTTAAATA	lag	AS	2135926	2135941
ypaE(1.5E-3)	TGGAGCGCTTTTGCCTATGCTAGCC	ATTTCTTTAATTCCTAAAA	lag	S	1505183	1505198
yngF(1.5E-3)	TTTTAGTAAAGATAATCGCCATAAT	CACAAGGGCAATCAATGCA	lag	AS	1367670	1367685
uvrA(1.5E-3)	CCATCAACACGAACACGAACATAAC	CATCTTTTTGGATACGCTC	lead	AS	1887923	1887938
hslO(1.5E-3)	TAATTTCAGCACCGTGATCCTCGGC	TATCATTTCCTCAATTGGT	lag	AS	2035074	2035089
yjhA(1.5E-3)	GGATTTTTTCTTATCATAGGCATAAT	CAAGTGAACGAACAGTTGG	lead	AS	971040	971055
intergenic(1.5E-3)	GTTGTATCTACGCCAATTGCATAAA	CACAAACTATCTGCACCAA	lead	.	1891667	1891682
intergenic(1.5E-3)	GCACGTCATAGAGAGTTTTCATTAC	TACATTTTACCGTATTTGA	lag	.	2100424	2100439
intergenic(1.4E-3)	AGCGCTAGCGGAATGTAAACGTATT	CCAAGAAATGGATAAGCCA	lag	.	2012763	2012778
preA(1.4E-3)	GCAATCGCTTCATCATTTACTTCAC	GAGCCAAGAGTAAAGGTAA	lead	AS	187014	187029
ycgD(1.4E-3)	GGTTGACAATTCGTTTAATCACAAT	ATTCTCACGTTCTTTCACG	lag	AS	264608	264623
glnP(1.4E-3)	CAAAGATAATTCCGACGATCATTGC	TAAGATAAATGAGAGAATG	lag	AS	1818448	1818463
menD(1.4E-3)	AATTGCTGACCAAAATCAGCATAGC	CACTGACCAAATGATAATC	lag	AS	736524	736539
udk(1.4E-3)	TGTGAACGCGTATGTTTTGCATAAT	CATAAATCGGAATATCAAC	lead	AS	1709603	1709618
dexC(1.4E-3)	GTTCCAAAGAAATCTTCTTCGTAAT	CAAACCATCTTTAATCGCA	lead	AS	1737467	1737482
rpsH(1.4E-3)	TACCAGATGTAGGCAATTACTTCAC	CACCAACATTTGTTTCACG	lead	AS	2162238	2162253

yhcA(1.4E-3)	GTGAGCTCAATCGCTCTTTCATGAC	GCTCTTTATCAGATAAGCT	lag	AS	721505	721520
ptnC(1.3E-3)	GAAGATGCAGTACCATTTCCAGAAC	CACCAGAAGCTTGGAGGTT	lag	AS	1764021	1764036
glnP(1.3E-3)	GGGAGATTGATGGGAAGTTCATTGC	TAAATCTTGCCCTTGTTTC	lag	AS	1817470	1817485
noxB(1.3E-3)	AACTTTATGCGTGGTCATACTTCAC	GTCAAGGAAATGTACTTTG	lag	S	842595	842610
trpD(1.3E-3)	TCCACCCAGCGATGAAATACTTCAC	GCCAGTCCGCAGACAACTC	lag	S	1498901	1498916
noxB(1.3E-3)	GTCGCATGTACTCCTGCAACACCAC	CACCGATGACTACGATTTG	lead	AS	841329	841344
intergenic(1.3E-3)	CCCTCTGGGTCAGCTCGTACTTCAC	GCCGTCACTGCACATATTT	lag	.	2016727	2016742
yqiA(1.3E-3)	ACCGCGCTCAAGAGCGCACCCATTG	CCACTACTAAAAGAATGAC	lead	AS	1681716	1681731
murA1(1.3E-3)	GTTGGACGAGGGCCTAAATCACAGC	CACCAGGCAAGCCTACTGT	lead	AS	313400	313415
fhuR(1.3E-3)	CTTGCTCCAAGTCTTTAACCGCAAT	CGACATTGAGGGTTGACTA	lead	AS	330533	330548
asnB(1.3E-3)	GTTCGTAAGAAATCTGCCACTTCAC	GAGCATATTTCAAATCAAT	lead	AS	356847	356862
rpoC(1.3E-3)	GAACGGTAAGTTTTTTGTACTTCAC	CGAGCAAGTAAGTTTCAAC	lead	AS	1857655	1857670
menD(1.3E-3)	CAAGAAGTTGAGCATCCAGCGCTTC	CAAATGTTTTTGTAGTTCT	lag	AS	736927	736942
pstC(1.3E-3)	TCACTTCGAGCAATTCCAACTTCAC	AACGTGAGGGTGGTTTGGC	lag	S	1773284	1773299
ydiC(1.2E-3)	AAGAGAATAAAGCCAAGCAATGCAC	CAATAATAATTAATGGATT	lead	AS	385240	385255
tgt(1.2E-3)	GATTGACGACGTAAATCTTCAAAAC	CAGCTCCTTGAACAATTCC	lag	AS	155934	155949
L200140(1.2E-3)	GGTTCGACGGATTTCGAGTCCGCTG	CATTACCACTCTGCCATGG	lag	AS	126575	126590
hrcA(1.2E-3)	ATAAATTGATTTGTTCTTACTTCAC	CAGTCCCTAATGTTATAAC	lead	AS	977222	977237
intergenic(1.2E-3)	CATTTTACTGAAGTGCTTTCATTTC	TATTGTAATTATAACTGAT	lag	.	1712489	1712504
ybaI(1.2E-3)	CTGCGAGCACCAACACTATCATAAC	CTTCTTGAAGTTTCTGATA	lag	AS	110048	110063
als(1.2E-3)	CTCCTTGCTCATGACGAGTCACGAC	CATTTGAGGGCCTTCTTCA	lead	AS	1200572	1200587
kinB(1.2E-3)	TGGTCAAAAGCAGTGTATCCTGAAC	TAAATTCATCAGGCGTGAA	lag	AS	1459925	1459940
intergenic(1.2E-3)	ACTCGCTATAGCTTATTTACGTAAC	CATGACTTTTAGGTAAACG	lead	.	1838537	1838552
gidB(1.2E-3)	CCTCGAAAATGTGACTTTACTTCAC	GGACGAGCTGAAGATTTTG	lead	S	1381124	1381139
dexA(1.2E-3)	GCTTGATAATCAGAAATATCATAAC	CATTATCATCCATTGGCGA	lead	AS	1736507	1736522
dtpT(1.2E-3)	ACTAACCAAAGAGCACTTGCACGAC	CTGAAGTTCCGTTCAAGAG	lead	AS	705591	705606
glnP(1.1E-3)	CCTGACATCATTCCGTCAGCATGTC	CAGATTGAACAGAGTCAAC	lag	AS	1817828	1817843
citF(1.1E-3)	GGTGCTTTACCGTCCATGCCGTTGG	CATTACCCATTTCGTCAGC	lead	AS	1209507	1209522
xerD(1.1E-3)	CATAGAACTCAGGAAATTCCTTAAT	CATTGGATGATAACTTTGA	lead	AS	1310409	1310424
feoB(1.1E-3)	GCCCCTTCAAGTCGATGGTCATAAT	CTAAAGTCTGACTTTCATG	lag	AS	191636	191651
cydA(1.1E-3)	TTTGATAAGCTGAAATACCAGCAAC	CACAAAACCACCAGTTACA	lead	AS	708282	708297
citB(1.1E-3)	CCTGATAATCGTGTTGCTACTTCAC	TTAAATCAACATTAATCGT	lead	AS	669535	669550
uvrA(1.1E-3)	GCAAATAGTCATTAATTTCCGTAAC	CGTCCCGACTGTTGAACGA	lead	AS	1888140	1888155
yudK(1.1E-3)	TCAGTTCCTGAATAGGCTCCTTCAC	CGTGTAAATATGTTGCGCT	lead	AS	2038474	2038489
ysdA(1.1E-3)	GTCCGATGATTCCCACATACATCAT	CATCAATGTGAAAATTAAA	lead	AS	1834288	1834303
pbuX(1.1E-3)	GGAGCAACAGATTGAAAAGCAACAC	CAAGGACAACTGGTAAGCC	lead	AS	1160591	1160606
purA(1.1E-3)	GGTGTTTGTAAAGCCTATACTTCAC	GTGTGGGTGATGGTCCATT	lag	S	2029214	2029229
ansB(1.1E-3)	CTGTTTTGATTCGATTTTTCAAGAC	CAACATATCTGATAAAGTG	lag	AS	743480	743495
plsX(1.1E-3)	TTTCAGTTGTTTCAACAATCTCAAT	ATTTGTTTCATCGTCTAAA	lag	AS	72013	72028
citC(1.1E-3)	GCGTAAATGGATTAGCATTCATGAC	CACTGCCGCGATTTTTTTC	lead	AS	1207190	1207205
yrfA(1.1E-3)	TTTGAGTAAAGTAAGAATCTTCAAT	CGATATTCATTTGATAGGG	lag	AS	1750155	1750170
busAA(1.1E-3)	CAGCAATAGCTTGTTCACTCGTAAC	AACACCACGGAATTGACGC	lead	AS	1474713	1474728
ysjA(1.0E-3)	CTTGATGGATGACCATTTTCCCAAC	ACCATACATGACAACATAA	lead	AS	1891247	1891262
sdaB(1.0E-3)	GTGTATCAACTTCTAAAATCATAAT	CGCTTTTTCACCCTTAGCT	lead	AS	843991	844006
pcrA(1.0E-3)	TACCCGAACCAGCTCCTGCCATAAT	CAATAAAGGCCCTTCTGTA	lead	AS	1133849	1133864
ymdE(1.0E-3)	AAATGCCCTAAGTCAAATTCATAAT	CATTTCGTGCATCAATCAC	lag	AS	1238993	1239008
pacL(1.0E-3)	TTTCATGATCCCCTGTAATCATAAC	CACATTAATATTTGCATCA	lead	AS	677844	677859
yrfE(1.0E-3)	CATAAACAAAGAAACCAATCATGAC	CGCAATGATCACAACAATA	lead	AS	1759959	1759974
busAB(1.0E-3)	TGAACAGCTGCAAGTACACCACGAC	CTAAGCCAGGCGCCCCAAT	lead	AS	1473732	1473747
ydiB(1.0E-3)	TTTGCTAGCCCTCCTTACACTTCAC	CAGGAGCTTTGAAGAAAGC	lag	S	383788	383803
yniH(1.0E-3)	TTCGCCAAATTAACGACTGCTCCAC	ACTCGTTACAAGCCATATT	lag	AS	1387926	1387941
ldhX(1.0E-3)	ACTGGCAGAATCACGCTTTCGTTTC	TAAAAATTGCTCTTGTCAA	lead	AS	1143148	1143163
dacA(1.0E-3)	TTAGAATTTTGAGTTAATTCATAAG	CATAATCTGAAATAGGAAC	lag	AS	2356064	2356079
ywaI(1.0E-3)	TATCTTTACCAGAAAATTCCACAGT	CACTTTATTTCCAGATTTG	lag	AS	2209257	2209272
ywiA(1.0E-3)	TCTTGCGCAAATTCACGAACATCTC	CAATATTACCTGGATTTAC	lead	AS	2280342	2280357
yrjD(989.1E-6)	TTTCTTTACCAATTTTCCACTTCAC	CAATTGGTCTGAAATAACA	lag	AS	1793542	1793557
yweE(984.5E-6)	TAAATCACGGACAATGAGCATTTTC	CAACGGTCGCCCATCAAAA	lag	AS	2247499	2247514
intergenic(976.0E-6)	GACCCCCTTCAAAAATATCCATGAC	TACCCTTTCACAAAAGAAA	lead	.	1210567	1210582
ykiF(975.7E-6)	TCTAAATCAAGCCAATCTTCAGAAC	TCAAAGTTTCTTTGATAAA	lag	AS	1087045	1087060
yuhI(971.4E-6)	ATATCAAGGAAGGCAAGAACATCAT	CATCTTCATAAATTTTGGT	lag	AS	2077617	2077632
thrA(968.9E-6)	ACTTTTCGGCCGAATCCAACTTCAC	GGTTCATCAGATATTTACT	lag	AS	747685	747700
citG(967.4E-6)	GCACATTCAGCTTGACAGACACAAC	CGTACTCTCAGGAAACTCC	lead	AS	1210681	1210696
birA2(953.2E-6)	GTTAAATAAAGACAATTTTCTTGTC	CGTCTAATTTAGCATCTTT	lag	AS	1973041	1973056
aroH(943.5E-6)	ACTGAGCGAGCGCCAACTGCATAGT	AACTTACTAAGTCATCAAC	lead	AS	1280932	1280947
intergenic(941.5E-6)	AAATAATTGCAATTTTTTTCATGAC	TCGATTATACCATAGTCTA	lag	.	2355772	2355787
yudH(937.4E-6)	CTATAAAAAGCACCATAGCCGTTAT	CACCCTCACCCTCAACATC	lag	AS	2035315	2035330
yiaC(935.3E-6)	TCATCGTTAGAATAACATACAGGGC	CGCAACAATTGCGATTGTA	lead	AS	802688	802703
queA(934.9E-6)	TAAGCGTCAAGAACAAAATCACGGC	CAGCAAAAGCAGCGACTAA	lag	AS	1617701	1617716
intergenic(934.7E-6)	CAAAGGCTTGTATTTTTTACACTTC	TATCGTACAATAATTTTAC	lag	.	1562466	1562481
ysjD(925.9E-6)	TTTTTTACCTTGTCAGCCACTTCAC	CTGCTTCACCAGATAATCC	lead	AS	1893720	1893735
yriB(925.7E-6)	AAATGATAAACGCCCCAATCGTAAC	GTTCTTAAATCCTTCAGTT	lead	AS	1786925	1786940
clsB(917.3E-6)	GAAGCCAAATATAGTCTTCCGCATC	CATAATCATTTTAATAAAA	lead	AS	1188112	1188127
yeaC(913.3E-6)	TGGAGATCATCCTCATTTACTTCAC	GCACAATCGTGTGGTGAAT	lead	AS	405707	405722
typA(909.9E-6)	ATTGACAAGTGCAATTCACCACGAC	CAGAAACAACCCATTTATC	lead	AS	2093418	2093433
fbaA(902.0E-6)	TCACCACCAATTGAACCAACTTCAC	ATTCAACTGAGATGCCTTT	lead	AS	1979895	1979910
pbuX(893.7E-6)	AATGAACCAAACATTGCTCCAGAGC	CATGTCTTTGACCAATAAT	lead	AS	1160642	1160657
yecE(885.8E-6)	TTTCATCAAGATTTACATACATATC	CAAATGACCTTTGAGATTA	lead	AS	427516	427531
trmE(884.6E-6)	AAACGAACAATGGCAATTGCTCCTT	CACCAAGCGGTGTCGAAAT	lag	AS	2327788	2327803
pycA(880.8E-6)	TCGTTACCACGCGCATAAACATCTT	CTTCTGTTAAATCATTTTG	lead	AS	666290	666305
fadD(875.8E-6)	CCTGCAGGACATTCATTTCCATCTT	CATCGGCAATACGTGCTTT	lag	AS	654992	655007
llrG(867.9E-6)	ATTTTTTCCTTGGTCACCACTTCAC	CATTTCCACGAAAAAGAAG	lead	AS	1805025	1805040
deoD(858.1E-6)	ATATGAACATCTTCATTTACAGAAC	CAGCAGTCCCAACACGAAT	lead	AS	957081	957096
fabD(857.2E-6)	GCATAATCAACCGCCTCAACTTCAC	CACCAATAACAATTTGTGC	lead	AS	783367	783382
clsB(849.2E-6)	AAATATAGTCTTCCGCATCCATAAT	CATTTTAATAAAACCTGTT	lead	AS	1188106	1188121
L200130(847.7E-6)	TTTGATTAAAAGTCAAATGCTCTAC	CAACTGAGCTAACGAGTCA	lead	AS	1980938	1980953
intergenic(843.7E-6)	CCAACTCTCTTTAGTTGGTATTTTT	TATTTTAAAATAGTTCTCT	lag	.	2275138	2275153
apbE(842.3E-6)	AGTGAACCAATTGTAATATCATAAC	CTAAATCTTTAGCTGAATA	lead	AS	1124691	1124706
yagB(842.2E-6)	CGTTCAATATGGTCAAAGCCATGTC	CATCATTATTTTTATCATG	lag	AS	67086	67101
intergenic(841.1E-6)	AGGAAAATTTACTGACAGCCACTCC	TAAACAGTCTGTCAGTAAA	lead	.	970097	970112
rplM(839.1E-6)	AGTATATTACTCACACTCACTTCAC	CCAGGTGGATTGAAATCAG	lag	S	2346912	2346927

ylgC(837.8E-6)	TCAGGTAAACCTGTATCTTCACGGC	CAAACAAGAAGTAGTGATC	lag	AS	1162425	1162440
ychH(833.8E-6)	TATTGATGATAGCCCCATCATAAT	GACCGCACTATCGCCAATA	lead	AS	279650	279665
yijE(825.2E-6)	TATGAATAAGATACATTCTCTCATC	AAGCGCATGAATTTGCTCT	lead	AS	897434	897449
tgt(824.8E-6)	TCGGGGTTAAGAAAAAGTTCACGTC	CGTCCAAATGGCTCTTGAA	lag	AS	156150	156165
ssbB(811.3E-6)	GAAGTTCCGCCTCCCATACCTTCAC	GAGCAGATCTACTTTCCAA	lead	AS	2274356	2274371
fbaA(804.1E-6)	ACACAATGTGCCAATCGTACTTCAC	GGTGGTTCTGGTATCCCTG	lag	S	1979644	1979659
yjgC(802.2E-6)	TCAGTAAAATTTACTTAAACATTTC	TGCTTTTGGCACGAAATCC	lead	AS	963079	963094
dexC(800.1E-6)	GTCACCGCATACTCATCCCCATAAT	AAATACAAGGCACACCTGG	lead	AS	1737262	1737277
ptsI(799.5E-6)	GTAGATGGATCAAGAATTACTTCAC	CAGTCAATCCTGAAACAGC	lead	AS	121312	121327
pbp1B(794.1E-6)	TTGGATCGGCAGCATTAATTGCATT	CACCAAATTAGCTGTAAAT	lead	AS	393516	393531
intergenic(793.8E-6)	CGTGCGTAAAATTGAGAACCATGAC	CAGGCACTTATTGAGTGCT	lead	.	2012509	2012524
intergenic(787.6E-6)	TAAGAACAACCATGATAATCACAAC	AACCATGGTTACTTTTTGG	lag	.	1931070	1931085
citF(784.9E-6)	TATTGAGCATCCATCAAAGCATAAC	CCAATGATCCAAATGGTGC	lead	AS	1209546	1209561
argR(775.8E-6)	CATTCGCCAACACATCGGCCTCACC	CAATTTTGTCCGAATAACC	lag	AS	2117884	2117899
relA(767.0E-6)	TTTTGTTTGGCTCTCTCAACTTCAC	GACGTTCATTTTCAGTTAA	lead	AS	108865	108880
uppP(766.9E-6)	AAGGTAAATTCTGCGGCAACTTCAC	GACTTACACCAACTAATAA	lead	AS	2275479	2275494
intergenic(762.6E-6)	AAGGCTAAAGCCTTATAATCATTGA	TATTTTCCATAATTTTATT	lag	.	1706685	1706700
gshR(760.9E-6)	TTGGCTGACGGCCTACTCCCGCAAT	CACTAAATCTGTTTTATGT	lead	AS	864313	864328
floL(758.3E-6)	GCTTCGGCCATTGCTGTTACTTCAC	GTTGTTTAGCAGTGGCTTT	lead	AS	746840	746855
asnB(757.2E-6)	GTTTCTAAGTGCCAAATCACTTCAC	GGACTGCTGAAAGTACATC	lead	AS	356922	356937
yrfE(755.7E-6)	TTGATTTTATACCATTTTTCATATC	CAGAAGAGCTGTAATTCCT	lead	AS	1759098	1759113
yxaB(746.0E-6)	TTTGTATTAGTTTCATTGACGTAAT	CATGAGCGTACTGTTCAAT	lag	AS	2301202	2301217
menF(745.8E-6)	CTGCATAAGCATAAAGTTTCTTGTC	CACAATCAAAGCCGAACGA	lag	AS	738243	738258
menD(742.6E-6)	CAAAAGGGAGAACATCAGACTGAAC	CATCGTTGTCGTTGTCAAA	lag	AS	737101	737116
pbuX(735.3E-6)	CAAGAATTGAACCTGAATACATGGC	CAGCAGATGTTGTAATCCT	lead	AS	1160419	1160434
citE(733.2E-6)	TTTCGATTTCGACTTCCAGCTCTTC	CATCATAGCTGCTGTTTCA	lead	AS	1208386	1208401
yljF(730.6E-6)	GTTGGTTTCACTCCTAAAGCATAAC	TAGGTTCAACTGTTCCCAT	lead	AS	1196804	1196819
noxB(724.8E-6)	CCACCAAGGGCAACAATGACATAAT	CATAGCTATAACGACCATG	lead	AS	841599	841614
ynbC(723.1E-6)	TAAGAATAACGGTCTAATCCTAAAC	CAACATAAAAAAGATGACC	lead	AS	1311441	1311456
ysiA(717.3E-6)	TTGGAACCATGATTTCACGCGCAAC	CAACTCGTCTAAGCTGAAC	lead	AS	1882158	1882173
hemH(711.6E-6)	AGTCTATTTTGGTCAACTACTTCAC	TTAATACTCTAGTAAAGTC	lead	AS	1608810	1608825
lysQ(708.4E-6)	CCTAATGCAAAGCCAAAAGCAGGAT	CAACATAACGACCGCCATA	lead	AS	369852	369867
optC(697.2E-6)	CTGATAAAACAGCCTCATACATAAT	CATACTTGGAATATCAAAC	lead	AS	348745	348760
argR(694.2E-6)	TCTGTCCGACTATCACGTTCGTCAT	CTAAAGCATTCGCCAACAC	lag	AS	2117915	2117930
ylcG(690.3E-6)	TGAAAGTGACAGCTCCATTCATTGC	CTCAGGAGAACGTCTCATC	lead	AS	1130988	1131003
dacB(690.1E-6)	GTTCGGTTTAGCATGATTTCATTTC	CTTTATGCTGGTAATAGTT	lead	AS	976126	976141
noxA(687.5E-6)	ACGACTAAATAATCATAGTCATAGC	TATTTTTATCTGTAATCAC	lead	AS	840184	840199
panE(685.1E-6)	CCATCTATAAGGGTTACTTCATTGC	CACCTTTATGAAGCATTAA	lead	AS	1358181	1358196
clpC(681.5E-6)	ACACTTGCTTTATGCCCTACTTCAA	GTAGAAGATGGATTTGGTC	lag	S	630146	630161
ytdB(680.9E-6)	ATATCAGCAGATTCTTTTTCAGAAT	TAATTCTAAGCGTATCAGC	lead	AS	1934966	1934981
ygbD(680.0E-6)	CGAATAAATCTGAAATTTTCATAAC	CATTTTTTCCTTCAAGTTG	lead	AS	616221	616236
L200159(674.0E-6)	CTTGCGGTTAGGCAACCTACTTCGG	GTACTCCCAACTCCCGTGG	lead	AS	2363650	2363665
glnP(665.5E-6)	GAAGAATAGTAAGGACTACCATAAT	CGAAAACTTGTTTACGAGC	lag	AS	1817888	1817903
recD(660.7E-6)	TAGGTCTTTCGATTCGCTCCTTCAC	GCGCCACCGCTTGTGCAAA	lead	AS	1797401	1797416
scrK(651.5E-6)	GCGGAAAATCATTGGTTTACTTCAC	AATTGGAACAGGAATTGGT	lag	S	1518450	1518465
hpt(650.0E-6)	ACATCTAGGATTAATTTTACTTCAC	CAGAGGATTTTGTTCCGCC	lead	AS	25358	25373
alaS(638.3E-6)	TTAGCGTCAGGAACAGTTACTTCAC	TGGCGATAAAGTTGAGAGA	lead	AS	1778652	1778667
htrA(635.8E-6)	GTAGTGCTTTGATAGATTGCACTTC	CTCCAAGTGCGATGGCTCC	lag	AS	2204392	2204407
yjgC(635.1E-6)	GCACGAGCAACCTCAATATCGTAAC	CCGTTAAATTTCCTTGACT	lead	AS	962423	962438
lepA(628.7E-6)	TGCAAAAGGCCGAGGAAACCACAAC	GGAATCCGAAACCAAGCGC	lead	AS	1118394	1118409
rpsM(628.4E-6)	CGTTTGATGTTAAGTGACACTTCAC	GACGAAGGTCTCCTTCGAG	lead	AS	2153631	2153646
yagB(625.5E-6)	TAAAAGACTCGATTTTTTGCCCAAC	CATATTCAAGTGTCCTTAC	lag	AS	66735	66750
ybeH(625.4E-6)	GATGATTATACCATACGGTCATAAT	CATAAAAAAGCTCAAAGCA	lag	AS	143096	143111
intergenic(624.6E-6)	AAACGTCAAAGAGGACTCACACATT	TAGCTTAAATTAGCTAGTG	lag	.	820084	820099
ilvD(621.5E-6)	TCGATGCTGTCAGCAATCACTTCAC	GACTGACCAAAGAATATCT	lead	AS	1246083	1246098
pepQ(603.2E-6)	AAAGACAGTCTTCAATACGCACACC	TACTTTACCAGGAATATAA	lag	AS	1698129	1698144
efp(601.2E-6)	GTTTGTGATGACTTGCTTCCATAAC	GCGGAGAAGTTTTTCCCCA	lead	AS	691670	691685
Intergenic(594.1E-6)	TTTGACTCAGACTCAACCTCACTCC	TGTTTAGTTTAGAAAAGTC	lag	.	2276493	2276508
yqbK(590.7E-6)	TTACAGATTTATGCCAATACTTCAC	CAACTACTGGTACTTTTAA	lag	S	1619709	1619724
gidC(579.4E-6)	CCAGAAGCAGCTGATTCTACATAGC	CCTCTACTCCTGTCATTTG	lead	AS	1257813	1257828
copR(575.7E-6)	TTGCCGTCAAAGTCGATTTTTCAAT	CATCTCTTGCAAAACATTG	lead	AS	845396	845411
citR(570.7E-6)	GCCAAATTGGCGAGAAGTTCATGAT	TATTCATAAAGTAATTATA	lag	AS	1206676	1206691
yoaB(569.0E-6)	TTTTGTGGTCACCCGTAATCATAAT	CGTTTTAATGCCGGCTTTT	lead	AS	1404130	1404145
choQ(557.7E-6)	GGATGAACCTTTTTCAGCACTTGAC	CCAATTTCACGACGTCAGT	lag	S	865510	865525
yseI(557.7E-6)	ACACCACGGTGGACATTAGCATTAT	CATTTTCATAATAATCTTT	lead	AS	1849919	1849934
ygaB(548.0E-6)	ATTCGACTGACAATAAAATCTCGAT	GATTAAAGACTTCATAAGC	lead	AS	602141	602156
malF(539.1E-6)	AGTGGGAAAAAGGCTTTTCCGTAGT	CCCCTTGGAAAACTTGAGC	lag	AS	1740960	1740975
parC(537.4E-6)	TCAGGTTACATCAAACGTACTTCAC	ACGTTCATTTGCTTCCTCA	lag	S	1012443	1012458
yrgH(536.8E-6)	AAATGAGCTAATTCTCGGTCCCAAT	CATAGAAATCTTCCTGCTC	lag	AS	1769355	1769370
ykiF(535.2E-6)	AGCCGCTTTAGCTTTTTTAACAGCC	GTTTTTACTTCGGCAGTAA	lag	AS	1087227	1087242
ps105(530.7E-6)	AAGGGCTTCACTCCCTCATCTTCAC	ACCATGCACGATATAAGGC	lag	AS	37893	37908
yuhE(521.7E-6)	AGTGCGTCAGTATCTATTTCATTGC	CAGGAGTAAGGGCACCAAA	lag	AS	2073300	2073315
yfjF(512.1E-6)	CTTCAAAGTGACTTACATACTTCAC	TAGACATGATTCAGTGGGC	lead	S	599610	599625
leuA(511.8E-6)	ATTCATGTTTTCATTGCCACTTCAC	CTATTCACATGAAATATAA	lag	S	1240133	1240148
nrdD(499.2E-6)	TCATAGTTTGGTGTCCCTACTTCGA	GGTTAAGACCACGTTTGAG	lead	AS	271457	271472
kdgR(492.7E-6)	TTTTGTCCAGGACGATATTCATTCC	TGAGAATTTTGCCTGTAAT	lag	AS	1680250	1680265
pepF(492.5E-6)	TATTCTTTACCAAATATCGCTAAAA	CTTCTTCTGCTTTTTTCAC	lead	AS	1783848	1783863
rplC(486.0E-6)	ACAGTGATTTCTGCTCCTACTTCAG	CGCCTTCGAGTCCTTTGAA	lead	AS	2169681	2169696
comFA(482.4E-6)	TCAACAGTGAGCACCCAAACTTCAC	TAAATCCGCACTGATACAA	lead	S	1097125	1097140
topA(477.7E-6)	AAAGCATTAATTTCATTCTCGCGGT	CAACAATTAGTTTTAAGGC	lead	AS	1255147	1255162
yveC(470.5E-6)	ATTCCTAAGCCTTGATAATCAGGGT	CAACGGTTAAACTGATGAT	lag	AS	2143864	2143879
yeeB(465.5E-6)	TTTGGTCGATGGATTTGTTCAGAAT	TACCATACCAAGCGGCTAG	lag	AS	443166	443181
ybcG(460.1E-6)	CTTGTTTCATCATTGATTGCATATT	CATCATGGAAATATTACCT	lag	AS	123139	123154
yqcA(456.8E-6)	TAAAAGTTCAAATGACCGACTTCAC	AACAGCTGATAGTAATTTA	lag	S	1624880	1624895
gyrB(456.5E-6)	CCTGATTTTTTACGTGTCACTTCAC	GCGCACGTTTTGCTGCAAT	lead	AS	929120	929135
yeeB(453.5E-6)	TTGTGAAATATGTTGATAACCACCA	CCAGGGCAGATAATAATAG	lag	AS	443427	443442
ybcH(450.7E-6)	AATGATACTTATGTCCCTACTTCGA	TGGTTTACGAAAATTATAA	lead	S	123666	123681
ylaD(448.7E-6)	CCAATTTGAACTGCAACTCCTTCAC	GACCAACACTGGCACCAAA	lead	AS	1105705	1105720
mscL(446.4E-6)	CCAACGGCTAAGTCCAATACGTTCC	CACGTAAAATAAAGTTTTT	lag	AS	2170604	2170619
L200065(444.5E-6)	TGAAGATAAAACCTCACAACTTCAC	TAGATAATTCCTGTTTTCT	lead	AS	1745995	1746010

ymdE(437.5E-6)	CTTCAAAGACGTTGGTCAACTTCAC	GGAGGAATTGCCACATACG	lead	S	1238775	1238790
yqgG(432.8E-6)	TTGGAACGGCTATTTCTTTCATAAC	CAATGGCATATCCACATAC	lag	AS	1664100	1664115
yebE(428.7E-6)	TATCTTCACTAATAATTCTGTCAGC	CCTAATATGATGAGTTATC	lead	AS	417456	417471
glnQ(427.9E-6)	CTTCTTCGGCCAATTTTTGCATAAC	GGCTAGAACGTCACCAACC	lag	AS	1819582	1819597
purL(424.0E-6)	ATATCCACAACCCCAGCTCCTTCAC	CAGGCCCCATCAAGACTTG	lead	AS	1576156	1576171
yveH(422.8E-6)	GTACGTTAATATGTTCGCCCACAAC	AAGTTGTGTTTTCACAGCA	lead	AS	2146197	2146212
yrgE(418.4E-6)	GGGATTTTGAAACATCACCCTCAAC	ACGAGCCACTGCCGTCCAA	lag	AS	1765334	1765349
intergenic(408.9E-6)	TCAGGAACAAAGTCATTTACTTCAC	GATTGAGCCTATAAGAATA	lead	.	1213718	1213733
intergenic(405.0E-6)	TAGTTGCCAAAATCGTCCTCATTTC	CGTTTATACCTGTATTATA	lag	.	1766419	1766434
clpB(402.4E-6)	AGGTGAACGTGAAAAATTACTTCAC	TTGCCAGAAACACTGCATC	lag	S	1567554	1567569
infB(401.7E-6)	TGTTGAGTGATACCACCTGCTTCAC	CCTCAGTCACACGAGATTC	lead	AS	777180	777195
pi301(401.6E-6)	TTCTGCAGATACAGATCCACTTCAC	GAAAGATTATATAGAGTTT	lag	S	1414528	1414543
pbp2A(400.0E-6)	GCTGCTTCATCAACAGTTACTTCAC	TTGCTGGAACACCAAAGTA	lead	AS	2178563	2178578
gntR(391.4E-6)	GTATGTGAAATAACAAAAGCACAAT	CGTCGCTCGTTAATTTTGC	lag	AS	2272040	2272055
ymjE(387.4E-6)	CTGTATAGATTGTCAAAAGCATAAT	CACAACAACTTCAAAAATT	lead	AS	1297570	1297585
purC(385.1E-6)	GATGAAATTTCACCTGATACTTCAC	GTCTCTGGGATGCGAATGG	lag	S	1577698	1577713
radA(383.9E-6)	TGCATCAATTCTCCAGTCACTTCAC	GAACTTGACTTACAGAGCC	lead	AS	2149913	2149928
ydbA(381.9E-6)	ACACTTAAAACAGTGGCAACTTCAC	ATGCAGCCAGCACTTCTAC	lag	S	311295	311310
yhdB(381.5E-6)	GATAAATCACCGGCCATAGCATTTC	CAGAAAGCGCGGCGAGTTG	lag	AS	731959	731974
vacB2(379.1E-6)	ATGGTCAGAAGGATATAAATGCAAC	AAACGATGGATGATTAAAT	lead	AS	1227759	1227774
gyrA(368.5E-6)	AAGCGGCGAAATATCTCTTCTTCAC	AACACGTAACGGAATTGTT	lag	S	1123616	1123631
glgP(367.5E-6)	CCATCATCTTCTAACCAAACTTCAC	CACCAAATGTTACTTCAAC	lead	AS	700438	700453
topA(364.2E-6)	TGTAAAATTGGTCAACAACCTCAAT	CCATTGACGTTTTCCTTCT	lead	AS	1256226	1256241
ycjB(360.6E-6)	TTTTTCAAATTTAATGTTACTTCAC	CAAGTAATATTCCGTCAGC	lead	AS	291861	291876
intergenic(355.9E-6)	ATACCGCGGTACCACCCTTATTCAC	AATAGAATTTCTATCATCT	lead	.	1781130	1781145
aspS(352.2E-6)	TTTTTAGGGAAAGCAATTACTTCAC	GGATATTGTCTTTTCCAGC	lead	AS	2040291	2040306
pepA(349.6E-6)	TAGTCACGAACAGATCCCTCAAAAC	CAGAAGTCGCTTGAATTTC	lag	AS	394913	394928
yrjI(345.7E-6)	CCGTGACCAACTAAAAGTACATTGT	CCATTTTAGAATTCAACAA	lead	AS	1799737	1799752
yahI(345.5E-6)	ACAAAAGCTTGGTTTGATTCAGAGC	TAGTAACATCCAGTTTTAG	lag	AS	78146	78161
yyaL(345.4E-6)	CCAACATATTCTTTTCCTTCTTCAC	GCAAGCGACCGGCTTCACG	lead	AS	12154	12169
ywjH(344.8E-6)	TCTGGCCAAACCCCTTCAGCATAAC	CAGAATGGCCGCTTAGGAG	lead	AS	2299894	2299909
apl(341.8E-6)	TAATCCCTAAAATCACAACCACAAC	TGAAGAGTAAGAATCAAGA	lag	AS	719117	719132
ygeA(341.2E-6)	TCACTAACAGAGGTATAATCAGGAC	TAGAGTTCATATAGGTCAA	lead	AS	647001	647016
rliA(340.4E-6)	TTAAAGACAAGACATCATCCATAAC	TACAAGAGCTGTTTCATTT	lead	AS	1728090	1728105
ddl(335.8E-6)	AACTCAACATCTTTCACAACTTCAC	CTGGAAGCGTTGCAGAAAC	lead	AS	341407	341422
gltB(335.2E-6)	AAGAAGTCGTTGACAAGTACTTCAC	AGGTACGAGTACAAGAATT	lag	S	1318756	1318771
prsA(331.9E-6)	AACTTGCGCGCACGTCCTACTCCAC	CATGGTCAGGCGAAACGAC	lead	AS	826166	826181
yfiC(328.7E-6)	AGACAAAATTCATAAAATACGCAAT	CGTCCCATCCTTTGTTATC	lag	AS	582039	582054
ynhC(328.0E-6)	TCTGTCAGTGAAGAACCAACTTCAC	CCAAATCTTGTGCCTGAAC	lead	AS	1375536	1375551
yseF(319.5E-6)	CTTCAACAAAGATTTCAACCACTGC	TGCATGAAGTGAACTTGCA	lead	AS	1846805	1846820
gidA(317.5E-6)	ATGGATTACATGGCATAAAGGCAAC	CATATTAAGATTAATCGTC	lead	AS	1916173	1916188
feoB(317.2E-6)	TAAGTGCTACTTTAACAACCTCATC	TAAACCTTTATTTTTAATG	lag	AS	191685	191700
vacB1(316.8E-6)	GAAAATTATACGCACTTTACTTCAC	CAATTCGTCGTTATCCCGA	lag	S	968723	968738
ahpF(314.9E-6)	TCAAGCGATTCAAAATCCACTTCAC	TTTGAAACTTATGTGAGTT	lag	S	336370	336385
recX(313.1E-6)	ATATCATCATATGAAAAGCCTTTTC	TAGCCAAAGCCTGTGTTAC	lag	AS	2260546	2260561
clsB(312.1E-6)	CCTTATTAAGCCCTATATACAAGAC	CATTTATAATATCGGACTA	lead	AS	1188583	1188598
gntR(311.2E-6)	CCCCGCGACTCTTCAAAATTTCAAC	CAAGTGCATTGTATCTCTG	lag	AS	2272093	2272108
ygiI(310.7E-6)	GTGTAAGCTGGTCTATTTTCTTTTC	CACGATATGGCATTGGTGT	lead	AS	688022	688037
cydB(307.4E-6)	AGCAATACAGCAAATACAACTTCAC	CAGCATAAAGAACAAAATA	lead	AS	709788	709803
tyrS(302.1E-6)	CCGTGAACTAAGCTGACAACTTCAC	GAGCCAAAACTTTTTGGGC	lag	AS	390395	390410
ykhD(301.5E-6)	AATTGATAATGAAGTAATGCACGAC	CGAGATTTCCGACACCAAT	lag	AS	1072414	1072429
busAB(298.6E-6)	TCGTTTGGTTAACACCAGCCATAAT	TGTTCCTTTAGCCAAAGGA	lead	AS	1473814	1473829
glnP(298.6E-6)	CCATTGGCACGGAGATTTGCTAAAC	CATTGTTAAACATTTCAAT	lag	AS	1818260	1818275
intergenic(293.2E-6)	TGACAAACTCCCAAAAGAGTGACCC	CACCAATTGAACCAAAGTT	lead	-.	426554	426569
yrdB(287.3E-6)	GGAATGGAAACTGAATAATCATTTC	TAACATTATTTATTTTGAT	lag	AS	1748835	1748850
yliB(286.4E-6)	GGCATGAGTTATTAAAATACTTCAC	GATTTCGCATAAGATTCAG	lag	S	1183134	1183149
pmsX(284.1E-6)	TCATCATAAGAAATCAATTCATTAT	CAAATAAAATTTCAACTGC	lag	AS	1594083	1594098
ysfB(283.9E-6)	CCTCAGCACGTTTAGCATTCATTGC	TGCTCGCAAAAATTCTGCA	lead	AS	1851913	1851928
aroB(283.2E-6)	ACAACCACTTTTCGTTTTACTTCAC	AAGACCGATAGATAATTTC	lead	AS	1814163	1814178
frdC(283.0E-6)	TTAAGTTCAGCAGCAGAAACTTTAT	CACGAGTATCCATTTCATT	lead	AS	1138856	1138871
cysE(273.6E-6)	GTTTCACCAATAACTAATCCAGAAC	CATGGTCAATAAAGACTCC	lead	AS	1920560	1920575
grpE(270.4E-6)	TTTCCAAAAAGGTTACCAACTTCAC	GAACGTTTACTTCGTCCAG	lag	S	978348	978363
intergenic(269.5E-6)	AATCAAATGTAAAAACTAGACCCAC	TTATTTTAACATAAAAAGT	lead	.	228110	228125
tra983F(269.2E-6)	GCGCACGAGGTTTGATTTTCTTTGC	CGCCTTATACTGCGGATAG	lag	AS	1556004	1556019
dut(267.2E-6)	CGGTCATACATGTAAAGCACTTCAC	CTGCCTGCATATAGGCCTT	lead	AS	181327	181342
carB(267.2E-6)	AGGGCTTGAAGTGCTTTTACACAGT	GAACTGTCGCATAATCAAA	lead	AS	1399755	1399770
lepA(262.4E-6)	GCCTTGAGCGGAGCGTCAACTTCAC	CTTGAGGGGCAGGAACTTT	lead	AS	1117932	1117947
rpsC(261.0E-6)	GATTTTTTAGTAGGGAGAACTTCAC	CACGGTAGATCCAAACTTT	lead	AS	2166108	2166123
ycfD(257.7E-6)	AAGCCGCCTAATCCATCTCCTTCAC	CATTAAAAAGACGAAAGGC	lead	AS	252972	252987
intergenic(256.9E-6)	AATTCCATAATTTTCTAGCACTTTC	CTATATTATGTAATCAATT	lag	.	2071740	2071755
hslO(256.3E-6)	GCGTTCCTGTGCTTCTTTCACAGTT	AGGGTTGAATCCAAAGCAA	lag	AS	2034366	2034381
atpC(254.9E-6)	CGCACGTGATACATCAATATCACGA	TTACGTTCTGCAGAGTCTG	lead	AS	1822759	1822774
yhcH(253.6E-6)	ACATCAAAGCCAAAACGGCCATGAT	CAATGCTATCTCCCAAGAC	lead	AS	726528	726543
pepT(253.1E-6)	AAAAGATGGAAGAAACCTTCACGGC	CTTCGGTTTTTTCAGGACG	lead	AS	1877702	1877717
ylhB(248.3E-6)	GAGGCAAGCGTGAATGATGCAGGAC	CAATAATAATTTTAGCCGG	lead	AS	1173578	1173593
intergenic(246.6E-6)	ACATCCAAATTTTCTTTTTCATAAA	CATCTCCAATTGTATTTTA	lag	.	2208612	2208627
apl(243.5E-6)	GAATTGGTGGGAAGATGTTCTCAAT	CATAATGAGAAAGGCAACA	lag	AS	719588	719603
pspA(243.5E-6)	AGAGAGGAGTGCATAATAGCATTCC	CCAGAAGTTGTATTAAATT	lag	AS	2304554	2304569
hemN(240.9E-6)	TATCCATGATATATTCATACATGTC	CGCATTTTTATCTTCACTT	lead	AS	1153916	1153931
ymcF(239.7E-6)	TTCCTTCCTCATCAACATACTTCAC	CATTACTCCTTTAGAATAA	lead	AS	1223916	1223931
proS(239.7E-6)	TCTTTGTTAATTTCAACAACTTCAC	CTGTCGCACGAATTTTAAC	lead	AS	2196190	2196205
rplI(238.7E-6)	AAAAGATGTACCAGTTAAACTTCAC	CGTGATGTGACAGCCGTGG	lag	S	753463	753478
fhs(238.7E-6)	GGTAATACCTGTGCAAAACCACCAC	CAGCAGCTCCACCCTTAAT	lead	AS	960069	960084
yrfE(237.9E-6)	TGTTGACGTTTTACTCGTCCATAAT	CTCCCTCATCATCATGACG	lead	AS	1759268	1759283
ispA(235.9E-6)	CCATATCCAAGATTTGACCCGCAAC	CATTCCATAAGAACCTGAA	lead	AS	881180	881195
L200149(232.9E-6)	GCAGGCGTCACCCCCTATACATCAC	CTTGCGGTTTAGCAGAGAG	lead	AS	2219499	2219514
hsdR(232.7E-6)	GTTTGATCCACTAAGTCCACACGAT	CAACAATAAATAAAACATT	lead	AS	641728	641743
glgA(232.0E-6)	CATTATCTAGAAAATAATACTTCAC	ACCCTCTTTTTCTAGACTT	lead	AS	698612	698627
rdrB(229.4E-6)	TCTTTGCCGATTGGAATTGCTCCAC	CATGAACTCGATGCAATTT	lag	AS	1331986	1332001
qor(226.9E-6)	TTTGAACCTGAACGAGTAACATCAC	CGGCCCAAAACACTTTATC	lag	AS	723823	723838

pi148(226.7E-6)	CTTGGTGGAAGAATAATGACTTCAC	TCATGCAGCCAAAAAAGGG	lag	S	482353	482368
yjbB(225.2E-6)	AGGGAAGATAATTGCTAACCAGAAC	CATCCAACTGTGAAGAGAA	lead	AS	911052	911067
ps315(222.8E-6)	GAATACATTATACTTTTTACTTCAC	TCACAATAAAAGGAATGAC	lag	AS	2023936	2023951
yqgG(220.9E-6)	TCATAACCAATGGCATATCCACATA	CCCCAGCTAATATTAATCG	lag	AS	1664082	1664097
pi201(220.2E-6)	CTATAACAATATCTCTTATTCCAGC	ATCTGTTTTGGGCTTTTTG	lag	AS	1037038	1037053
intergenic(219.7E-6)	CTTCTATAAATTAAACATACTCATC	TATTCTACCTAAAATTCAG	lag	.	565296	565311
hslO(216.9E-6)	ACAAAAGGAGCAACTAACACTTCAC	CAGTTGAAGCTTTTTTATA	lag	AS	2034601	2034616
ptcC(216.4E-6)	CGGTCAATTACTCCCCTACTTGAT	AAGCAGTTGAAACTTTAGT	lead	AS	421115	421130
ycjM(216.3E-6)	GTTTTGTTCTCCCAACAAACTTCAC	CGAAAGAGAAATGAATTTG	lag	S	299901	299916
celB(212.9E-6)	ACCTCGATGCTGGAGCTTACTTCAC	AGTTATTATTATGGGTGCC	lag	S	178101	178116
ytcE(212.2E-6)	ATACACCGCGGTCAACAAGTGCATC	CATTTGGAAGTAAAATTTA	lag	AS	1929213	1929228
ftsH(211.5E-6)	CTATAGAAAGGAACTCCTGCTTCAC	CGGCAACAGCCTTAGCAAG	lead	AS	26613	26628
yljG(211.5E-6)	TTTGGCTGATAGACTTTTACATCTC	CTTCTGTTGAAAATTTATT	lead	AS	1197636	1197651
dltE(211.4E-6)	CGCTGGTTTTGCTAAACAACTTCAC	CAAATGGGCAAAGTAAACA	lead	S	144478	144493
topA(209.0E-6)	TCACAGTCTGGGTAACGATCACAAC	CATAGAAAATTCTATTTTT	lead	AS	1256515	1256530
citC(200.9E-6)	CATTACCAGCAATAGAACCCGTCGC	CAAAACTTCACCATTTTTC	lead	AS	1206875	1206890
mutS(197.6E-6)	TTTCTTGAACTTTCAATCACTTCAC	GCGTTTTTTCTTCCAACTT	lead	AS	1692486	1692501
rgpA(197.2E-6)	CCAGGATTTGTCCCCCTACCTCAT	GACCATGTAAATAAGCAAA	lead	AS	202268	202283
intergenic(194.6E-6)	ATCGTAAAACAAAAACTTACACTTT	CATGTTATCATAGTTAATA	lag	.	172797	172812
trxB1(194.5E-6)	CCAAAAGCTTTTTCAGTTACTTCAC	CCGTTTTAACGTTTTTATA	lead	AS	966106	966121
yveC(194.1E-6)	AAGTAACGACCTTCTGTAACAGGAC	CAGAAACGTAACCTACGAC	lag	AS	2143762	2143777
trmE(194.0E-6)	CGAACAATGGCAATTGCTCCTTCAC	CAAGCGGTGTCGAAATCGC	lag	AS	2327785	2327800
yvaA(192.3E-6)	AATTCAATTTGCATAAAAGCAATCG	CATCCAAACGATCTGGAAA	lag	AS	2100504	2100519
ptnC(191.7E-6)	ATAAGGTTGATAACCATTGCATAAC	CAACGGCAACAACCATCCC	lag	AS	1763862	1763877
yeaG(191.3E-6)	ATAACAAAAAAAGATGGTCATCAAC	CATCTTAAAATGAGTAAAT	lag	AS	407850	407865
ldh(189.0E-6)	TCTCATGCCTTGGCATTTACTTCAC	CTAAAAAGATTTACTCTGC	lag	S	1370008	1370023
ylcG(188.5E-6)	AGATTTCCTTTAACCAATACTCTAT	CACCAATGGTCACATCTTT	lead	AS	1131775	1131790
yciA(184.1E-6)	CCAATAAAGGATAATTCAACTTCAC	AAGTCCCTGCAAAAAGTGT	lead	AS	280687	280702
ytjF(183.8E-6)	AGATGAATATGAGCACTGACATAAC	CATATTTTCTGTGATGATG	lead	AS	1997999	1998014
intergenic(182.9E-6)	GGCTGATTAAGCACATTTTCATTTT	CATTTAGGACTGATTTTAT	lead	.	1880376	1880391
L200155(182.9E-6)	TAAACAGTTGCTTGGGCCTATTCAC	TGCGGCTGGCTTTTACACC	lead	AS	2342297	2342312
dpsA(182.1E-6)	TTATAAAGTAATTAACCTACTTCAG	CTTCAATCATCCAAAGTGT	lead	AS	2102154	2102169
pepN(180.9E-6)	TTAGTTGCAAAAGCACCTACTTCAA	CACCAGATTTTGTTTTTCC	lead	AS	302960	302975
yogI(180.4E-6)	CTCTTATCACTCGGGATTACTTCAC	TGATTCGACTGTTGTTAAC	lag	S	1467652	1467667
gyrA(179.5E-6)	GGGTGATATTTACCCATAACTTCAC	CGACAATACGTGCCGATTT	lead	AS	1121968	1121983
recX(177.9E-6)	AATGTATAATCACGTAATGCACTAC	TAATATCATCATATGAAAA	lag	AS	2260573	2260588
pspA(177.3E-6)	CATTGACCTGTCCTTTTCTCATAAT	TCCAGAAGAGCGATCAAAG	lag	AS	2304303	2304318
ycgD(176.4E-6)	TAGCGATTTGTTGATTTACCAAAAC	CAACATAGGCAATATTAAG	lag	AS	264664	264679
intergenic(174.5E-6)	TTGCTATATTTACTGTCATCACTTC	TCCTTTTCAAACAAAAATT	lead	.	1171031	1171046
pi149(174.1E-6)	AAAGTTACCTATTCAATGACTTCAC	GAATGGGTCCAAAAAGTTA	lag	S	482537	482552
noxB(173.9E-6)	CCACACAACCATGACTCAACTTCAC	GAAGTTGCAGCAGGACGTG	lag	S	841457	841472
hom(172.9E-6)	TCTGCCAAAGCACGCAATTCAGTTC	CATGAACAGCCAGTAAATC	lead	AS	1171374	1171389
ybjJ(172.0E-6)	GCATTGGCTTTTTTGCCTTCTTCAC	CAATATAAATCGCCACAAT	lag	AS	193714	193729
era(170.0E-6)	GAAATTGTAGCCGATTATACTTCAC	AAATGGAGTTTTCTGAAGT	lag	S	355476	355491
tra1077B(168.4E-6)	TTTTTAGCTACCGTCCCTACTTTTC	CTTTATATGAATTATGATT	lead	AS	139417	139432
yniJ(167.6E-6)	TCTTGTTCAAGGAGGGTTACATTTC	CACCTAAAAGCTGAAGATT	lag	AS	1389428	1389443
intergenic(167.1E-6)	CGTTTTTTCTTTCAATTGTTTAATC	CAAAAATCAGGAATTCCCA	lag	.	146664	146679
ykiF(165.9E-6)	ATTGAACGGTCATTTTCTTCATCTC	CAATAGCCATTACTTCATC	lag	AS	1086658	1086673
yqbK(165.8E-6)	ATTACATTAGGAAATAGTACTTCAC	ATAACCCAATTACCATCAA	lag	S	1621593	1621608
yuhI(164.7E-6)	TTAGCAATTTCAGCTAAATCATAAT	CATGAGCAGTGAATTTTCC	lag	AS	2077908	2077923
yrjA(163.1E-6)	TATAATGACGTTTCATTTCCTCTGC	CAATGCTAATTTATCTTTT	lag	AS	1790453	1790468
rheB(158.7E-6)	CAAGCAGTCATTACGGCTCCTTCAC	GTGAATTAGCTACTCAAAT	lag	S	416038	416053
vacB1(158.5E-6)	GCTTTTTTCATTTTTTCAACTTCAC	GTTCAGCATCAACCGCCCG	lead	AS	968864	968879
secA(156.8E-6)	CCAAGCACACGGCGGGCTGCTTCAC	GACAGACAGCAAATGCTTC	lead	AS	116558	116573
pi252(153.7E-6)	AAGGTAACTTACTCAATGACTTCAC	GAATGGGCCCCAATAGTTA	lag	S	1070851	1070866
ywfE(153.6E-6)	TGAGTTGCAGCATCTAGAACTTCAC	CGCGTTCAGGGTCAACAGA	lead	AS	2253509	2253524
kupA(153.2E-6)	GCAAAGCAGCACCACCAATCATTGC	CGGAATAATTAACCATTTT	lead	AS	609811	609826
adhE(152.2E-6)	CCGGCATCGAGAGCAGAACCACCAC	CAAGACAGATGACAGTGTC	lag	AS	2231413	2231428
intergenic(151.0E-6)	TCTAGTTAAGAAATCCCTATTTCAC	ATAAAATAAAAGACCGTTC	lag	.	767701	767716
carB(149.3E-6)	ATTGCATCGACTTCACATTCACGTC	CTTGTAAATAAGAATCAAC	lead	AS	1399233	1399248
rexB(148.9E-6)	TGCTCTGGAGTTAATTTTACTTCAC	TCATTTCGCCCTTCTCCTT	lead	AS	6410	6425
rexA(148.9E-6)	TGCTCTGGAGTTAATTTTACTTCAC	TCATTTCGCCCTTCTCCTT	lead	AS	6410	6425
L200139(147.0E-6)	TAGCTCAGTCGGTAGAGCACTTCAC	TCGTAACGAAGGGGTCACA	lead	S	155265	155280
ytcA(146.1E-6)	ATGGAATTCATCCCGCATACTTCAC	AATCATCTATGTTTTATAT	lag	S	1919841	1919856
ybfE(145.3E-6)	CTTGAGATTGTAAGCCCAATTGCGC	CCAGAAAATATCGGCGTCA	lag	AS	158953	158968
rplU(143.4E-6)	TGCTTACCATGTTTTTCGACTTCAC	CAACAACAGTTGCGCCTTC	lead	AS	1090794	1090809
nadR(143.2E-6)	TGAGCACCACGGTCATTATCATAAC	CAGAAACGACAAGCAAGAC	lead	AS	2067466	2067481
accC(141.8E-6)	GCAACTGTACCCACTCCTAATTCAC	GAGCTGCACGAATAATTCT	lead	AS	787362	787377
intergenic(141.1E-6)	CACAGATTCTCTCCATTTCCTAGAT	TAATAATCTAAAAACCATT	lag	.	1765147	1765162
pacA(140.5E-6)	TCATCAGTCATCAACTGTACATTAT	CAATATTCTGACGAACTTC	lead	AS	1149790	1149805
clpC(138.9E-6)	TTCATGGAAAAACATTCGACTTCAC	GCTTGATTGGAGCTCCTCC	lag	S	631578	631593
rpoC(138.0E-6)	ATTGTTTCTGGTTTTTTAACTTCAC	CGAATGACCAGTAACGAAT	lead	AS	1860730	1860745
yugD(136.4E-6)	CTTAACTCTTCTACAGAAACTTCAC	GCGCTAAAACTACGCGTTC	lead	AS	2069632	2069647
yseE(133.9E-6)	AGAAAAACAAAAACTCTCACTTCAC	AGCCTTTTGGCGTTAATTT	lead	S	1845334	1845349
ygiI(133.8E-6)	GAATGCATAATCGCACCAGCAGGAC	CAACGCCTTCTGATTTAAT	lead	AS	687818	687833
yvdB(133.8E-6)	CCAAGACCTTAAATCCAAGCACTTC	ACGGTAGAAATCTAAATTT	lead	AS	2132376	2132391
yrbA(133.2E-6)	CAGAAGTAATCGCATTAACACCATC	CAAAATTAAAGTTCCATCA	lag	AS	1710889	1710904
ldh(132.5E-6)	CGCGTTGTAGGTTCAGGTACTTCAC	TTGATACTGCACGTTTCCG	lag	S	1369738	1369753
yoiC(129.4E-6)	CGTGGACTGCAGCAGATAACTTCAC	AGGTGGAACAAGTTCTACT	lag	S	1486078	1486093
uxaC(128.3E-6)	AGTTGTAATTGTTGCACTCCTTCAC	CTTGAAAACAGCCAATCAT	lead	AS	1673326	1673341
pepF(128.1E-6)	AAGTATTCACTTGTTAAAACTTCAC	CGGCAGCATCTGCTTCATG	lead	AS	1783404	1783419
ytdF(126.8E-6)	GAAACTCCATTTTCAAAAACTTCAC	GTGCAAGATTAGCATCAAC	lead	AS	1937543	1937558
ymjE(126.6E-6)	TCTTGACGATTAGTTTTTACCCAAT	CATTTTTAGAGTTGAAATA	lead	AS	1296165	1296180
ypiA(125.6E-6)	ATTCGGAGTACCTCCATTAATTCAC	GAACCGCCCATTTTGTTGC	lead	AS	1580173	1580188
yvdD(125.2E-6)	GTTGCTTTCGGTAATATTACTTCAC	AAATTAATCCAGCTTTCAC	lag	S	2134298	2134313
noxB(125.0E-6)	TTTTGCATGAACAATGCGACTTCAC	GGTTTGGAATCATAAACTT	lead	AS	843000	843015
xylH(124.4E-6)	AAACATGCTGGAGCACCTACTTCAG	CAATTCATGTCATCTTTAA	lead	S	568323	568338
intergenic(121.8E-6)	AAATATAGTTAGAAAAACCTACTTC	CGGTTCCCAACTTGTTCTC	lag	.	1247526	1247541
yxcA(121.2E-6)	TAATGAGCCATTGTTTCAACTTCAC	CATAAATCAACATGAAGTCC	lead	AS	2323787	2323802
chiA(121.1E-6)	ACGGCATTGGGGTCTTTCACATAAC	CTGTTGCAGCAGCGTCATT	lag	AS	2027406	2027421

yngG(120.4E-6)	CCGCCTCGTTCGGAGAAGACACCAC	CAATACTTGTAAAGATTAG	lag	AS	1368216	1368231
ysjD(119.8E-6)	TTGTCAGCCACTTCACCTGCTTCAC	CAGATAATCCCAAAACTTT	lead	AS	1893729	1893744
yhdA(117.1E-6)	TATTGACTGGAGCCGATGCATTAC	TTGCCATACCAGCTGTGAT	lag	AS	731594	731609
secA(116.6E-6)	TGACGTTGCGCATAAATAACTTCAC	GTTGCTCACGGATGACATC	lead	AS	118226	118241
glpD(115.5E-6)	ATAAGATCCGTTACTGAACTTCAC	TCGTCCCATCGTGCCCAAA	lag	S	1271169	1271184
rpoB(114.1E-6)	TGTCATGTACATGATTAAACTTCAC	CACATGGTTGATGATAAAC	lag	S	1861379	1861394
arcC3(113.9E-6)	GGTAAGCATGCCACGATTACTTCAC	TTGAAAATGTGGAAAACTT	lag	S	1752051	1752066
nifS(113.8E-6)	GCGGACGGAACATTCCCAGCTTCAC	TTATAGAAGAAAACTTGCG	lead	S	1928168	1928183
dnaJ(113.8E-6)	TTCCAGCAGGAATTTTCAACTTCAC	ATTCCCATGAACAGTCGGA	lead	AS	2307464	2307479
rplD(113.0E-6)	ACTGAGTCGTTAAGTGTAACTTCAC	CAGCTTGTGAACCATCTTG	lead	AS	2169254	2169269
ygfC(112.4E-6)	TTTAAACAGGCATCATATACGCGAT	TGCGTTTTTCGTCAGATAG	lag	AS	654295	654310
tkt(112.3E-6)	AGCAAGGCCAGCCGTGTAACTTCAC	AAGAAGCTATCCAAGAATT	lag	S	1670095	1670110
glnA(111.5E-6)	CCTTTGATTCGCGTACCTGCTTCAC	GTGGTTTGTCAACTCGTGT	lag	S	2282652	2282667
ywfH(109.7E-6)	GCTCCACCCGGTGCCCCTACTACAC	TTGTATCACCAGGATGAAA	lead	AS	2259140	2259155
xynB(108.4E-6)	TTTAGAACATTGGAGCCTACTTCCA	AGTCCATTGTCGACAACCA	lag	S	1544744	1544759
yqeL(108.2E-6)	TCTGAGGATGTGATTACAACTTCAC	GTTTCCTGGGACAACTCT	lag	S	223614	223629
ygdA(108.1E-6)	ACTTTACGTGCAGAAGATACTTCAC	AAGATATGTATTCTTCAAT	lag	S	632597	632612
relA(107.1E-6)	TCTGCTGAATCACCAGCTTCTTCAC	GCAATTCCACAAGTTCATG	lead	AS	108310	108325
yfgG(106.7E-6)	CAACAATCATTGCCGGACTCATAAT	CAACATTGCCATTCCTGAT	lag	AS	564603	564618
ackA1(106.2E-6)	AAATATGGGGCTCATGGAACTTCAC	ACATGTATGTTTCACAAGA	lag	S	2090551	2090566
leuS(105.8E-6)	TTTGCGTCAGTTGTATTAACTTCAC	GTTCCCAGTCATAACTAAA	lead	AS	827793	827808
yrgH(105.8E-6)	TGTAAAAAATGAGCTAATTCTCGGT	CCCAATCATAGAAATCTTC	lag	AS	1769361	1769376
carB(105.4E-6)	CCACCAGCTAGTTTTTCAACTTCAC	GGACATAAAGTCCATTTTC	lead	AS	1398537	1398552
llrB(104.3E-6)	AGGGTCAAAATTGCCGATCATCAT	CAGCAATTAAAATTTTTTT	lag	AS	1458179	1458194
glk(103.8E-6)	ATGTCTTTAGAAGTCACTTCATCAC	CATTGTCAATAGCTGCTTT	lag	AS	2101334	2101349
yqaB(103.7E-6)	CTAAAATCAGGAGCCACACCATAAC	CAATATGACCACCCCATTC	lead	AS	1603759	1603774
hsdS(102.3E-6)	CCGTTTAGAAATGTTGCAACTTCAC	CCAACTTACGTTCTTCCCA	lead	AS	646044	646059
adhE(101.2E-6)	CGTACAATCTCAATTCCTTCTTCAC	GTGTTTCAGCTTTGTAGAT	lag	AS	2230834	2230849
snf(101.0E-6)	ATTATAATGAGAGAGCATACTCATC	CAGCGAACACCCGTTTCCT	lead	AS	2121892	2121907
ptcC(100.8E-6)	AACGTTGCGGCATCATTCACTTCAC	TTGTTCCTGCAGGAGCAAT	lag	S	421301	421316
potB(100.3E-6)	AACTGTAAAAAGTTATTAACTGAAC	CATATTTACCAAAATTCC	lead	AS	1177294	1177309
L200152(99.6E-6)	CCAGTGCGTCTTACCACACCTTCAC	CCTGCTCATGGATAGGTCA	lead	AS	2263088	2263103
ykjK(98.8E-6)	CAAGTCCAGCCTTACCAGTCGCAAC	CACACTACTTGTTGTGTTT	lead	AS	1100315	1100330
cpsM(98.4E-6)	ACATGACCGAAATTTTCTCCTTCAC	GTAAATCAACCCATGCGCC	lead	AS	198632	198647
yshA(98.2E-6)	TCTTTGGTGTAATTGCCTACTTCAT	GATGCGCAACTATAGAAAG	lag	S	1875454	1875469
rbfA(97.6E-6)	AGCTCATCAATTTTTGAACCATATT	CAACAGATTCATCTTTTGC	lead	AS	779168	779183
tuf(96.2E-6)	GAACCAACAGTACGTCCACCTTCAC	GGATAGAGAAAGTAGTACC	lead	AS	1929416	1929431
fadD(96.1E-6)	CCAGGCATACCAATCCCCATTTCAC	GGGCATTAGTGTCTGCTAA	lag	AS	655043	655058
yucG(96.1E-6)	TTAAAAGCGGCGGTCCACCCTAAAC	TTTGAGAATCTAATTGTCC	lag	AS	2028169	2028184
yhfC(95.8E-6)	GGTGAAACGATTGAAATACTTCAC	AATTTGGTCGTAAACGCGA	lag	S	755166	755181
yahA(95.6E-6)	AAGAACCAGCGTATTCAAACATTTC	CAAATCATTCATTTGGTCA	lag	AS	72409	72424
recN(94.5E-6)	CGCACAAAATCACTCGAAGCACGCC	CACCTAATAATAAAGACAT	lead	AS	883221	883236
relA(94.2E-6)	TTACTTGATTCGCGTTTAACTTCAC	CCTTCATCAGCTCTTCGGC	lead	AS	108916	108931
ycjM(93.0E-6)	TCTAACCAATCCTCTGCTTCTTCAC	TCACAACGGATATCAGCTG	lead	AS	300722	300737
dtpT(92.8E-6)	TGGTTCTTGGCAGATTCAACTTCAC	AAGCAATCAATGCTCAAAT	lag	S	705765	705780
ps311(92.7E-6)	AATTAGGCAAGCGTTCTGACTTCAC	AGCTAGTGAAGTTGTAGAA	lead	AS	2021054	2021069
nusA(92.4E-6)	TCGAAAACTTCATCCACGACTTCAC	GAGTCGAATAAACATTGAA	lead	AS	773978	773993
yvcC(92.4E-6)	TAACCTGTAGGCACCTCCTCTTCAC	TTATGATATAAAGATTACC	lead	AS	2124348	2124363
pfl(91.4E-6)	GTCATTAATGGAGCTGCAACTTCAC	TTGGACGTGTTCCAATCGT	lag	S	658237	658252
rpe(91.0E-6)	TTTTCAATATTTTTCTCAACTTCAC	CATTAAAAATAAATGACCC	lead	AS	2003231	2003246
citF(90.5E-6)	TATCAAAATCAATCTTTAACTTCAC	GAATGATATCAAGCAAGCT	lead	AS	1210497	1210512
menE(90.0E-6)	TCTGCCTTAGCAACAAGTGCACATT	CCTTGATTTCAGGAAGAGA	lag	AS	733083	733098
usp45(89.7E-6)	TCTCAAGCTCAAGAGTTGACTTCAC	AACAAGCTGAACTTAAAGT	lag	S	2313993	2314008
yxfC(88.4E-6)	ACCGCATCTACATAGGTTTCATAAT	CATGAGCTCGATTAATCAA	lead	AS	2359357	2359372
leuC(87.1E-6)	GTCCGAACACGACGTCCTGCTTCAC	GTAAGCCCTGAAATGCTTG	lead	AS	1242999	1243014
yeeG(87.0E-6)	TTCATGAGTGCTGCAATGACTTCAC	GTTGGTGAGCTGCCCGACC	lead	AS	446315	446330
ptnD(86.8E-6)	TCGATATCAGCACCGTTAGCACGTT	CTTCTTCAAGGGCAAGAGT	lag	AS	1764360	1764375
ywaF(84.5E-6)	CGTTCTTGATGTTTCATTGCACGAC	CAATTCCTGAATGACTCAA	lag	AS	2205732	2205747
guaA(84.3E-6)	GGTTTCCACGTTGTCGGAACTTCAC	CAAACAGTCCATTTGCTGC	lag	S	1517292	1517307
yeeF(83.5E-6)	TCAGAGATTAAAATTCCTACTTCAT	CAATGGTCAAAAAATTACT	lead	AS	445084	445099
hasC(83.5E-6)	TTGTAAAAGGCCTTTAGAAACTTCAC	CGTTAGGGTCAATGACACC	lag	AS	1378400	1378415
lacZ(83.0E-6)	TTAGGATCAATATGTTCAACTTCAC	TAACACCAATATCATTATC	lead	AS	2055873	2055888
yjdE(82.2E-6)	GAACATCTTGAAGGTAAAGAGGAAG	CATCATTTCAACACCCATC	lead	AS	933438	933453
atpH(81.8E-6)	ATGAAGTAACAGTAATTAACACAGT	CAATGAAAAAATTCTCGGT	lag	S	1827415	1827430
thrS(81.6E-6)	TTTCATCATCTGGGAAATACTTCAC	TGTATCTTTAGGGTCACGG	lead	AS	1987780	1987795
malE(80.4E-6)	TACCTTGGTAAGACTCCTGCTTCAC	AATATTGACAGTCAACTTAA	lead	S	1740226	1740241
zwf(80.1E-6)	TTGATGACTCTAAAACAACTTCAC	GATAATACTCGTTTGTCCA	lag	AS	2301470	2301485
bglH(79.8E-6)	TCCCGCATTTACGAATCGACTTCAC	CGTAAACACGGAGTAAATG	lag	S	1490018	1490033
noxE(79.7E-6)	CTTTTTGATGCAGAAAGTACTTCAC	TTGCTTCATATTATGATGA	lag	S	397135	397150
ptbA(79.5E-6)	ATTTTAATCGCGCCTGAAACTTCAC	CTGTAAGGGTAATATTAGA	lead	AS	1491561	1491576
tenA(79.5E-6)	GCGATTTGGACGGTAGCTTCTTCAC	TCGCATAGGTTTCAATCCA	lead	AS	1838849	1838864
yeeA(79.4E-6)	TGAACAAATTGCGATTGTTCATCAC	CAATTAAGCCGGACTGCCA	lead	AS	440760	440775
yeeA(79.1E-6)	ACTGTTCACGGTTCAACGACTTCAC	GTCCGGTTTTTGCTGGGAT	lag	S	441510	441525
cpo(79.1E-6)	ATTTGGCAAACCATGTAACTTCAC	TGGACGAATAGCCACCAAT	lead	AS	834569	834584
vacB2(78.8E-6)	CTTCTTTAGATGAAGAAGCCTACTC	ACGCGCAACATCAGTCTAT	lag	S	1226984	1226999
yyaL(78.6E-6)	ATATTATCAGGTTCGCCTACTTCAT	CTTCTGAAACATTAGCTAC	lead	AS	11749	11764
yljF(78.4E-6)	AAAGCCGAAACATTCCTGCGGGAC	CAAATCCAATGACTACAGG	lead	AS	1195835	1195850
intergenic(77.3E-6)	AGCTTGGACTAGAAAAATACTTCAC	AAAATGCTATACTAGGTAA	lag	.	1980330	1980345
intergenic(76.6E-6)	ATAGACGAATTTCTTTTTTCTTGTC	CATTGGAACTCCTTTTAAA	lead	.	1293417	1293432
ymjE(76.0E-6)	AAGCTCCGATCATCACTTGCATCAT	CAATAACAGTTTACCACTAG	lead	AS	1297164	1297179
cysS(74.5E-6)	GAATGTTCAGTCATGTCAACTTCAC	TATTAGGCGAAACAATTGA	lag	S	1918936	1918951
oppD(74.2E-6)	TCCCCAACAATAGCTAAAACTTCAC	CACGTCTAAGACTTAAATC	lead	AS	1910699	1910714
intergenic(74.0E-6)	AAATAAAATGGAGTAATGACTTCAC	AGCGCACACTTTGAAAAAA	lag	.	1830442	1830457
hom(73.8E-6)	ATTCATAAAACAGCATTCATAAC	ACCATTGACACTGGCCAAT	lead	AS	1171877	1171892
intergenic(73.7E-6)	TGGGGAGTTTGAAATACTCCTTCAC	GGATGATATGAATTAAAAA	lead	.	47610	47625
trpS(73.5E-6)	AGACCGTCCAACAGGAAAACTTCAC	ATCGGCCACTATGTAGGCT	lead	S	68223	68238
serS(73.0E-6)	AGAGCGTAAAGTTCAACTCCTTCAC	ACTTTGAATGGTTCTGGAC	lead	S	1768329	1768344
kinC(72.8E-6)	GAGCGAAATGGATTCATTCCCAGAA	TCATGTGTCTCAATAGTTA	lag	AS	402440	402455
dnaK(72.4E-6)	AGCCGGTGCTGCTGGCCCTCTTCAC	TTGGAAATGGCTTTGACTC	lag	S	979257	979272
nusA(72.2E-6)	GTTGGAATTTCTTCCCCATCTTCAC	CCACGAATTCATAATCATC	lead	AS	774845	774860

amyY(72.1E-6)	GCCGAAGCCGTTGAAAAACTTCAC	CCACTAAATAAGCTTTTGG	lead	AS	1733687	1733702
ptcB(71.4E-6)	ATTTCAGCTTTTTTAGCAACTTCAC	CTTTCATGAAGGCAACTTG	lead	AS	419068	419083
yqfG(71.2E-6)	TTTTCTGATATATTATTTACTTCAC	TTGGTTTGGACGAAATAGT	lead	AS	1659783	1659798
ptsI(71.1E-6)	TCAAATACAGATGCTGCTTCTTCAC	CAAGGGTTGTTACTGCTTT	lead	AS	120832	120847
tra904I(71.0E-6)	ATTGTAGGCTGGTCAATGTCTTCAC	GGATGCAAGATAAACTGGT	lag	S	2215294	2215309
yciH(70.9E-6)	CTGGTGACTTCAAGTTTGACTTCAC	ACCAGTGGGTGAACCTGCT	lead	S	290558	290573
purB(70.9E-6)	TTAGCAAAGAATTCTTCAACTTCAC	GTTGTTCAGATTTTTGTAA	lead	AS	1688986	1689001
pgk(70.5E-6)	CAAGGTTACGAAATCGGAACTTCAC	TCGTTGAAGATGACAAACT	lag	S	243525	243540
glyQ(69.6E-6)	CTCATTGTTCCTGCTCCTACTTCAT	TATCATAAGCTTGCATCAA	lead	AS	1101918	1101933
ymdC(68.5E-6)	TGAGAAATTTCCTGAAGCACTTCAC	AATACGCTAAAAGATGCAA	lag	S	1234116	1234131
yliB(68.0E-6)	GATTTGATTTTTCCAAATACTTCAC	AGATAATCTATGGATTCCC	lag	S	1181868	1181883
ilvC(67.6E-6)	TTAGCTACCGCTTCTCCTACTTCAA	ATGTTTCAAAGCCATCTTC	lead	AS	1249913	1249928
intergenic(65.9E-6)	TCATTACTTCTCCATCCTTCTTCAC	TAATGAAGGCTTTTTAATT	lead	.	297420	297435
ylgC(65.6E-6)	GTTGTTTTAAAGCTTCATAGACAAT	CATACAAGCACAATTTGAT	lag	AS	1162306	1162321
racD(65.6E-6)	GTACAACCTAAAATGGTTACTTCAC	AATTCATTTCTGACGCGAC	lead	AS	2309368	2309383
ypgB(64.6E-6)	TGGCCGCTTTCATCACAAACTTCAC	TCCATCTACAGCCATTTTC	lead	AS	1562082	1562097
trpB(64.3E-6)	GATTGTCTTTTAACGTCTTCTTCAC	CCATATAAATCGTACATTC	lead	AS	1495507	1495522
pycA(64.1E-6)	ATGTTATTGCTAATGAAAACTTCAC	AAGTGGTCAAGCAACAACA	lag	S	664950	664965
pepN(63.9E-6)	GGGTCAACAAGCATGCAGACTTCAC	GATAAGTGATACATCCCCA	lead	AS	303152	303167
ccpA(63.7E-6)	GTCTTTTCCTTTACATTTGCATTTC	CATTTACAACACGACTAAC	lead	AS	1696846	1696861
yfjF(63.5E-6)	GATTTTTCAAATCTAAAAACTTCAC	TGCAGCGAATATTGGAATC	lead	S	598934	598949
polC(63.5E-6)	TTGATTGCTGCATCAAAAACTTCAC	CAATTTCGCAGGCCGAACC	lead	AS	2191543	2191558
yqgC(62.4E-6)	AACCATTTCAGCTCTTCAACTTCAC	TCATTTGTGGTCTTAAACT	lead	AS	1666094	1666109
pydA(62.2E-6)	TAAAAGAAGTTTTTACCTTCTTCAC	AAAGCCTTTAGGTGTAAAA	lag	S	1593286	1593301
ilvD(62.1E-6)	CCTACTTAAAGAAGGAAAACTTCAC	GGTGATTGTTTGACCGTCA	lag	S	1246820	1246835
gyrA(61.6E-6)	TCATCTTCAAGAGTAAGAACTTCAC	CAACTAACAATTCTGTTCG	lead	AS	1123222	1123237
ykiG(61.5E-6)	CAGGTTTATTTTCATGCTTCTTCAC	GCTCAATGGAAGTTTTACT	lead	S	1088003	1088018
yjgE(60.7E-6)	TTTAAAATTACTTCTTGAACTTCAC	CAACTAATTCAGGGTCAAG	lead	AS	964786	964801
trpE(60.7E-6)	GGACGAAGCTCGCCGGTCACTTCAC	TTGTAATATGCATGACGTA	lead	AS	1500446	1500461
rbsK(60.2E-6)	CCATCGTGGAAAATGACTCCTTCAC	TTCCTAAGGTGACAATCAG	lead	AS	1686674	1686689
ylbB(60.0E-6)	CGATAAATATAAAGTGTGACTTCAC	GGTCATAGAATCCTAAAAC	lead	AS	1113813	1113828
ydbA(59.9E-6)	TGATTTCCTTCTTCGATAACTTCAC	CATCTTTAAGAACAACAAT	lead	AS	312783	312798
ylgG(59.9E-6)	CAGATAAAGCTTGAATTTCAGCAAT	TAGGTCTTCATTCATTTTT	lead	AS	1168308	1168323
rplC(59.8E-6)	GCAGGTTTGTTGGTCAAAACTTCAC	GAAGTGTATCGAAACCAAC	lead	AS	2169777	2169792
ynhC(59.5E-6)	TCCATTTTAGAAAGTTCAACTTCAC	CTTGTTGACGTTTTTGACG	lead	AS	1374723	1374738
pepN(59.1E-6)	GCAATAACAGTTGCTACATATTGTT	TGCTTTGAATGGTTGCATT	lead	AS	303201	303216
aroB(59.1E-6)	TTGATGACCTCCCCCAGTCCTTCAC	GGAATTCTCTTTGACCGAG	lead	AS	1814295	1814310
glmU(59.0E-6)	GTTTCTTCACCAATCGTAACTTCAC	TGTCAATATAAGTTGTCGC	lead	AS	1951674	1951689
hsdR(58.9E-6)	CTTTGATAAACTTCTTTTTCATAAT	CTTTTTCGGAATGTTTTTC	lead	AS	642226	642241
accA(58.9E-6)	GCAATATTTTTTTGTGAAACTTCAC	CTGTCAGTGAATCAATTGT	lead	AS	789638	789653
yshA(58.7E-6)	ATTTTTTTGAATTTCTTGACTTCAC	AAGGTGGAGCAAGTCAATT	lag	S	1875797	1875812
rplJ(58.6E-6)	ACTTTGAATTCAACGCCTGCTTCAC	GAAGTTGTTTACGAAGTTG	lead	AS	1301775	1301790
leuS(57.9E-6)	TTATCCATCTGGTGCAGGACTTCAC	GTTGGACACCCAGAAGGTT	lag	S	827588	827603
ywjB(57.7E-6)	ATGCAGGCTTACAAAAAGACTTCAC	AGTCTGCTCAAGTCATTGA	lag	S	2296014	2296029
era(57.3E-6)	TTGAACGTGGTTCATCAGCCGCAAC	CATGAACAAAACGACATCA	lead	AS	355313	355328
papL(57.2E-6)	AGGATTTAAAACGTCGTGACTTCAC	GATCAATGCCTTTGCTTGT	lag	S	1602930	1602945
gyrA(57.1E-6)	GGCGACTATGTTGTCGGAACTTCAC	GAATTGATGACAGTCAAGA	lag	S	1123879	1123894
ylcF(56.7E-6)	AGTAAAAAATTATATAATTCTTCAT	TCATTTTGTTTTTCCCTTT	lead	AS	1128157	1128172
ylfB(56.2E-6)	AATTTCAAAGATGAGACGCCACATT	GATGAAAAGGATTGGCAAC	lead	AS	1152026	1152041
ilvH(56.1E-6)	GATACTTTAATCAAGACGACTTCAC	GTTCTACATGAGGCAAATC	lead	AS	1249451	1249466
pstE(54.4E-6)	TTTTGGTCTTGACCTCCTACTTCTT	TCCAGTTGGTAATTTTACC	lead	AS	1775284	1775299
citE(54.3E-6)	CCCTTGACAAAGCCTTCTTCATCAT	CAACATTGCCAAAGACCGT	lead	AS	1208681	1208696
uvrA(54.3E-6)	TAACGTCTGCCATGGCAAACTTCAC	ATGGAACATAAACATCCGG	lead	AS	1886165	1886180
Intergenic(54.2E-6)	TGCTGACAGAAGCCACATACATGGT	TAGAGTACTATTTAGTTTC	lag	.	165032	165047
pdp(54.1E-6)	GAATAAAGCTGAGCAATGACTTCAC	CGGCACTCACCTTATCTCC	lead	AS	1464555	1464570
valS(54.1E-6)	CCTTCTCCCTCAGGTGCTTCTTCAC	CAACATACATTTCACCCGC	lead	AS	2249627	2249642
ytgG(53.6E-6)	TTTTGATTGACTGATAAATCTCATC	CCCTTCTTCAAATGGAACA	lead	AS	1969240	1969255
aspC(53.4E-6)	GCTCAATCAGTCGTACAAACTTCAC	TTGGTGGCACTCAATCGGT	lag	S	163533	163548
yhdB(53.2E-6)	GGAGTCGTGAAAGCAACAACTTCAC	CAAATCCCGAAAGTCCGTC	lag	AS	732748	732763
L200158(52.9E-6)	AGCGCTTATCCCTTCCCTACATAGC	TATCCAGCCGTGCTCCTGG	lead	AS	2360508	2360523
intergenic(52.5E-6)	GGAAATCCTGTTTGACCTATTTCAC	GACTTAATCACACAGAACA	lag	.	1041037	1041052
frdC(52.4E-6)	TGATTTACAAGAATCGCCCCTTCAC	CACGAATTGATTCTGAAAC	lead	AS	1138793	1138808
nrdG(51.7E-6)	TAAAGTGAGCAGCGCACCCCTTCAC	CATCAACGAAATTGAAGGG	lead	AS	272742	272757
bcaT(51.4E-6)	CCTAAATCTTTGGCATAAACTTCAC	CCTCAATCGCTTTGAGTCC	lead	AS	1322017	1322032
apl(51.0E-6)	GTGGGAAGATGTTCTCAATCATAAT	GAGAAAGGCAACACCAAAG	lag	AS	719594	719609
ytbA(50.9E-6)	TCAATCAGCTGGTCAAGAACTTCAC	TGACTAATTGGGCAGTTAA	lag	AS	1913156	1913171
copA(50.6E-6)	TGAACAACAACCGCTTTAACTTCAC	CATCTAAATTAATTGTTCC	lead	AS	846827	846842
ywfF(50.2E-6)	GTCACAAAAATAATTTGTACTTCAC	CTGTTTTTTGTGAACGACG	lead	AS	2255346	2255361
yebB(50.1E-6)	GAAGAGTGTATTTTACGAACTTCAC	TGAAACCAGCAGCTAGTTC	lead	AS	411411	411426
recN(50.1E-6)	ATAATATTCGATTTATTTGCTCAAT	AAACGTTTAGCCTGAGCTA	lead	AS	884734	884749
ybcG(49.8E-6)	GTGCATCATTAACAGCATCAGTAAC	CATATCTTGTAAAGTTTCA	lag	AS	122935	122950
yibB(49.8E-6)	TGACTTGAAAGGAAAGTAAAGCGTT	CATTGACTTCATCAAATTG	lead	AS	813101	813116
pi229(49.6E-6)	ACTAAACCAGAAGAAAAAACTTCAC	CGTTTGCTCTATTTATGAG	lag	S	1052899	1052914
ypdB(49.1E-6)	TCAGCAAGATATTTTCCTACTTCAT	TCGCTTTTTCAAAACGTGT	lead	AS	1532507	1532522
fusA(48.9E-6)	ACACACCAGGTCACGTGGACTTCAC	AATTGAAGTACAACGTTCA	lag	S	2354018	2354033
ydbA(48.0E-6)	TTGATAAAAGTTGGAAACCATTCAT	CAATGGGAAAATCATACCA	lead	AS	311876	311891
intergenic(47.3E-6)	AAAAGATTAGCAAGGGGAACTTCAC	AGATTGGGGTCCTATTGTC	lag	.	295163	295178
ysiE(47.3E-6)	TATAATAAGAGTATGATTACTTCAC	TTACGATTCTTTCAAGTCT	lag	S	1889135	1889150
pflA(46.7E-6)	ACGTGACGAACCCACATTGCATTAC	CACGGTCTGATAAATACTG	lead	AS	1880728	1880743
yoiC(46.6E-6)	TTCACAGCAGCAGACCCATCTTCAC	AAATCGCAACAAATCTTGC	lag	S	1486532	1486547
apt(46.5E-6)	TCGTAAGTTGCTTCAATGACTTCAC	GAGGTAATTTACCAGGCTT	lead	AS	623066	623081
intergenic(46.3E-6)	ATCAAATATAGCTGCTGTACTTCAC	CAATATTTCTGTGCAGTTT	lag	.	1413891	1413906
ypjA(45.7E-6)	ATAATTTTTTGAATCGCTTCTTCAC	CACGTTCATCAATAATATC	lead	AS	1590916	1590931
tuf(45.7E-6)	TCTTTGCTCAATACGTAAACTTCAC	CTTCGAAAAGTTTGTGTGG	lead	AS	1929629	1929644
galK(44.7E-6)	AATAGGATTGCTTTTTTGACTTCAC	CAAAACCAATAGCGAATTG	lead	AS	2061130	2061145
yhcH(44.1E-6)	CCAAATAAAAAACCACTCACATGAC	CAACAAAGACCTGAACATC	lead	AS	727005	727020
optC(43.3E-6)	TGGAAAATTTAAATAAAGACTTCAC	CCTTGTTGGTTCAAAGGGT	lag	S	347970	347985
yldA(42.9E-6)	AGCACCTGAACTCTTTTTACATGAC	GGATTAAGTGTTTTAGCCT	lag	S	1132657	1132672
yliB(42.9E-6)	ATATTTCTTTCTTATCCTACTTCAG	ATTTGCAGATAGGAAATGC	lag	S	1182872	1182887
ydiA(42.7E-6)	GAGAAGTCTATGATGTATTCAAAAT	TCATATCATTTTTAAAACA	lag	S	381887	381902

ykjB(42.6E-6)	AATAGCCGGACGTTTGCCTCTTCAC	CAACAGTGGCAGGGATGTC	lag	S	1092491	1092506
malF(42.3E-6)	AACCACCAAATAATAATGACTTCAC	TGGCAAGAAGGAGAAGCCC	lag	AS	1740489	1740504
pi246(42.1E-6)	TTACGTAGAAATAAGCATACCCATC	AGACATAACTTTAATCGCA	lead	AS	1068667	1068682
pdhC(41.9E-6)	ATATCTCCTTCATGCATTCCTTCAC	CAATATCAGGCATCGTGAA	lag	AS	61832	61847
dexA(41.7E-6)	CTTTCCCAATCAAGTCAATCACATC	CATGCGAAAACCGCCGATT	lead	AS	1736112	1736127
carA(41.1E-6)	TTGCAACACGGACGAATTGACTTCAC	CTCACAAAATCATGGATAT	lag	S	1644318	1644333
gltD(40.6E-6)	GCAAATCGAATTCCAACTGCTTCAC	GGCCTGAAATATTTAAATC	lead	AS	1315740	1315755
rpoB(40.6E-6)	CGAAGTGATGTATCACGAACTTCAC	GAGCTTTTTCACCAAAGAT	lead	AS	1861909	1861924
yfgG(40.5E-6)	GCACCCCAGAGATAATAACCAAGAC	CACACTTGGATGTCCTAAA	lag	AS	564702	564717
yugD(40.5E-6)	ATTGAAAATTCTTCAGTGACTTCAC	CAGTAATCGATTTTCTCTC	lead	AS	2069410	2069425
yljB(40.2E-6)	GTACAATCCAAGGCGATGACTTCAC	AAGAAGTGGCTACTAACTC	lag	AS	1192125	1192140
leuC(40.2E-6)	GTTGCAAGAACATGTTCAACTTCAC	TTGTACCAATTCCAAAAGC	lead	AS	1243302	1243317
relA(39.4E-6)	CCTCTTGGTAATTCTTGGACTTCAC	CATTTGGAGTGAAGACATA	lead	AS	108400	108415
yneH(39.2E-6)	TCCGAAACAATAATTTCTTCAAAAC	CTTTATTTGAAAGCTCTAA	lead	AS	1346233	1346248
bglR(38.8E-6)	ATTTTTTTGTCTTCAACAACTTCAC	CTGCTTTTTTTCCCAAATCC	lead	AS	1493676	1493691
gntK(38.8E-6)	ATATTTAGGTCACCTACTACTTCAA	CAAGGCTAAGCGCAACCAT	lead	AS	2268969	2268984
thiL(38.5E-6)	CCAGCGATTACAACTTCTGCTTCAC	CAAGCTGTAAAAGTTGTTT	lead	AS	1612822	1612837
noxE(38.2E-6)	ATTCTAATGCTTTACAAGACTTCAC	CTACATTGCCCTTGCCTCA	lag	S	397469	397484
yjhD(38.2E-6)	TGTCCAATCTCGTTACCTACTTCAA	CAAGTTTTCCTGCTTTTTT	lead	AS	972867	972882
dexB(38.2E-6)	TTTGTAAAACCAGCTTTCTCATCAC	TGGTCCATTGCATTGGCGT	lead	AS	1525229	1525244
fbaA(38.2E-6)	TCGATAGGAGCAAGTTCACCTTCAC	CAACGATACCGTCTTCTTC	lead	AS	1979853	1979868
yjgD(37.8E-6)	ATTGGACTTTTGAAATCAACTTCAC	TTGCCTTCGTTGCAGGTGT	lag	S	964028	964043
parC(37.8E-6)	TTGGCAAATTTTTTCTTAACTTCAC	GAAGTTCGCGTTTCATTAA	lead	AS	1012281	1012296
intergenic(37.4E-6)	TATGTTCGAAAAAATTCGACTTCAC	AAGGCCTAAAAAAGGAAGA	lag	.	1212453	1212468
guaA(37.2E-6)	CGAATACGGTGTTGCCCACTTCAA	CTTACTGGAAAATCAGCAT	lag	S	1517407	1517422
yqgA(37.0E-6)	GGTTTTGTTTATGTTAAAACTTCAC	GTGATCTGATGCGTGAAGC	lag	S	1662192	1662207
butB(36.9E-6)	ACTTTAAGTCCGCTTACTCCTTCAC	CTATAAAATTTACAACCCC	lead	AS	917656	917671
rsuA(36.7E-6)	TTTTTCAATAGAATTTTGACTTCAC	TACGGCTTCCGAGGCCACT	lead	AS	2326760	2326775
yniH(36.6E-6)	GAACCAGCAGCCTCAAGACCACGAG	CAACAGCTCCTTGGACAAA	lag	AS	1387665	1387680
yvcC(36.2E-6)	CAAGGCTGGAATTCTTTTTCTTCAC	AGTGGGATGGAGTCAATAG	lag	S	2127492	2127507
tra983C(36.1E-6)	TCAATCTCGTAATGCCCACTTCTA	AGCGATTGTTAATGAACTC	lead	AS	958140	958155
ymcA(36.1E-6)	ACATTAAAACTTGCTCCTACTTCTG	ATCAAGGGAAGGTTGCGGC	lag	S	1220304	1220319
pi146(35.8E-6)	AAGGTTCCGGATGTTCCTACTCCAC	TCGCTCGAGTGATATCAGT	lead	AS	481528	481543
rpsG(35.4E-6)	TACATTGGATCTGCCAAAACTTCAC	GTTTTGGGGCACGATTTTT	lead	AS	2355006	2355021
ybeF(35.2E-6)	ATATTTATTTCCCAAATCACTTCAC	TAGGATTTGGAGTTTTATC	lead	AS	149869	149884
ybeH(34.8E-6)	TCATTATGTCATTTTTCCCCTTCAC	CACGGGTTGGGTCGGAAAA	lead	S	142965	142980
apl(34.0E-6)	CGACAGAAGAAGATTGTTGCATATC	CTCTCTTTAAAAACCATTT	lag	AS	719326	719341
trmE(34.0E-6)	CAAGCGGTGTCGAAATCGCCGCAAT	CGTATCAAATTCTTGTGTT	lag	AS	2327760	2327775
accA(33.4E-6)	AATGCTCCTCCTGAACCACCTTCAC	CTGTGATGAAAGCAATAAT	lead	AS	790082	790097
yudI(33.3E-6)	AAAAATGGAAATTGCAAAACTTCAC	TTGGCACGTCTCGTCGAAT	lead	S	2036707	2036722
ctsR(33.2E-6)	GACGAGCAGAATATTGATACTTCAC	AATTTTGATGTATCCACCG	lead	AS	629551	629566
infB(33.1E-6)	TCTTGAACATTCATTCCTACTTCAT	ATTCAAGACTTTCTGGTAA	lead	AS	776847	776862
fusA(32.7E-6)	TATCTCTGGTATGGGTGAACTTCAC	TTGGACGTTCTTGTTGACC	lag	S	2352871	2352886
ybaI(32.6E-6)	CCAGCAAGTAAAGCTGCTTCTTTAC	CAAAATTTCTTGAGAAACT	lag	AS	110171	110186
vacB1(31.9E-6)	ACTTCCAAAACGTCAATTCCTTGGT	CATCTGCTTGACCAATAAT	lead	AS	967655	967670
pi214(31.8E-6)	ATAATCTTGCAACCATGGACTTCAC	AGGCTCCAAGTTCCCCACC	lead	AS	1044606	1044621
pepDB(31.4E-6)	ACAAGTGTTACAAGGTCTTCTTCAC	CCATCCCATTTTTCCACATA	lead	AS	1601634	1601649
yafB(31.3E-6)	ACACTTGGGTAAATGGCTTCTTCAC	CAACCATGCTAATAAGTCC	lag	AS	51093	51108
intergenic(30.6E-6)	ATTATCAAATTAAAAAGCCAAATTG	GCTTTTTTATCTGCTCAGC	lag	.	420472	420487
pflA(30.5E-6)	AGCGGATATTTCCAACCTAATTCAC	GCCATTTAAATTCACCCAT	lead	AS	1880578	1880593
pi146(30.4E-6)	ACACCAACAACTGGCTGGACTTCAC	AAGTCCCTACTCTAGTCAA	lag	S	479371	479386
hrcA(30.3E-6)	GATTCAAAGAGTTGGAGAACTTCAC	TCGTAACTTTGAAATAACG	lead	AS	977393	977408
yxbD(30.3E-6)	GCAGGTCACGTTGTCCCTGCTTCAC	GTCGTGGTGAAGGAATTGG	lead	AS	2319286	2319301
truB(30.1E-6)	GTTGAAAAACCAATAGTAACTTCAC	CTTCGTAAATTTTACCTGC	lead	AS	1140585	1140600
yrgI(29.9E-6)	GAAATATTACGATTTTGAACTTCAC	GTAAGGATTCTCCGTCAGC	lag	AS	1769961	1769976
feoB(29.3E-6)	GCTCGTAAAGTCCAACGAACATCGC	CCATTTCTTTATAAATTGT	lag	AS	190202	190217
ptnD(29.3E-6)	GCTGGAATGAGCGAGTAAGCCCAAC	CACCATTTTGCATACGTTC	lag	AS	1764210	1764225
ycfF(28.8E-6)	GCTGAATGAAGAAAACGACCATTTC	CAGGAACATTTGTTAATAT	lag	AS	257260	257275
L200150(28.6E-6)	GGAATTCCACTCTCCTCTCCTGCAC	TCAAGTCTACCAGTTTCCA	lead	AS	2222501	2222516
yleF(28.4E-6)	AATGATTCTGAATCACCTACTTCAA	TTAAGAATAAACTCAAAGT	lead	AS	1147802	1147817
ychC(28.2E-6)	AAATCATGCCAATATTTAACTTCAC	ATAGAACAAGTAGTGTTTT	lag	AS	273862	273877
L200064(28.1E-6)	AGAGTAGTTTGTATCGAAACTTCAC	ATCCCACATTGCCAATTTA	lag	AS	1718135	1718150
glyA(27.5E-6)	GTTGCTGGCGCTTCTGCCTATTCAC	GCCTGATTGATTTTGCTAA	lag	S	591480	591495
ywaF(27.4E-6)	CTATTTAAATGGAGGATATCGTAAT	CATCCTCAGGATTTTTTGT	lag	AS	2205804	2205819
uvrC(27.3E-6)	AATTCAAATATTATGGGGACTTCAC	CAGTATCTGCAATGGTTGT	lag	S	857817	857832
mapA(27.3E-6)	TAACGACACAGAAGATGGACTTCAC	ATCACATCAATGACTGGCT	lag	S	1729190	1729205
argG(27.1E-6)	TTGGCATATTCGATTTCTTCTTCAC	GAGCCCAATGCCATTCACG	lead	AS	127192	127207
recD(27.0E-6)	AAATGAAGAACTTTATCTCCTTCAC	GAAATTTCATATCACCCAA	lead	AS	1797764	1797779
ykhD(26.9E-6)	AGCACTTTCGTTTCATAGCCGTATC	CACGTCGGCCTAATTCGCC	lag	AS	1072513	1072528
chiA(26.9E-6)	GTGGCAGTAGAAGCAGTCACATTAC	TAATTGAAATTGTGGGCAA	lag	AS	2027631	2027646
glmM(26.8E-6)	TTTTGTGGATAAATCGTAACTTCAC	TTGCTAATTCAGATAAAGA	lead	AS	436154	436169
intergenic(26.4E-6)	TCACCTTCTATAAATTAAACATACT	CATCTATTCTACCTAAAAT	lag	.	565292	565307
ftsK(26.4E-6)	CCAACTTGACTTTCATCTACCTCAC	CAGGGTCAAAGCTTTCGTC	lead	AS	1704506	1704521
ywcC(26.4E-6)	GCTGCGGTTCAAAACCCTACTTATA	TCACTCTCGATGAAAAGGA	lead	S	2228600	2228615
ps311(26.3E-6)	AAAGATATAATTTCTACAACTTCAC	TAGCTGTGAAGTCAGAACG	lag	S	2021044	2021059
yiiI(26.1E-6)	ATACGGAAACTTTAGCGCCAGAAGT	TCTAGCAAGTAAGTTTAAA	lag	S	889057	889072
typA(25.8E-6)	GAAACTTGAAGTTCATATCCTTCAC	GGCGCATTGTTTCAATAAG	lead	AS	2093373	2093388
acmC(25.7E-6)	GCATAAGTGGGGTCTGTTGCATAGC	CAGAATTTTGAACGGCTTT	lag	AS	1402971	1402986
yjcE(25.3E-6)	TCTCTCCACTCAATCTTAACTTCAC	GAAATAATAGCCGTAATTG	lead	AS	926233	926248
als(25.3E-6)	CTGGAATCCCAAATACATACTTCAC	TTTATGGTTAATCAAACTA	lead	AS	1200491	1200506
pgi(25.3E-6)	TAAATACGTTTGTTTGCTTCTTCAC	GACCGTATTTTTTAACTAA	lead	AS	2245042	2245057
ycbI(25.1E-6)	AACTGTTTCCAAGCGACTCCTTCAC	TCATAAATCTTTCTCCTGG	lead	AS	218241	218256
ypfF(25.1E-6)	TATGTGGTTAGTAATGGAACTTCAC	GTGTTTCTCGTCCACGAAT	lag	S	1558390	1558405
carB(24.9E-6)	CCATCACAGATTGCATCGACTTCAC	ATTCACGTCCTTGTAAATA	lead	AS	1399224	1399239
pi214(24.8E-6)	TTTTCGATTCATTGGCAGACTTCAC	ATCGAATCAAGCTGAAAAA	lag	S	1045075	1045090
yqbK(24.8E-6)	ATAGTTACAATACATCATCCTTCAC	AACGGGTCAAGCTTTATTT	lag	S	1623170	1623185
hisI(24.6E-6)	TTTTTACTAGCAATAATCACTTCAC	TTGCTTCTTCACCAACTTT	lead	AS	1237413	1237428
ftsK(24.5E-6)	TAGGGTCAACCATCAGAAACTTCAC	TTGACTAGGCAAAGCTTTC	lead	AS	1705152	1705167
kupB(23.9E-6)	AGATAATTGCCAAGGCAATCAGAAC	CACAATATATGCTCCGTTG	lead	AS	613026	613041
rplN(23.9E-6)	ACGATGACTGCTTTCACAACTTCAC	CTTTTTTAACTGCTCCACC	lead	AS	2164972	2164987

mtsC(23.6E-6)	AAAATGATAATTCTCTCCTCTTCAC	TAGGGGCAGTTTCTTCCGT	lag	S	1350292	1350307
pepDA(23.5E-6)	CAGGACTAGGTGAAGAAGACTTCAC	AACAATTACCCTCCCTTAT	lead	S	249419	249434
ptsI(23.1E-6)	ATTTCAATCATGATACCTACTTCGA	TACCATCTGCAACTGGAAC	lead	AS	121915	121930
ywaI(23.1E-6)	TTGAATTCAGAATTATCTCCACCAC	CATGAATTTTGAATTCATT	lag	AS	2208802	2208817
rpsC(23.0E-6)	TCAAGTTGTTTAGCAATTCCTTCAC	CAACAAGATGAGCGTCCAA	lead	AS	2166372	2166387
ywfF(23.0E-6)	GGATTCAAAGCCTTAACTGCTTCAC	CATAAAGTACATTGGCTTG	lead	AS	2255016	2255031
intergenic(22.7E-6)	GCTCTACCAAGCTGAGCTACTTCCC	GGACATGAAGTGCTTGACG	lead	.	282940	282955
yleF(22.5E-6)	CCCGGACTTTGAGTTTATTCTTAAT	TGAAGTAGGTGATTCAGAA	lag	S	1147818	1147833
yidE(22.2E-6)	AAAATTTTTCCTTTTTGAACTTCAC	AGGTTTGAGCGCCATGGAG	lead	AS	835562	835577
yogA(22.1E-6)	CTTCTATTGGATTCTCCTACTTCCT	ATTCTCGCTGGACTATCTA	lead	S	1460644	1460659
glyS(22.0E-6)	TGTCATTGATGGGCACAAACTTCAC	GAACCTTTTAAACCAGCAA	lag	S	1104472	1104487
ypfF(22.0E-6)	GGCTTATGATGACCCACTTCTTCAC	TAATAAAGATTTCTCTAAA	lead	AS	1558342	1558357
ytgF(21.9E-6)	CGACGCTGCTTTTGTTCTTCTTCAC	GAAGGGTGTAACGGGCTTC	lead	AS	1968538	1968553
ylcC(21.7E-6)	TTTAAAGGGGTTGGAAATACTTCAC	TTTTATATGGAGCGGGAAC	lag	S	1125903	1125918
yceJ(21.6E-6)	AGCGAAATATTTTTAGCGACTTCAC	GAATTAATTTTTTTAGTCC	lead	AS	247326	247341
rmaB(21.6E-6)	TTACCAAAGCCTTTCCAATCTTCAC	CAAACGGAGCTTGTGAGCG	lead	AS	714999	715014
ynjG(21.6E-6)	CTCACGACCTTTAACCCTACTTTAC	AGGTAACAAATTTAAGTCC	lag	S	1394984	1394999
pepP(21.2E-6)	TTTGGGGCTTTTGTTAAGACTTCAC	AGCCATTTTCTGTCACTAA	lead	AS	691432	691447
intergenic(21.2E-6)	ATGCTAACCCCTCCATAAGCTTTAA	TGGTATAATAAAAAGAAAA	lead	.	1133651	1133666
llrA(20.7E-6)	TCAACGGCATGACCATGCTCTTCAC	GACGGAAGAAAGCACGAAC	lead	AS	1639102	1639117
alaS(20.3E-6)	GTGCGGTTGAAAGTTTCTTCTTCAC	GATCAATAATTTGCATGAT	lead	AS	1779915	1779930
yfgQ(20.0E-6)	AAAAATTGCTTCAGATGATTTGGTT	GAAGAAGATATTATTATTC	lag	S	568993	569008
intergenic(20.0E-6)	ATTTTAAGAGATTATCAGTGGTATT	AATCATTTTATCAGACATT	lead	.	966884	966899
ps315(20.0E-6)	CTAAAGAGAGCGCCAAACTCTTCAA	GCGTCATTCCCTTTTTAGT	lag	AS	2023699	2023714
prfA(19.6E-6)	TTATCACGTAAATCTGCTTCTTCAC	GCGATAATTCCATAAAACG	lead	AS	586052	586067
yjaJ(19.6E-6)	TCTCGGGCAAAAACTAAAGCTTGTT	CTCCGTTGATGAGCTGCTT	lead	AS	909737	909752
vacB1(19.5E-6)	TGTAAAAATAATGGTTGAACTTCAC	CATATTTTAGAAGCCATGC	lag	S	968257	968272
ybfD(19.4E-6)	TTATCTTGTTTTTTATCTCCCTACT	GTTGTTATTGGTACAATGG	lead	S	158550	158565
rmlC(19.3E-6)	CCAGAAGCCACAACTGCTTCTTCAC	CAAGATGTTTAGTCCGTCC	lead	AS	200761	200776
yngE(19.2E-6)	AGCTTTCCCTTAACGTGGACTTCAC	CTTCGCTAGGCTCGAGGAG	lag	AS	1365714	1365729
pknB(19.1E-6)	GCGAAAGCACTCTCTGAAACTTCAC	TTACTCAAACAAATACAAT	lag	S	1956462	1956477
ybeF(19.0E-6)	ACTTTTACTTTGCCTTCTTCTTCAC	CTGAGATGCTAAAGTTAAC	lead	AS	153682	153697
gltD(19.0E-6)	ATTTCGGCTGTTTTTCTTCCTTCAC	GGATTGCCCAAATAACCAA	lead	AS	1315143	1315158
yjfJ(18.6E-6)	CGCGTTGTTAATCCGAAAACTTCAC	CATATACTTCAGTAATTCG	lead	AS	959323	959338
yljF(18.5E-6)	CCGCCAGCAAAACCAGCCCCTTCAC	CTGAAGGATAGATTCCTTT	lead	AS	1197029	1197044
rpmA(18.2E-6)	AAAAAAGGTGGGGGTTCTACTTCCA	ATGGACGTGACTCACAAGC	lag	S	1091336	1091351
pdhB(18.1E-6)	GTATAGTAACCTTCAGGAACTTCAC	CTTTAAGTGAGCGATAAAG	lag	AS	62610	62625
celB(18.1E-6)	TTAATAACCAAGCCCTCTACTTCCA	AGTTCACTCACAAGGTATA	lag	S	178484	178499
intergenic(17.8E-6)	ACTTCCATATCCCTTTATACTTCAC	CTTAATAATCTTTTTAATA	lead	.	1987040	1987055
yahG(17.7E-6)	GAGCGATAAACTCTTTCAACTCGGC	AATTTTCTCTTCAGCTTTA	lag	AS	74058	74073
rbgA(17.7E-6)	GATGCTCGTCTCCCTATTTCTTCAC	GTAATCCGATGTTGGGGCA	lag	S	1329921	1329936
ps105(17.6E-6)	CAAAACCTAAAATAGAGGACTTCAC	AGATTATGGCGAAGACGGC	lead	S	39194	39209
lepA(17.5E-6)	GGACGATAATCAGAAATCTCATAAT	CAAAGCTGGCATAACCTTT	lead	AS	1118787	1118802
pppL(17.2E-6)	ACTTGAATATATCTGCTTGAACTTCAC	TAGTTTGCCCAAGTGAACG	lead	AS	1957232	1957247
ybaB(17.0E-6)	AAAATACGAGTTGTCCATTCTTCAC	AGTTAATGACATCAGAAAC	lead	AS	103218	103233
lysQ(17.0E-6)	GGTGCGGGAACCAAAACTGCGTAAT	CAATCCTGCTGTCGTTAAA	lead	AS	369938	369953
yqbK(17.0E-6)	GTTTCTGCAGTACCTCCCATGGCAC	TACAATATACTACTCCAGC	lead	AS	1622667	1622682
ybeF(16.9E-6)	CCATTTTTGTCGGTATCTCCTTCAC	GAATTAATTTATCTTTTTT	lead	AS	152104	152119
eno(16.9E-6)	CCATCTCTTAATTCAACTGCTTCAC	GGTCTCCAGTTGATGCTCC	lead	AS	274299	274314
araT(16.7E-6)	TAAGATAAGCGGACAAATCCTTCAC	CGTACTGACCAAAAGCGAT	lead	AS	57646	57661
pi214(16.7E-6)	AAGTAAGGTTCTCCAATCACTTCAC	CTTTTTCATTCTTAGGATA	lead	AS	1044675	1044690
rpsC(16.7E-6)	CGTGGTGAAGTTCTCCCTACTAAAA	AATCTGTGAAAGGAGAAAA	lag	S	2166072	2166087
comFC(16.6E-6)	CCTTTGGTTCCAATTCCTATTTCAC	CAAAAAGATTAGAGGAACG	lead	S	1096611	1096626
gidC(16.6E-6)	TTGGAAGTTTACCTTACTACATCAC	CCATACTGACAGCAAACAT	lag	S	1257952	1257967
ycgI(16.5E-6)	TTCAGAAGAAAATTTGCAACTTCAC	TTACATTGTGTTGGTGGTT	lag	S	267947	267962
yciH(16.5E-6)	AATATTATTCATCAGGTGTCATAAT	CATTGGAACAACAAGTGGT	lag	AS	289429	289444
intergenic(16.5E-6)	ATCAAGAGAACCATCCCAACTTCAA	AGAAGAGGGCAATCCCAAT	lead	.	2267063	2267078
intergenic(16.4E-6)	TTAAACAAAGTATATTATACTTCAC	ATTACTATGTAAGTCAAGG	lead	.	308448	308463
agl(16.4E-6)	CAGATAAAATCAATCCCCAATTCAC	GTAGATAAGGAATTTTTTC	lead	AS	1732727	1732742
ftsH(16.3E-6)	TAAGCTTGGAAGTTTTTAACTTCAC	TACTAGAATTACCTAAAAG	lead	AS	26157	26172
pmrB(16.0E-6)	CCAAAGGACAATTAAATTTCGTTGC	CATGATTCTTTCATGAATG	lag	AS	130558	130573
ribA(16.0E-6)	ATTAATAAACTTCGTGCCTACTCAC	TTCAAGATGAAGGACTTGA	lag	S	1024702	1024717
argG(15.9E-6)	CAGATTCCACTGCCCCTACTTGAAG	CGCCTTATCATGAATAAAA	lead	AS	126892	126907
purL(15.9E-6)	TCTTCATCCGTCAGTCCCCACTCAC	GGTATATTTTGCTTTCTTG	lead	AS	1576342	1576357
fhs(15.8E-6)	TCGGCCAAATCTAAACCACCTTCAC	CACCCTTAGCAAAGACTTG	lead	AS	960978	960993
llrF(15.8E-6)	GATTATTGACAAATTCTGTATCTTT	CAATTTTTTTCCGAATTTTA	lead	AS	1727031	1727046
yshA(15.6E-6)	CATGCACTTGGACTTTCTACTTCCG	GAGCAGATATGCTTGGTGG	lag	S	1876038	1876053
vacB2(15.4E-6)	TCTAAAATATCTAGTCCTACTTCGC	CTTTATGTCCAATAATACC	lead	AS	1226666	1226681
ysdB(15.4E-6)	TGACCAATTAAGCCCATAACTTCAC	CTGGTTTAACAACCATATT	lead	AS	1835702	1835717
yqgA(15.0E-6)	CTTGAGGTTGACGTGCTTCCATAAC	TACTGGAAGAATCACTGGT	lead	AS	1662046	1662061
infB(14.9E-6)	CCATCATGAATCACGACACCTTCAC	GAATGACACGGACAGAAGC	lead	AS	778425	778440
gltB(14.9E-6)	TTACCTGGCAATTGCCCACCTTCAC	CTGGTTTAGCACCTTGCGA	lead	AS	1318175	1318190
gntK(14.9E-6)	CCCGGGGAAATGTTTGCTGCAAGTT	CCGTGACTTCATCAAAAGA	lead	AS	2269167	2269182
groES(14.8E-6)	CCATGATGATTTGTTTTCCCTTCAC	CGACGGCAACAACTTCAGC	lead	AS	398636	398651
L200015(14.8E-6)	TGAGAAAGTTAAATTCAAACTTCAC	TTGCTTCTCCAAATCTAAC	lead	AS	456625	456640
pi115(14.8E-6)	TGAGAAAGTTAAATTCAAACTTCAC	TTGCTTCTCCAAATCTAAC	lead	AS	456625	456640
fhs(14.8E-6)	ACTGTAATATCAAAACCATCTTCAC	GGGGCACACCATTTAAAGG	lead	AS	960309	960324
nifS(14.8E-6)	AACGCTGCTACAACACCTCTACTTC	CTGATGTTATCACCGCAAT	lead	S	1927818	1927833
accC(14.7E-6)	TCTTGCTTGACAGACAATTCATTGC	CATTGGCTACACGAATCTG	lead	AS	788253	788268
dnaD(14.7E-6)	ACCTTACGATTGAGGACTGCTTCAC	GCAAAGCTTGTTTAATCAA	lead	AS	1083630	1083645
sbcC(14.7E-6)	TTAGCTGTATTTTGTACGACTTCAC	TTAAAGCCAAAGCAATTGA	lead	AS	1353084	1353099
yngG(14.7E-6)	AGCACTCGGAGTTTTCCTACTTCAA	GTTTTCTATCAACAAGGTC	lead	S	1368503	1368518
trmE(14.7E-6)	GATGATGTGGAAACAATGACTTCAC	AAATGCTCCTTGAAAAAAC	lead	S	2328325	2328340
ydaG(14.2E-6)	TGATAATGGCAAACATAATCATTCC	CATATAAGTCATGAATGTA	lead	AS	309995	310010
snf(14.1E-6)	TCTAGGTCATAACTTACCTCTTCAC	CATCAATTTCTGCTGTTAG	lead	AS	2123579	2123594
L200121(14.1E-6)	CAGACTCAAAATCTGGTTCCTTCAC	GGGAGTGCCGGTTCGACCC	lag	S	2340443	2340458
ldhX(13.9E-6)	TGAGCATAAGTACTCCCTACGGCTC	CAGCTCCAACAATTACAAC	lead	AS	1142431	1142446
yohC(13.9E-6)	GAACTCCTTGCTGACCTTCTACTTC	CGGTCCTTTGGCAACATAA	lead	S	1472006	1472021
ywjH(13.9E-6)	AATTCAACTAAACCACTTAAAATTT	CATTAAATTTATCTGGCCA	lead	AS	2299858	2299873
yrfC(13.8E-6)	GCAATACAAATATCTCCATCTTCAC	GTGGCATTGTTCCTTCAAC	lead	AS	1753200	1753215

secA(13.5E-6)	TTCAATTACGATTCAAAACTACTTC	CGGATGTATAAAAAACTGT	lag	S	117488	117503
ftsY(13.5E-6)	CCAGCTGGTTTTGTGACTACTTCAA	CGCCTGAACGATTTCCCCA	lead	AS	825016	825031
trxB2(13.5E-6)	GCATTAATCGCATGGACTGCTTCAC	CAAAACCAACGGACATCAA	lead	AS	1694915	1694930
intergenic(13.4E-6)	CAATGAACCTTTTTGCAAACTTCAC	AAGATGTGAAGGCAAAGAT	lag	.	1702217	1702232
yucF(13.4E-6)	GCTTGCCAAAATTCACCGACTTCAC	TTTCAAACTTTTTCTTAAG	lag	AS	2025914	2025929
purA(13.4E-6)	CGACGGCAAAAAGTTCAAACTTCAC	CTTATTCCATCAGGAATTT	lag	S	2029866	2029881
L200158(13.4E-6)	ATAGAAACTAGCGTCCCTATTTCAA	TGGCTCCTACCTATCCTGT	lead	AS	2361099	2361114
yuaD(13.3E-6)	CGCGCACGCGTCTGATAAACTTCAC	CTTCTGATTCTACATCATA	lead	AS	2005245	2005260
yahG(13.2E-6)	AAAGGCTCAATCCTCTTTGCTTCAC	ATGACCATGAATTCATTAA	lead	S	73337	73352
yohJ(13.2E-6)	GTCATAAATATGTATGATTATTTTC	AAGAAGTTATGAATGTTCC	lag	S	1479677	1479692
ycfF(12.9E-6)	AAGTCTTCAAGGCATTTCCCTACGC	GAAATCAAACTCTCAGTAC	lead	S	256796	256811
ksgA(12.8E-6)	AACCATTCTTCATCTTCTACTTCAA	CCAATGGTGCTTCACGACG	lead	AS	690090	690105
rmeA(12.5E-6)	TCGTTTCAGGATTTCCCTCATTCAC	TAATGCCATATAACGGGTC	lead	AS	1947036	1947051
ygiI(12.4E-6)	TTGGCTGGCATCCTGATGATGCTGC	TGAATTTGATGACCAAGCT	lag	S	687601	687616
yibG(12.4E-6)	AAAAGACCAGCACCAATTGCTCCAC	CAAGAGCAATCATAGTAAT	lag	AS	819874	819889
yljF(12.4E-6)	GAAAAAGTTCCTGCTCCACCTTCAC	CAAATTGAACATTCGATTT	lead	AS	1195988	1196003
oppA(12.3E-6)	TAGTCAAGCGTCATCCCTACCACAC	GTTTGTTAACTGGGGTGTA	lead	AS	1905177	1905192
yciH(12.2E-6)	AGTGGGTGAACCTGCTGACCTTCAC	CGAATGGCTGAACTTGGAA	lead	S	290530	290545
gltB(12.2E-6)	CGTTTTGCATTTTCACCACCTTCAC	CCGAATTGGATTTTGCACC	lead	AS	1318322	1318337
yneG(12.2E-6)	GAACAGTCCTGACAGTCTTCTTCAC	ATCCGAGATTTTGAAAACT	lead	AS	1345665	1345680
llrC(12.1E-6)	TTAAATGCTTCAAGAGCTTCTTCAC	CATCAAAAGCTGTAAAGAC	lag	AS	403590	403605
ypcD(12.1E-6)	GACGGAGTTACAATTATTCCTTCAC	CAGATGAGCCACCCCAATA	lead	AS	1524368	1524383
clpB(12.1E-6)	TGACCAAGCTAATCGTCGCTTGATG	CAATTAGAAATTGAAGAAG	lag	S	1567980	1567995
yijB(12.0E-6)	CCAATGAAAAAGCCACCTACTCCAC	CAACAATTAATGTTAGTAA	lead	AS	892941	892956
yniC(12.0E-6)	CGATAAAATGAAAAAATTACTTCAC	TTTTTGGTTGTGTTACTTG	lag	S	1381911	1381926
yniH(12.0E-6)	ACAGCTCCTTGGACAAAACCTTCAC	GGATAGAAGGGTCACCAAG	lag	AS	1387638	1387653
ycfD(11.9E-6)	ATGATTGTCCTCGACCCTCCTTCAT	TTGCCAGAAATGGTAAAAA	lag	S	253569	253584
ispA(11.7E-6)	GCTAATGTCAACTCTTTTCCTTCAC	CAGCCATATCCAAGATTTG	lead	AS	881208	881223
tpx(11.6E-6)	TCAGAAAGCTCTGACAAAACTTCAC	TATAGACAATTTGACCATT	lead	AS	302128	302143
yeeA(11.6E-6)	CCATAAGAACGTTTCCCTACTTCTT	TTTTATCATATTGGATTAT	lead	AS	439614	439629
ybdC(11.4E-6)	TTTTCGACCTGTACCTTAGCTTCAC	GCGCCGCAATAATTTTGTA	lead	AS	132561	132576
yvhB(11.4E-6)	TTCATTATTTTTTCACCGCCTTCAC	CCCATGACCAACGAGTGAT	lag	S	2175732	2175747
yafC(11.3E-6)	GAGTCACTTACTATCTCTACCTCAC	TCAAAAGACCGTGACCAAC	lead	AS	55779	55794
araT(11.3E-6)	TTAATAATCTGTGCCACTAATTCAC	GCGCTGCAAAGATTAATCC	lead	AS	57325	57340
